# Ribosomal frameshifting and transcriptional slippage: From genetic steganography and cryptography to adventitious use

**DOI:** 10.1093/nar/gkw530

**Published:** 2016-07-19

**Authors:** John F. Atkins, Gary Loughran, Pramod R. Bhatt, Andrew E. Firth, Pavel V. Baranov

**Affiliations:** 1 School of Biochemistry and Cell Biology, University College Cork, Cork, Ireland; 2 School of Microbiology, University College Cork, Cork, Ireland; 3 Department of Human Genetics, University of Utah, Salt Lake City, UT 84112, USA; 4 Division of Virology, Department of Pathology, University of Cambridge, Hills Road, Cambridge CB2 0QQ, UK

## Abstract

Genetic decoding is not ‘frozen’ as was earlier thought, but dynamic. One facet of this is frameshifting that often results in synthesis of a C-terminal region encoded by a new frame. Ribosomal frameshifting is utilized for the synthesis of additional products, for regulatory purposes and for translational ‘correction’ of problem or ‘savior’ indels. Utilization for synthesis of additional products occurs prominently in the decoding of mobile chromosomal element and viral genomes. One class of regulatory frameshifting of stable chromosomal genes governs cellular polyamine levels from yeasts to humans. In many cases of productively utilized frameshifting, the proportion of ribosomes that frameshift at a shift-prone site is enhanced by specific nascent peptide or mRNA context features. Such mRNA signals, which can be 5′ or 3′ of the shift site or both, can act by pairing with ribosomal RNA or as stem loops or pseudoknots even with one component being 4 kb 3′ from the shift site. Transcriptional realignment at slippage-prone sequences also generates productively utilized products encoded *trans*-frame with respect to the genomic sequence. This too can be enhanced by nucleic acid structure. Together with dynamic codon redefinition, frameshifting is one of the forms of recoding that enriches gene expression.

## INTRODUCTION

**Figure 1.**
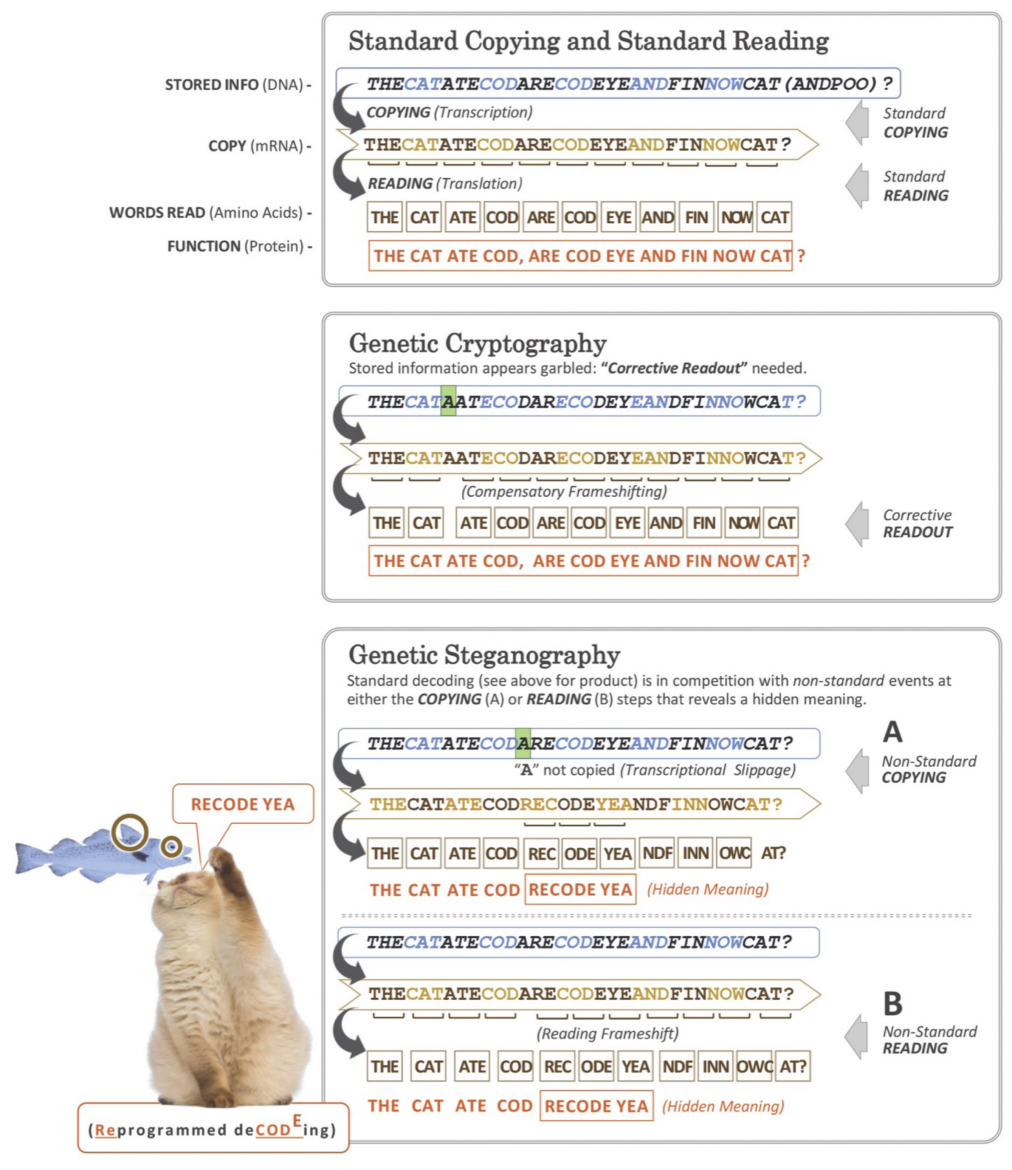
Genetic ‘Bletchley-ism’: As illustrated with three letter words, the framing of genetic informational readout can be modified to convey meaning from genetic ‘hieroglyphs’ (cryptography) or additional and hidden meaning (steganography). Embellishing the old adage ‘From tapes to shapes’ (proteins), in several cases this involves ‘shapes-in-the-tapes’ unlike counterparts in many human languages. The process is dynamic, and the competition yields products from both standard reading and frameshifted reading. The relative proportions of the products from each are case dependent. Examples of genetic cryptography involving translational bypassing are in the decoding of phage T4 gene *60* and the mitochondrial genome of the yeast *Magnusiomyces capitatus* (56,65,158) and another type is in decoding the mitochondrial genome of glass sponges (252). The latter is a WT translation component counterpart of the suppression of frameshift mutants by suppressor mutants of translational components. Examples of genetic steganography involving transcriptional realignment are in the gene expression of paramyxoviruses, potyviruses and the bacterial insertion sequence *Roseiflexus* IS630 (42,99,617); examples of genetic steganography involving ribosomal frameshifting are in the decoding of influenza A virus expression (125,270) and *D. melanogaster* APC (46). While standard expression of most bacterial release factor 2 genes, and also probably eukaryotic antizyme genes except for antizyme 3, yields a product that is non-functional on its own, the +1 frameshifting required for productive expression has been positively selected. The representation was inspired in part by a genetic framing garden ‘rebus’ (812), a slide by V.N. Gladyshev and a recent publication (1).

AUG. Doubtless applicability of the word ‘steganography’ to certain forms of genetic recoding and frameshifting in particular, was not envisaged when it was first used in 1499 to mean an intended secret message that does not attract attention in contrast to cryptography where just the contents of the hidden message is protected and not its existence. Nevertheless, its use in connection with productively utilized frameshifting by Patrick Moore (1) highlights the extra N-terminally coincident product(s) whose synthesis involves a switch from the frame set at initiation to one of the two alternative reading frames (registers) inherent with standard non-overlapping triplet decoding (Figure 1). The frameshift-derived product is generally quite different in both length and sequence from the product of standard decoding. It is not only ribosomal frameshifting that can yield a *trans*-frame encoded protein, but also where the RNA polymerase ‘slips’ to yield mRNA lacking or containing one or more extra bases (that are not 3 nt or multiples thereof). Such ‘transcriptional frameshifting’ also yields products that are *trans*-frame specified with respect to sequence present in the encoding DNA (or RNA in the case of some viruses).

To commemorate this year the 50th anniversary of the full-deciphering of the genetic code and the 100th anniversary of Crick's birth, we provide an overview of knowledge gained since then on the aspects of the dynamic nature of both mRNA generation and code readout gained by studying frameshifting, especially ribosomal frameshifting. For space reasons, other features of the ‘extra layer’ in code readout, including dynamic codon redefinition and other processes that yield a *trans*-frame encoded product with respect to the DNA will generally be omitted (even though certain RNA processing (2) involves a ribosome/nascent chain complex, indel editing can have similar consequences to RNA polymerase slippage, and of course splicing is of major importance). However, tmRNA which has been recently reviewed (3,4) will be treated minimally.

Many cases of utilized frameshifting are phylogenetically conserved, thereby facilitating their identification bioinformatically though this is most feasible when the ribosomes that shift frame do not encounter a stop immediately in the new frame but continue translating in the new frame to synthesize an extensive C-terminal protein segment. While increasing sequence information has of course helped, improvements in bioinformatics software has been crucial, especially in its application to RNA viruses.

Productive frameshifting is generally in competition with standard decoding. At the functional level there are three broad classes. In many cases the proportion of ribosomes that shift frame, or of polymerases that slip with consequent frameshifting with respect to their template, is constant (though in some cases this may reflect our ignorance of a relevant regulatory condition). This is often termed ‘set ratio’ frameshifting and the function is commonly generation of an extra N-terminally coincident product. In a second class, frameshift efficiency is responsive to the level of initiation or a trans-acting factor. In this class the frameshifting acts as a sensor and effector for regulatory purposes, either via synthesis of a functional trans-frame encoded product, mRNA half-life or new frame ribosome movement affecting translation of a downstream ORF, e.g. by affecting mRNA structure and initiation site accessibility. A third functional class is ‘corrective’ frameshifting where the effects of a ‘problem’ indel, at the DNA level (or potentially at the mRNA level, e.g. U-indel editing) are translationally compensated. Most known occurrences of this class are in mitochondria.

However, a different type of example is the combination of a ‘savior’ indel together with a low level of compensatory frameshifting. For example, an indel that adds or deletes a G in a run of 7Gs in herpes virus thymidine kinase mRNA prevents susceptibility to a common antiviral drug. A very low level of frameshifting in decoding the run of Gs yields sufficient product to allow reactivation from latency but not enough to activate the drug inhibitory pathway (5,6) review (7) (‘savior’ being from the virus, and not the patient's, perspective). With ‘compensatory’ frameshifting, in general, the non-shift derived product is useless and degraded. Though use of the words corrective or compensatory would be questionable, from a mechanistic and practical perspective, the ability of a high proportion of ribosomes to simply ignore a synthetic unnatural base, and continue translation as if the base were not present (8), is also pertinent.

Some cases of ribosomal frameshifting are more detectable in specific cell types, e.g. where the amount of a particular aminoacyl-tRNA is low, at certain stages of growth, e.g. bacterial stationary phase, or in a particular phase of a viral infective cycle. Other novel cases are claimed in yeast under oxidative stress (9), in the context of broader translational adaptations (10). An extreme in this regard is the retention over long evolutionary time of the ability of *S. cerevisiae* release factor, eRF3 to convert to its [*PSI+*] prion form and ribosomal frameshifting associated with it (11–13) that may have survival value under stressful conditions and create the opportunity for a later mutational change to genetically fix the newly advantageous trait (14–16). Intriguingly, [*PSI+*] induces synthesis of a substantial amount of a *trans*-frame encoded variant from the gene for eIF1, and specific other translation initiation, tRNA maturation and amino acid metabolism genes (13). In other cases, even when high level, frameshifting can be effectively inconsequential. In some, the signals promoting the high level were selected for a different purpose, and efficiency of degradation of ‘dangling tail’ C-terminal extensions is also relevant (17–19).

### mRNA stabilization and destabilization

Frameshifting selected due to productive utilization of its derived protein product generally results in a proportion of ribosomes terminating on sequence on which ribosomes in another frame are continuing downstream translation. To an unknown extent, selection has presumably operated to generate features for avoidance of mRNA instability associated with the terminator in either of the utilized frames that is closest to the start codon. Avoidance is likely most relevant when the great majority of ribosomes are in the frame that leads to termination at the first terminator in either frame. Given the complexity of mRNA degradation and difference between the major classes of organisms, there is probably a diversity of answers with cytoplasmic transcription for many RNA viruses also being relevant. Parallels to the diversity of stabilities of internal UGA-containing selenoprotein mRNAs may emerge. However, attention is drawn to a sequence shortly after a frameshift site and 3′ adjacent to a relevant stop codon, that binds a polypyrimidine tract binding protein that competes with UPF1 (20,21), a component of the nonsense mediated decay system. A more questionably relevant case involves the very different organization of an RNA virus genome whose decoding involves shifts into both alternative frames (22,23).

The opposite type of situation is where frameshifting is advantageous because it leads to mRNA decay. Numerous high level frameshifting candidates for this have been identified (24–26). Many are not phylogenetically conserved, making it difficult to assess overall significance especially as the situation is different from where selection acts via the protein product. However, in some cases experimental analysis has yielded provocative insights as, for instance with the human CCR5 cytokine receptor that acts as a HIV-1 co-receptor with implications for other cytokine receptor mRNAs, especially interleukin receptor subunit mRNAs (27,28). This particular example is present from humans to lemurs. As a counterpoint to consequences of termination by frameshifted ribosomes, there can be dramatic effects of not having any stop codon in the new frame, as illustrated by a viral case (6). On bacterial mRNAs lacking stop codons, tmRNA-mediated shifting of translation onto its own internal sequence is associated with both mRNA degradation (29) as well as abberant protein destabilization. (Utilization of frameshifting to lead to the unwinding of stem loops to permit downstream initiation is dealt with below.)

### Framing fidelity

As described below, frameshifting is dependent on codon combinations and the physiological state of the cell, and so estimates of frameshifting levels without any productive consequence are only broad generalizations (the initial estimates were extrapolations from the extent of leakiness of frameshift mutants). The consequences of framing infidelity in synthesizing such giant proteins as titin is greater than that for small proteins because of loss due to premature termination by frameshifted ribosomes, but there is currently no evidence for extra long ORF framing fidelity enhancement. In contrast many missense errors are inconsequential. The other extreme is the possibility of ribosomal frameshifting being frequent and ∼100% efficient. The best candidates for this occurs in the decoding of the ciliate *Euplotes* (30–35).

## EVOLUTIONARILY SELECTED SITE-SPECIFIC FRAMESHIFTING

Here for practical reasons, the focus will be on discrete cases of frameshifting for which evolutionary selection is evident. This is not a value judgement since the importance of several described above is obvious and newly derived frameshifting could play a critical role in the species in which it evolved.

While transcriptional and translational frameshifting are distinct, there may well be sites at which both occur. With both there is a shift-prone site (‘slippery’/‘shifty’ site) at which the non-standard event occurs. In some instances, e.g. in decoding certain tailed phages, influenza A virus and likely closteroviruses (36–41), and maybe antizyme 3 (see below), the ribosomal shift seems not to involve stimulatory signals and despite its low efficiency leads to the synthesis of important proteins. An example with polymerase realignment for low efficiency but high importance frameshifting occurs with potyviruses (42–44). For both transcriptional and translational frameshifting, the efficiency of the non-standard event is often greatly enhanced by stimulatory (recoding) signals, though current knowledge of such signals for transcriptional realignment is very limited. Nevertheless, very elaborate recoding signals are present sometimes, even when only low efficiency frameshifting occurs, for instance in several mobile elements (45), but also for adenomatous polyposis coli (APC) (46).

The shift from the initiation-set frame can be to either alternative frame. Most ribosomal frameshifting occurrences studied have involved shifting −1 (1 mRNA nt toward the 5′). Occurrences that do not involve an overlapping codon, are commonly referred to separately as bypassing or hopping. With bypassing, coding resumption can be set to occur in any frame, even the zero frame. It is briefly included here because of some shared mechanistic features.

Related aspects of frameshifting are considered together, and consequently different features of any one case may appear in different sections. This necessitates some repetition, but it is kept to the minimum necessary to permit side-by-side comparison of the individual features. Many insights have come from the study of reporter-containing cassettes. Now however, the ability to analyze frameshifting in more natural contexts, greatly expanded sequence information, bioinformatic advances, ribosome profiling, biophysical techniques enabling single molecule studies and structural information from cryo-electronmicroscopy, creates an opportune time for a new survey. Discerning how ribosomes and RNA polymerase sense and respond to recoding signals is at last becoming approachable, but is just part of the broader issue of what a pioneer of deciphering the genetic code termed ‘remarkable and quite beautiful recoding mechanisms’ (47).

Selection to avail of the advantages of frameshifting has been particularly active in the evolution of viral, and other mobile element, genes – some of which are very important. So far, at least, the number of known evolutionarily conserved instances in the decoding of chromosomal genes that are not mobile elements or derived from them, is small.

Any particular case of programmed frameshifting is generally specific in terms of directionality, though there are some evolutionarily conserved exceptions (e.g. the tailed double-stranded DNA phages where either −1, −2 or +1 is used in different phages to yield a ratio of two products important for tail length and assembly (40,41,48,49). Most known instances of ribosomal frameshifting mediating access to the +1 frame are by +1 frameshifting, but −2 frameshifting is known and results in specification of an additional amino acid compared to +1 frameshifting. −2 frameshifting is used in decoding phage Mu (50,51), arteriviruses (nsp2TF), including an important pig pathogen where the efficiency is 20% (22) and *Trichomonas vaginalis* virus-1, a virus that infects an important human genitourinary protozoan parasite (52), perhaps with the nature of potential frameshifting in its host *Trichomonas* (53) being relevant. [However, *Trichomonas vaginalis* virus-2, -3 and -4 all utilize −1 frameshifting instead of −2.]

A big majority of ribosomal frameshifting occurrences, especially −1, involve dissociation of P-site tRNA anticodon: codon pairing and realignment with re-pairing to mRNA at a new and overlapping codon. Frameshifting involving re-pairing at a non-overlapping new frame codon (hopping/bypassing), (54–56) appears much less frequent. When a ‘stop hop’ occurs with 9 nts encoding a single amino acid (54), the distinction at the product level from stop codon readthrough is two fewer amino acids.

Until recently utilized −1 frameshifting was only known to yield a fixed set ratio of the product of standard decoding to the frameshift-derived product. However, it is now clear that as with several cases of +1 frameshifting, some −1 frameshifting is also responsive to *trans*-acting components and is regulatory.

### Position(s) within coding sequences: ORF architecture and relationship to efficiency and function

Translation of only a short zero frame sequence can be adequate to permit the utilization of frameshifting that acts as a sensor and effector of a regulatory circuit. The frameshifting can result in zero frame product being fused to functionally important domains encoded by the new frame. With *E. coli* release factor 2 mRNA, the functionally important part of the product is synthesized from the new frame by frameshifted ribosomes (refs. below). Though it has not been experimentally investigated, there may be counterpart −1 frameshifting early in the decoding of phage λ cI mRNAs where it is the short frameshift product that is rapidly degraded and the act of its synthesis serving as a ‘ribosome sink’ (57), perhaps analogous to that, probably for a different regulatory purpose, in decoding some cardioviruses (58,59). In contrast, the function of frameshifting can be unrelated to products of the overlapping ORFs on which the frameshifting occurs. Though long postulated (60), a bacterial case has recently been discovered where translation of a short zero frame leads to ribosomes frameshifting and their continued progression in the new frame affecting significant mRNA structure. In this instance, the consequence is allowing initiation of synthesis of an important downstream encoded protein (61).

Several examples will be used to illustrate frameshifting in central regions of coding sequences. No ORF overlap is involved where ribosomes encounter a stop codon as the first codon in the new frame; the frameshift-derived product lacks C-terminal ‘domain(s)’ present in the product of standard translation. Where the efficiency of transition to the alternative frame is close to 50%, a single nucleotide change can reverse the roles with longer product being derived from frameshifting almost at the end of the ORF yielding the shorter product. An example is bacterial *dnaX*, see below, which illustrates the relevance of efficiency to location. With *D. melanogaster* APC, the new frame encodes a functionally significant C-terminal segment that of course differs from that of the product of standard decoding, though they share the same N-terminal segment (46).

Frameshifting near the end of an ORF encoding a structural protein to yield a fusion with an enzyme required in lower quatities, is typically at a low level as illustrated by HIV GagPol. In such cases, the potential for mutationally switching the downstream ORF to the zero frame cannot be simply accomplished, with retention of frameshifting for expression. However, it can occur with utilization of a different type of recoding. With a different retrovirus, Moloney murine leukemia virus, the downstream *pol* ORF is accessed by a few per cent of ribosomes reading through the *gag* terminator with near-cognate decoding of the stop codon. While some retroviruses utilize just a single such −1 ribosomal frameshift, certain others utilize two −1 frameshift events (62) and in these the first one is more efficient. At least two frameshifts occur in the decoding of one *Euplotes* gene (32,63), one mitochondrial gene has 10 (64) and the current champion gene which involves 12 reading frame shifts is also mitochondrial (56,65).

## OCCURRENCE AND FUNCTION IN MOBILE ELEMENTS, DERIVATIVES AND SECONDARY GENOMES

### Selected viruses

Though viruses are not known to encode their own ribosomes, they are masters at customizing their host's translation machinery to optimize their own gene expression. Increasing coding capacity from small genomes with expression occurring in relevant ratios and with appropriate timing, has doubtless contributed to viral utilization of frameshifting. Frameshifting-mediated polyprotein generation involving fusion to capsid components prior to later cleavage, can aid viral packaging of the viral polymerase (e.g. for retroviruses and totiviruses, but not positive-sense single-stranded RNA viruses which don't package their polymerase). Some eukaryotic RNA viruses use polyprotein synthesis, in conjunction with IRES initiation, as part of their strategy to circumvent 5′-end dependence of canonical eukaryotic translation initiation, allowing its inactivation to shut-off host protein synthesis.

In vertebrates deeply conserved −1 frameshifting is not known to be utilized in non-mobile chromosomal expression despite the vertebrate translation machinery facilitating high efficiency viral −1 frameshifting. Exceptions are the chromosomal genes described below that are derived from mobile genes. Presumably the one now known to be essential was not always so. Given the intensity and complexity of the ‘arms race’ with viruses, it may mean that vertebrate cells have been unable to inhibit such −1 frameshifting, or have always used it themselves for an important function(s), or both.

Many viruses, especially RNA viruses, have small to modest sized genomes, and have extensively availed of decoding versatility. Though the report (66) that the first identified overlapping gene in an RNA virus (67,68) required frameshifting for its expression was incorrect (69), RNA viruses have nevertheless been a rich source of productively utilized frameshifting. However, despite breakthrough discoveries of frameshifting with many retroviruses and plant viruses, and even with intensive study over decades, it is only recently that frameshift utilization has been found in many important small to medium sized RNA viruses. This advance has depended on the development of relatively new comparative genomic algorithms to discern overlapping functional elements embedded within protein-coding sequences (70–72). RNA virus sequences diverge rapidly and, for medically or economically important species, there are frequently many sequenced isolates available, enabling (often even within a single virus species) the statistically significant detection of very short overlapping coding sequences and the RNA elements that direct their translation. The advantages compared to earlier algorithms are most obvious when the overlapping ORF is short and so more difficult to discern.

**Figure 2.**
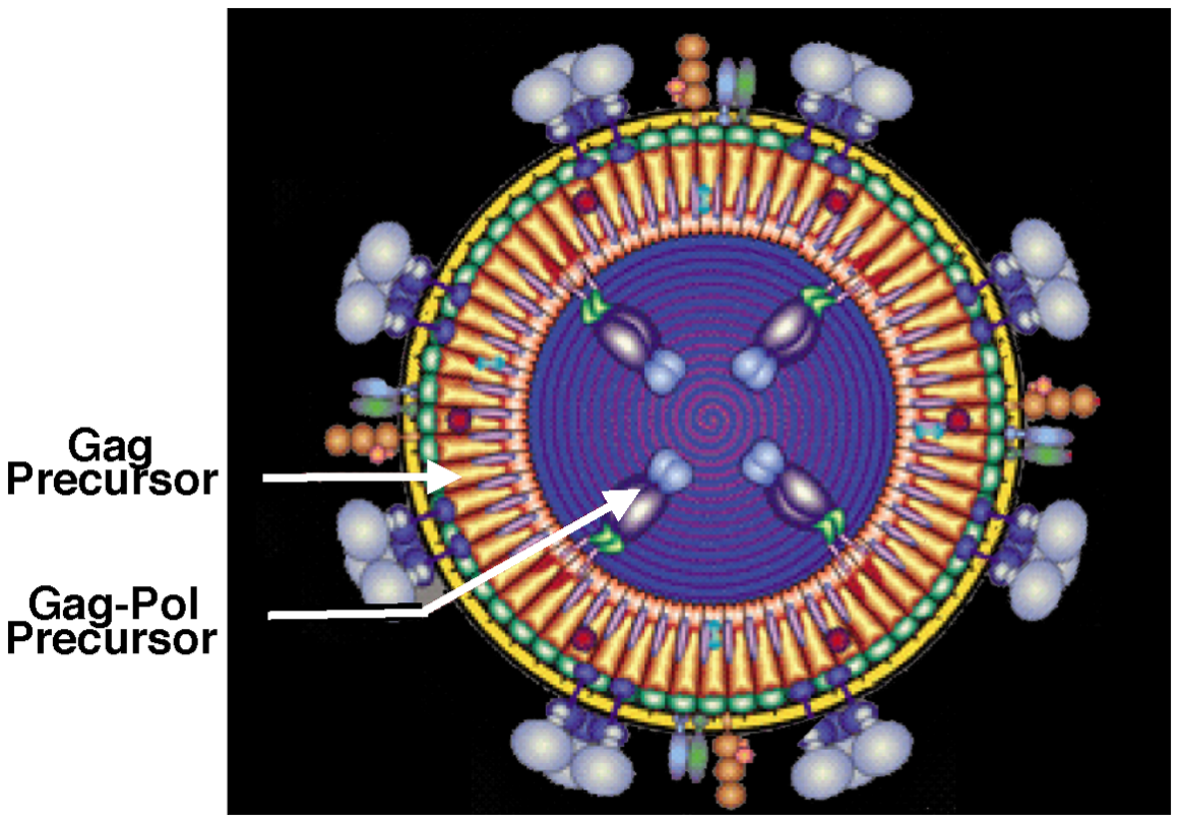
Illustration of the Gag component of GagPol serving to incorporate and localize Pol within forming virions.

**Figure 3.**
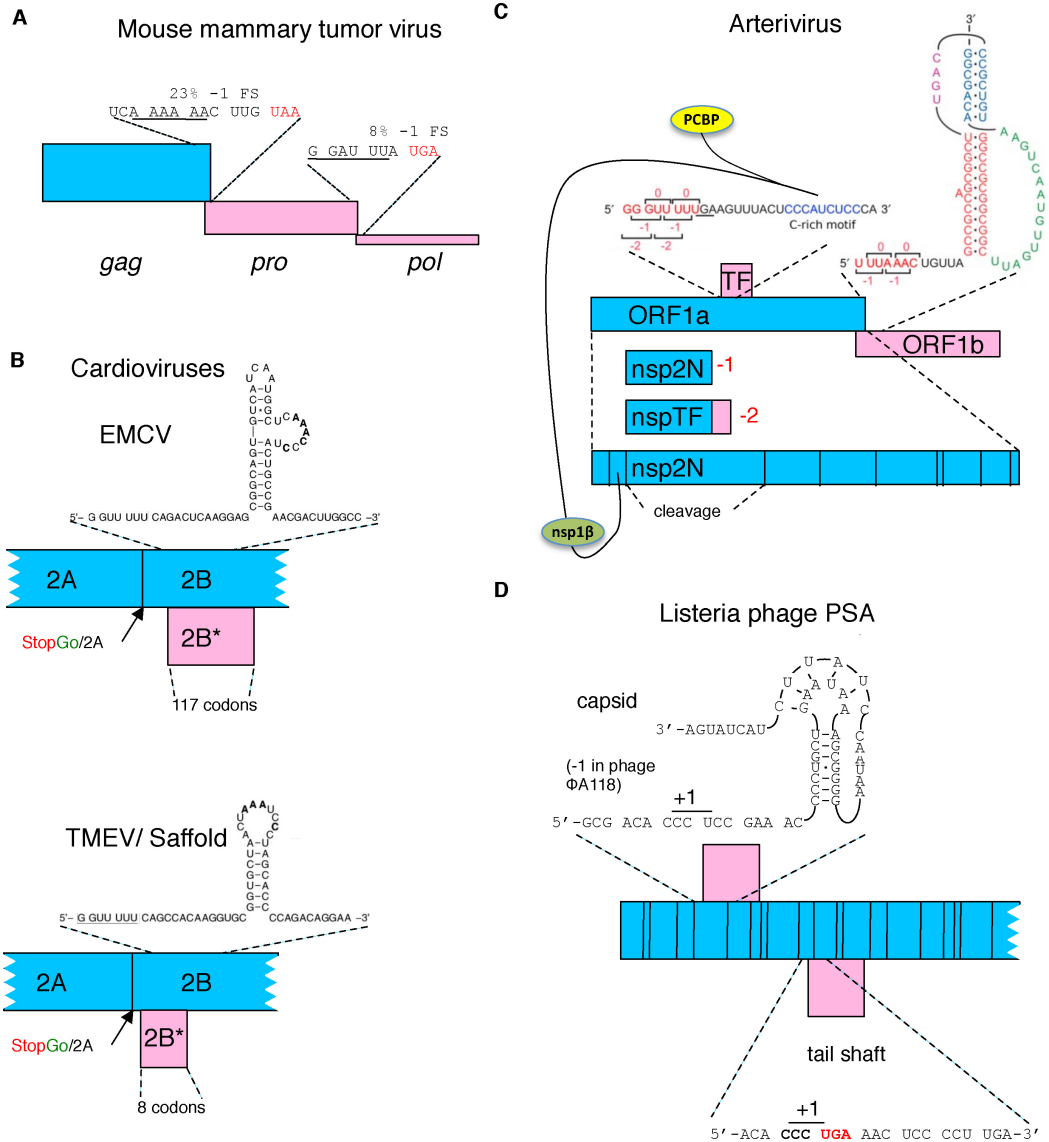
Schematic representation of several viral frameshifting cassettes. (**A**) Retroviral frameshifting with the thickness of the *pro* and *pol* ORFs (pink) reflecting frameshift efficiency and the proportion of ribosomes that decode them with respect to zero frame *gag* (blue). (**B**) The −1 ribosomal frameshift site in two cardioviruses that yields a transframe encoded protein (pink). The proximity of the Theiler's murine encephalomyelitis virus (TMEV) shift site to the StopGo is evident and the relevant amino acid sequence is shown in Table 2. (**C**) In arteriviruses, −1 ribosomal frameshifting near the end of a long 5′ gene, ORF1a, leads a proportion of ribosomes to continue synthesis by decoding a second and also long gene (ORF1b), with the products of both ORFs specifying non-structural polyproteins. At least eight shorter 3′ ORFs encode structural proteins. −2 ribosomal frameshifting in a central region of ORF1a causes some ribosomes to access the wholly overlapping +1 frame TF ORF to yield a C-terminal extension to nsp2 (the product liberated by proteolytic cleavage from that region of the polyprotein encoded by ORF1a). This frameshifting is stimulated *in trans* by virus-encoded nsp1β in complex with poly(C) binding protein (PCBP). (**D**) *Listeria* phage PSA. Capsid and tail shaft encoding genes utilize +1 ribosomal frameshifting on a proline codon just 5′ of either a stimulatory pseudoknot (capsid) or stop codon (tail shaft).

Despite early work with phages MS2 and T7 (73–75), it was the discovery by Jacks and Varmus of ribosomal frameshifting near the end of the *gag* gene of the **alpha retrovirus**, Rous sarcoma virus, that sparked widespread interest in viral frameshifting (76). A total of 5% of ribosomes translating *gag* shift to the −1 *pol* reading frame to synthesize the GagPol precursor that upon cleavage is the source of reverse transcriptase, an endonuclease and a specific protease (Pro) (Figure 2). Those ribosomes that do not frameshift quickly terminate at the end of *gag*. The double-stranded RNA totivirus, L-A, present in killer strains of the yeast *S. cerevisiae*, and a variant L-A-lus in wine yeasts, use counterpart frameshifting (the effects of the killer toxin were originally noted by L. Pasteur). Its efficiency is substantially lower (77–81). Red clover powdery mildew-associated totiviruses have a related frameshift cassette (82). A −1 frameshift event is also involved in the synthesis of the HIV 1 and 2 GagPol precursor. With all these retroviruses, the viral encoded protease involved in polyprotein cleavage is encoded from the 5′ part of the *pol* gene and so is in-frame with the reverse transcriptase encoding sequence. However, in both mouse mammary tumor virus and human T-cell lymphotropic retroviruses (83) (beta- and deltaretroviruses respectively), a second frameshift event occurs at the end of the protease encoding sequence*, pro*, permitting entry to the third and *pol*-encoding frame. This results in the synthesis of both GagPro and GagProPol precursors. The overall efficiency of GagProPol still has to be ca. 2% to 5% (for one at the low end of this range, the efficiencies of the respective frameshifting events are 23% and 8% (62,84) (Figure 3A). The defective endogenous retroviruses, Intracisternal A-type particles (IAP) that accumulate in endoplasmic reticulae, also use two efficient separate frameshift events to synthesize their GagProPol (85,86). Human endogenous retrovirus (HERV), which is highly expressed in some cancers such as colorectal carcinoma (87), utilizes efficient frameshifting (88). Having the reverse transcriptase being part of a Pol precursor with the Gag capsid components, facilitates its packaging into virions and in an inactive form (89). [It has been proposed that HIV Gag-Pol frameshifting is linked to selective viral encapsidation (90), but this has been challenged (91).

Shortly after the discovery of splicing in 1977, it was widely suspected that it was involved in generating an mRNA from which GagPol was synthesized by standard decoding. However, suggestive evidence was quickly obtained that for the gammaretrovirus, Moloney murine leukemia virus, whose *pol* is in the same frame as its *gag*, stop codon readthrough was involved and this was confirmed in 1985 (92,93). By 1984 two groups were testing whether frameshifting was involved in retroviruses where *pol* is in the −1 frame with respect to *gag*. A dilemma faced by both groups at the time was how to distinguish between a low level of ribosomal frameshifting and polymerase slippage (related to acknowledgements in (76)). The initial ‘proof’ for retroviral *gag*- frameshifting being ribosomal rather than due to standard decoding of altered RNA arising from polymerase slippage, was the reported absence of fusion-derived product in *E. coli* cell-free protein synthesis programmed with SP6 transcripts that yielded fusion product in reticulocyte lysates (76). However, cassettes with the *gag-pol* shift region do exhibit frameshifting in such systems with ∼40% of the efficiency shown in mammalian systems (94–98). Ironically for one of us, current *in vitro* work is revealing a modest level of HIV reverse transcriptase realignment (slippage) at the same 7 nt shift site at which the ribosomal frameshifting that yields GagPol occurs, to yield product with an extra base or lacking a base. This polymerase slippage is influenced by the ratio of two of the 4 dNTPs (C. Penno, P.V.B. and J.F.A. unpublished) (and possibly by an RNA structural feature different from that recently described (99)). dNTP depletion is part of the hosts response to viral infection and lentiviruses, such as HIV-2, have developed a *vpx* gene to counteract this effect (100,101). The problem with the control in the initial published work on retroviral frameshifting (76) does not detract from its great importance and the elegance of both it and the subsequent work by the same group including the discovery of heptanucleotide −1 frameshift sites to which 2 tRNAs could pair in alternative frames (102). A similar comment also pertains to the relatively recent finding that a proportion of the HIV frameshifting products are due to −2 rather than −1 frameshifting (98).

**Coronaviruses**, which have positive sense single-stranded RNA genomes, utilize high level −1 frameshifting for synthesis of their polymerase (103–105). Study of infectious bronchitis viral sequences by Ian Brierley and his colleagues led to the discovery of one of the most important frameshift stimulatory elements (106–108). Subsequent studies with both human coronavirus 229E (109) and SARS coronavirus (see below) revealed unexpected additional aspects.

Notable also was the novel distal stimulatory element discovered by W. Allen Miller and colleagues from study of the frameshifting utilized by the **luteovirus**, barley yellow dwarf virus. This led to the attractive proposal that the frameshifting utilized by some RNA viruses to synthesize their polymerase is part of a ‘traffic control’ strategy. The newly synthesized RNA-dependent RNA polymerase proceeds from the 3′ end to synthesize negative strands and soon disrupts the long distance pairing of the frameshifting recoding signals. Not only does this greatly reduce synthesis of now unneeded further polymerase, it frees the polymerase coding sequence for polymerase progression unhindered by oncoming ribosomes (110).

Many other viral genera also utilize −1 frameshifting in synthesis of their replicase (Table 1). An example being members of the ***Astroviridae*** family which, like members of the *Caliciviridae* and *Picornaviridae*, have non-segmented, single-stranded, positive-sense RNA genomes (111,112). In humans, they are important causes of childhood gastroenteritis. Others use +1 or −2 frameshifting for the same purpose, e.g. *Leishmania* virus 1, and *Trichomonas vaginalis* virus 1 (family ***Totiviridae***, with other members of the same family using −1 frameshifting or reinitiation to express polymerase). As the efficiency of this +1 frameshifting can be low, its experimental analysis is sometimes difficult. Curiously, different members of the family ***Closteroviridae***, plant viruses with among the largest RNA genomes, appear to use shifting to the +1 frame at different distances from the terminator, to synthesize their RNA dependent RNA polymerase but further work is needed (113,114) and contrast in (115).

**Table 1A.** Known and predicted occurrences of ribosomal frameshifting in virus genomes. In column S, ‘+’, ‘-’, ‘ds’, and ‘rt’ indicate positive-sense single-stranded RNA, negative-sense single-stranded RNA, double-stranded RNA, and retro-transcribing viruses, respectively

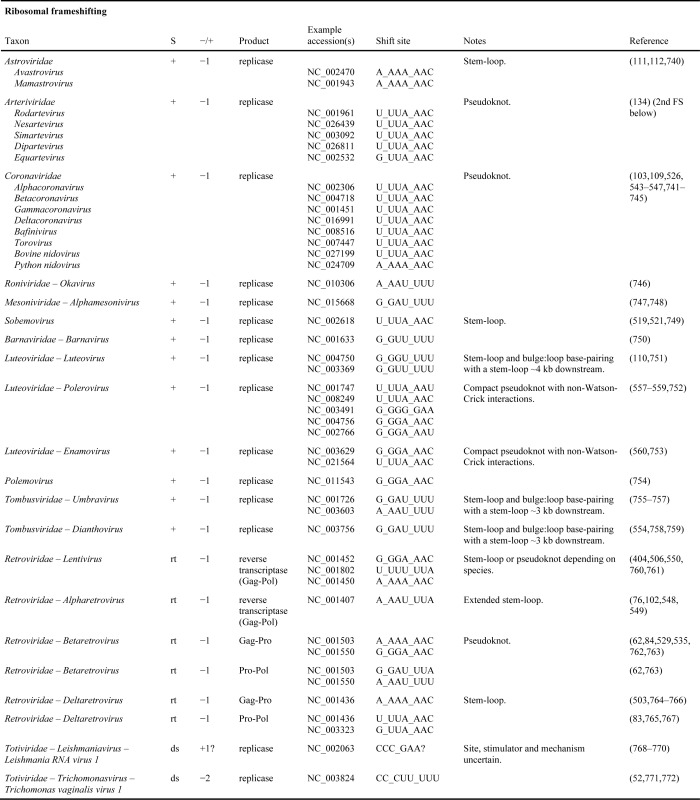							
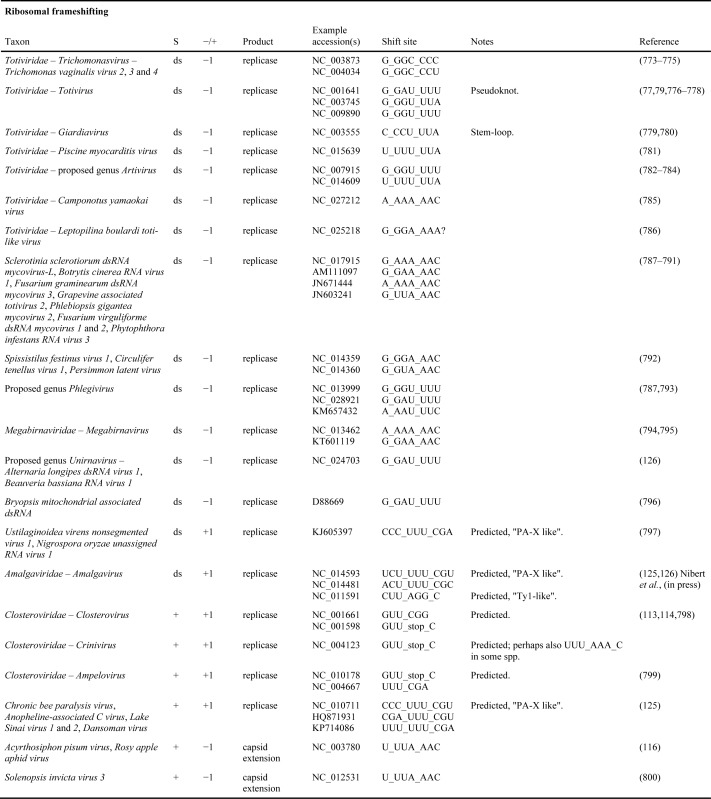							
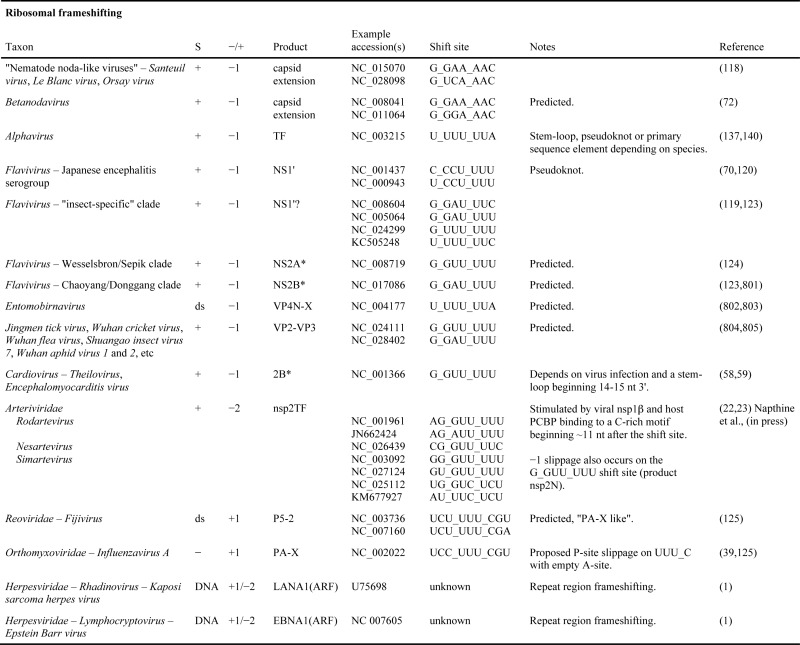							

In **cardioviruses**, the polymerase is encoded in-frame with upstream structural protein coding sequence, and frameshifting is utilized to divert excess ribosomes from decoding polymerase. This is illustrated by encephalomyocarditis virus (EMCV) whose small positive-sense single-stranded RNA was widely used as a model mRNA in early eukaryotic cell-free protein synthesis studies. Its −1 frameshifting mediates synthesis of a functionally important protein that has just 11 or 12 N-terminal amino acids encoded by the zero frame and 117 amino acids encoded from the new frame ‘internal’ ORF (58) (Figure 3B). For the related Theiler's murine encephalomyelitis virus, the *trans*-frame encoded protein is just 14 amino acids, 6 encoded by the zero frame and 8 by the new frame (58,59). Its frameshifting is a remarkable 74–82% efficient. It serves to divert most ribosomes to termination and allow only a greatly reduced number to continue for downstream encoded protein synthesis (a ribosome ‘sink’ function for frameshifting) (59). Such a ‘sink’ function may be ancestral in EMCV progenitors to the product of new frame translation acquiring a function. Interestingly, cardiovirus frameshifting involves a novel 3′ stimulatory element (see below) and is dependent on viral infection, suggestive of a regulatory aspect.

Instead of frameshifting being relevant to downstream polymerase synthesis, some unclassified picorna-like viruses such as *Acyrthosiphon pisum* virus (116), and *Solenopsis invicta* virus 3 (117) utilize frameshifting 3′ of their polymerase encoding sequence. They have two long ORFs with their ORF1 encoding their RNA dependent RNA polymerase 5′ of sequence encoding the capsid proteins. A −1 frameshift site at the end of the sequence for the 3′-encoded, and jelly-roll fold containing, protein permits some ribosome to decode ORF2. This frameshifting results in a proportion of the jelly-roll fold protein having an extension that protrudes from the virion capsid, and also the synthesis of other proteins.

A frameshift-derived capsid extension domain is also utilized by Orsay virus (118). This virus of *C. elegans* is from a newly identified clade of **noda-like** viruses. Betanodaviruses, which have a related capsid protein, have also been predicted to use frameshifting at the end of their capsid protein ORF, though the putative extension domain is much shorter (72).

Many **flaviviruses** also use −1 frameshifting mediated access to a short ORF overlapping an internal region of their long polyprotein-encoding ORF to generate a transframe encoded protein that is N-terminally coincident with a polyprotein cleavage product. Frameshifting evolved independently on several occasions in flavivirus evolution, though the sites within particular clades are conserved (70,119). The Japanese encephalitis serogroup of flaviviruses utilize frameshifting that has significant consequences for viral neuroinvasiveness (120–122). Many insect specific flaviviruses also utilize frameshifting in their expression (119,123,124), and some of these flaviviruses have major effects on medically important flaviviruses co-infecting the same mosquito cells. Not only does the frameshifting result in the synthesis of additional functional proteins, in some species it diverts a substantial proportion of ribosomes from synthesizing 3′ encoded replicative proteins. This permits quicker recycling of ribosomes for synthesis of further 5′ encoded structural proteins, and downregulates polymerase synthesis. However, utilization of frameshifting by certain other groups of flaviviruses including those containing Zika and yellow fever viruses is unknown.

**Influenza A virus** utilizes ribosomal frameshifting with important consequences (39). The directionality of the shift, +1, is different from that used by the viruses considered above, all of which are −1. While sites utilized for +1 frameshifting 5′ adjacent to stop codons were previously known in chromosomal gene expression, +1 frameshift sites at internal positions are notoriously difficult to spot. The previously unrecognized type of site utilized by influenza A virus has provided guides for spotting the sites of other occurrences of +1 frameshifting including that for important chronic bee paralysis virus, related viruses such as Lake Sinai virus (125) and members of the *Amalgaviridae* family (126). Influenza A virus is a single-stranded negative-sense, segmented RNA virus and the frameshifting occurs in decoding its segment 3. This segment yields a single mRNA that encodes a subunit of the viral RNA-dependent RNA polymerase. The frameshift-derived product, PA-X, has the same endonuclease domain as the polymerase subunit but lacks the C-terminal region needed for association with other subunits. Key amino acids encoded by the frameshift-derived segment that are important for the host shut-off function have already been identified (127). In some viral strains the +1 frame encoded C-terminal extension is 41 amino acids and in others it is 61 (39). There is evidence for host species specificity with increasing prevelance of the shorter form in pigs for characterized reasons (128). Potential significance derives from avian influenza viruses infecting pigs that serve as ‘mixing vessels’ for the generation of novel influenza viruses with pandemic potential. Effects of PA-X on depletion of poly(A) RNA (129), and in particular on specific host RNA polymerase II transcripts (130), have been characterized. Though strain-specific (131), for some viral strains PA-X deficient viruses display higher virulence in mice than isogenic WT viruses (39,131), in contrast to the effects of disabling several other cases of viral frameshifting. No signals for stimulating frameshifting at the shift site are evident and correspondingly the level of frameshifting is very low. It is salutory that despite this very low level of the frameshifting, the frameshift derived product significantly modulates host expression. Its loss leads to changes in the kinetics of the global host response including increases in inflammatory, apoptotic and T lymphocyte-signaling pathways (39).

Fijiviruses are plant infecting segmented dsRNA **reoviruses**. Their segment 5 has a long overlapping ORF that initiates ≥365 nts from the end of the main ∼920–940 codon ORF of that segment. Frameshifting was initially considered as ‘a distinct possibility’ for expression of the ORF (132). Following later identification of the site of +1 frameshifting in influenza A virus, an appropriately positioned similar site was pinpointed in fijiviruses (125).

Like cardiovirus frameshifting, virus infection is also required for the newly discovered second site of **arterivirus** frameshifting (22). A *trans*-acting viral protein is required for frameshift stimulation (23,133). This frameshifting is in addition to the long known programmed −1 frameshifting that occurs several kilobases 3′ of the new frameshift site (Figure 3C). The classical site, which was first identified in equine arteritis virus, is at the end of the long 5′ coding sequence, ORF1a and frameshifting at it expresses a much longer replicase precursor polyprotein (134,135). The newly discovered frameshift site mediates both −1 and −2 frameshifting (22,23). The C-terminal region derived from the new frame after −2 frameshifting is relatively short but does have an alternative transmembrane region and is targeted to a different subcellular compartment. In the economically important pig virus, porcine reproductive and respiratory syndrome virus, inactivation of the −2 frameshift product results in a 50 to 100-fold reduction in replication efficiency in cell culture (22), with the product down-regulating Swine Leukocyte Antigen class I (136). The ribosomes that shift −1 instead of −2 at the same site, immediately encounter a stop codon and terminate (23).

Well known **alphaviruses** include Sindbis, Semliki Forest, chikungunya which is now causing human joint pains and fever in parts of the world where its mosquito host was not previously present, and Ross River virus. Alphaviruses were thought to encode just 9 proteins and there was a 20-year dilemma about one of them, ‘6K’. Its migration as a doublet on gels was ascribed to different degrees of acylation. The enigma was resolved with the surprising discovery that one member of the doublet, a virion components, was a completely distinct protein TF (‘TransFrame’), derived from −1 ribosomal frameshifting (137). The C-terminal one third of TF encoded by the −1 frame is hydrophilic in contrast to the hydrophobic C-terminus of the ‘6K’ product of standard decoding. TF has a significant function as in a mouse neuropathogenesis model, the mortality of virus with mutant frameshift product was <15% compared to 95% with animal infected with WT virus (138), and another mouse study has also shown substantial effects (139). Evolutionary selection for efficient frameshifting for the synthesis of TF has resulted in a remarkable diversity of stimulatory signals (140).

The genomic RNA of **phage MS2**, and that of its close relatives R17 and f2, was used as a model mRNA for many early ground-breaking protein synthesis studies and it was also the first genome to be sequenced. It is a levivirus. Though members of this genus are not as small as the mitochondrial-infecting mitoviruses and other members of the family *Narnaviridae*, their genomes are among the smallest for RNA viruses. The replicase of both the single-stranded phages MS2 and Qβ is composed of three host translational components and one viral encoded component often termed synthetase. Synthetase is encoded by the gene closest to the 3′ end of the positive-sense genomes that acts as mRNA. The termination codon of the gene for the 66 kDa Qβ synthetase is substantially closer than its MS2 counterpart to the 3′ end from which replicase commences synthesis of the negative strand. Though there is minimal space between MS2 genes, the termination codon for its 62 kDa MS2 synthetase product is 174 nt from the 3′ end of its genome. Functional MS2 replicase likely assembles from components of the translation apparatus terminating synthesis of the synthetase and acts *in cis* on the nearby 3′ end of the RNA – unlike its Qβ counterpart it cannot be isolated in a functional state free from its RNA. Cell free protein synthesis studies revealed that −1 frameshifting yields a small proportion of MS2 synthetase of similar size as its Qβ counterpart due to termination 63–61 nts from the 3′ end of MS2 RNA (73,75). Product of this size has not been detected *in vivo* whether for lability or other reasons. It has not been determined whether the frameshifting has functional significance, but because of the short generation time and large progeny yield, the intense selection could have favored even very subtle effects.

**Figure 4.**
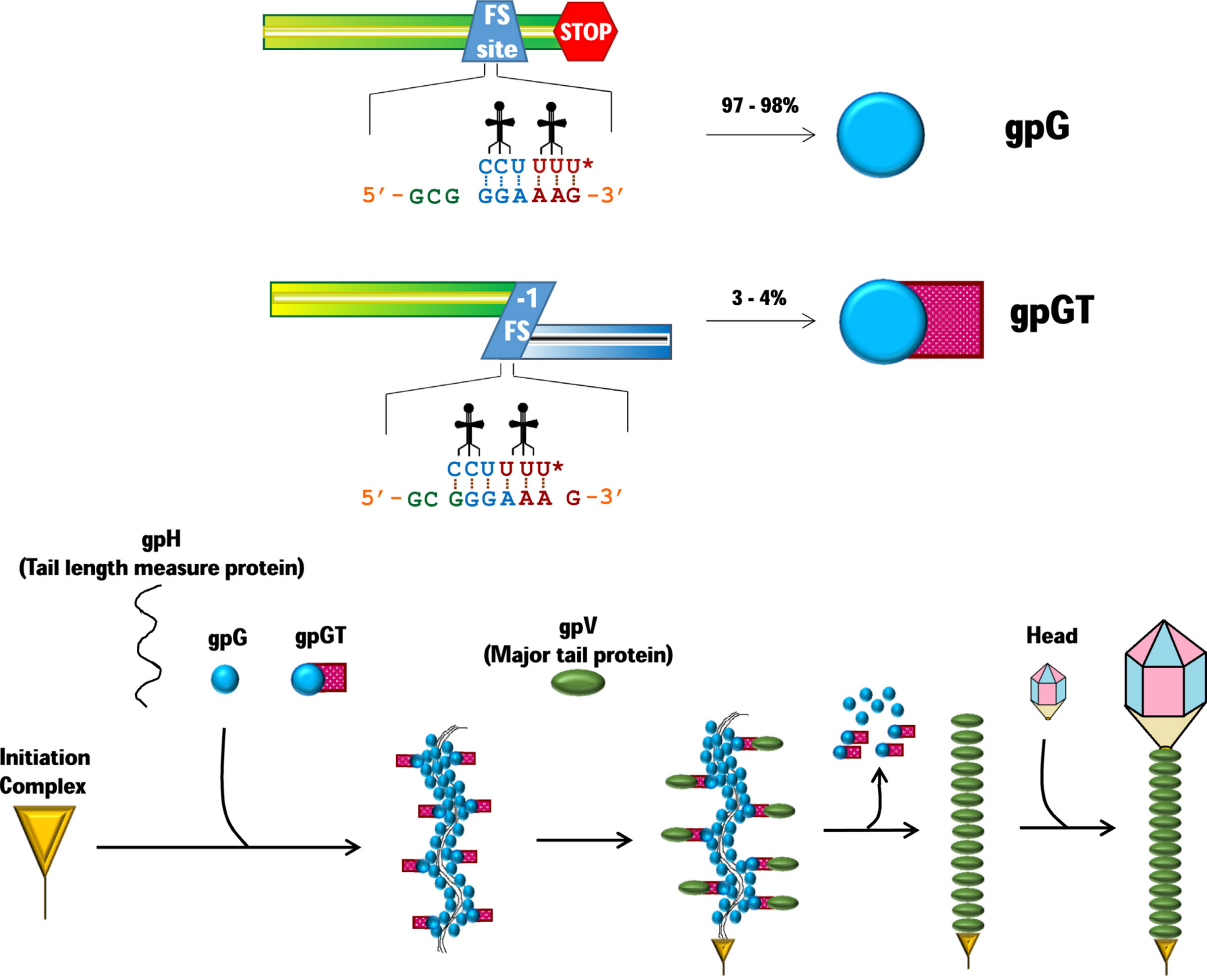
Frameshifting and phage tail assembly. Phage lambda and many other dsDNA phages gene *g* encodes gpG, a tail assembly chaperone, that binds to multiple regions of the tape measure protein gpH. Near the 3′ end of the *g* coding sequence, a small proportion of translating ribosomes shift –1 and continue to synthesize the fusion protein gpGT. The ‘G’ portion of gpGT binds to the tape measure protein gpH, while the ‘T’ region binds the major tail protein gpV, linking them and mediating the initiation of gpV on gpH. However, gpG and gpGT both dissociate from the assembly, aiding the fast polymerization of gpV on the initiator complex to form the mature tail. Finally, the head binds to the fully mature tail to form phage lambda. The tRNA^Lys^ wobble anticodon nucleotide shown as U*, represents uridine with a 5-methylaminomethyl-2-thiouridine (mnm^5^s^2^U) modification.

One of the early discoveries of viral frameshifting was the low level −1 frameshifting utilized by the double-stranded DNA tailed **phage λ** to link the synthesis of two products whose molar ratio of 30:1 is crucial for tail assembly (36,40,41). Neither product is present in the mature virions. The product of standard translation gpG, and the corresponding part of the longer and frameshift-derived gpGT product bind to the tail length measure protein, to form a complex with many molecules of gpG per single molecule of gpGT. The −1 frame encoded T domain binds to the major tail shaft subunit and may recruit it to polymerize around the tape measure protein which then determines its length (41) ∼192 copies of the major tail subunit protein are in the final structure (Figure 4). This low-level ’G-T’ frameshifting is widely conserved among other long tailed dsDNA phages, except T4, despite low sequence similarity of the respective ORFs of the different phages. However, in P2 and related phages the frameshift efficiency is 2- to 3-fold higher (141). Counterpart −1 frameshifting also appears to be utilized by the **siphoviruses** *Haloarcula vallismortis* tailed virus 1 (HVTV-1) and three *Haloarcula californiae* viruses (HCTV-1, 2 and 5) that infect halophilic archaea, though that used by the myovirus *Halorubrum sodomense* tailed virus 2 (HSTV-2) is very likely +1 frameshifting (48,49). Encoding length regulators is not unique to viruses, bacteria encode an analogous molecular ruler. One class is the Type III secretion systems – the use of transcriptional slippage in expression of several of these is described below.

The current estimate is that roughly 1.7 × 10^25^ new viruses are produced every second (142). The vast majority infect microbes, and the estimated number of phages is 10^31^ (143). Of the 5000 plus phages examined under the EM in one study, 96% are ‘tailed phages’ and one of the three main phage groups are lambda-like (another is T7-like) (144). The number of just G-T type frameshifting per second has to be a vast number.

The *Siphoviridae* temperate phage, A2, which was isolated as it destroyed *Lactobacillus casei* mediated cheese fermentation, requires two −1 frameshifting events, one being for the synthesis of an essential major head component and the other for a tail protein (145,146). Decoding in *E. coli* of its *cro* gene, which is crucial for the lysogeny: lytic decision, also features −1 frameshifting and its product is only 12 AA shorter than the product of standard decoding. Though apparently irrelevant to infection in *L. casei*, whether it is significant in other species remains undetermined (147).

Frameshifting is independently utilized to synthesize a proportion of the C-terminal immunoglobin-like domains present in diverse phages on different proteins (148,149). An occurrence of this type of frameshifting to yield an extension of the major coat protein of phage T3 results in two products from one gene in the viral capsid (150). Curiously the counterpart extension in the close relative **phage T7** (74,151,152) is not an Ig-like domain. With phage T3, and others such as *Lactobacillus* phage A2 (145,146), the Ig-like extension derives from −1 frameshifting. With ***Listeria* phages** A118 and A500 −1 frameshifting also occurs in decoding a coat protein gene, though in phage PSA it is +1 (Figure 3D). In all 3 phages additionally +1 frameshifting is utilized to derive a second product from the major tail protein gene (153,154). Apparent indifference to whether the frameshifting is +1 or −1, or even whether frameshifting is involved to yield the Ig-like extension instead of in-frame decoding, has reasonably been suggested to reflect chance. Dynamic movement of Ig-like domains via non-homologous recombination would land some in-frame and some out-of-frame with selection for function in the latter cases sometimes resulting in expression via frameshifting (155).

Kaposi's sarcoma-associated **herpesvirus** (KSHV) and Epstein-Barr virus are human DNA tumor viruses that diverged over 80 million years ago. Each expresses from a single gene a minute amount of the multifunctional products, LANA 1 and EBNA 1, respectively, important for several purposes including maintenance of viral episomes, regulation of viral latency and impairment of cell-cycle checkpoints. The products have a central repeat region that plays a role in the evasion of host immuno surveillance. While classical studies have focused on the products present in the nucleus, recently product from KSHV has been detected in the cytoplasm (156). The zero frame of the Epstein-Barr virus repeat region contains glycine and alanine, GA, codons and that of KSHV is rich in glutamine and glutamic acid, QE, codons. Although there is limited overall nucleotide sequence similarity between the two viruses, their repeat region sequence is highly similar though offset in terms of respective reading frames by 1 nt. A nested open reading frame in EBNA-1 mRNA encodes a protein capable of inhibiting antigen presentation *in cis* (157). An efficient switch, or perhaps multiple switches, to the +1 frame with respect to the genomic sequence, during expression of the EBV sequence yields a product that has ∼35% identity to the product of its KSHV zero frame counterpart (1). The product of a synthetic EBV construct designed to express what would normally be the +1 frame, inhibits antigen presenetation in *cis*, consistent with functional relevance of the frameshift derived product which is substantially present in the cytoplasm. Switching to the +1 frame, which also could be by a −2 event, occurs in the expression of the KSHV repeat sequence and it generates a highly repetitive SR-rich peptide with a distinctive subnuclear localization pattern. Whether a switch to the −1 frame, to yield a GA-rich product, also occurs in the expression of KSHV is unknown, and relevant to whether there is any significance to what is the zero frame in the coding sequence. This situation has interesting relevance to the incidental but important frameshifting in the expression of repeat sequences involved in certain human neurodegenerative degenerative disease (see below). The special cases of mutant herpes viruses using frameshifting to counteract therapeutic drugs is also considered below.

In the ancestor of **phage T4**, a homing endonuclease inserted itself into topoisomerase subunit encoding gene 39 splitting it so that the C-terminal segment became encoded by a separate gene (158), gene *60* that is ironically adjacent to the rII genes used by Crick *et al*. (159) in their famous experiment establishing sequential reading of adjacent non-overlapping codons! There is a 50 nt insert between codons 46 and 47 of gene *60* (55) that may be derived from a degenerate group I intron that provided protection against cleavage by the nuclease involved in the spread of the 5′ homing endonuclease (158). These 50 nts are non-coding and efficiently **translationally bypassed** in a process that yields a product from two disjointed and out-of-frame ORFs (55,160). The ***Streptomyces* phage** Hau3 may also use bypassing in expression of its terminase protein (161), but, if so, rather than using it to bypass a deleterious insert, it may avail of unusual features associated with its host's decoding the codon UUA (162).

The non-segmented negative-stranded RNA viruses, measles, mumps and Sendai (and other viruses in the same subfamily ***Paramyxovirinae***), and also **Ebola** (family *Filoviridae*), utilize what, after many alternatives, a key investigator is now calling programmed **transcriptional** frameshifting (PTF) (163) to yield additional product(s) that are *trans*-frame encoded with respect to genomic sequence. In bovine parainfluenza virus type 3 this is manifested in a ∼300 nt stretch of its P-gene being translated in all three frames. The P protein of these viruses is essential for activity of their RNA-dependent RNA polymerases. In Sendai and measles viruses, a slippage event at a specific site by polymerase transcribing the middle of the P gene results in 30% of the mRNA having a single ‘extra’ G (i.e. one more G in the product than the corresponding number of Cs in the template). This mRNA encodes the V protein that neutralizes host defences. For the mumps-like viruses (genus *Rubulavirus*) where the mRNA derived from standard transcription encodes the V rather than the P protein, 2 Gs are inserted at high frequency with the resultant mRNAs encoding the P protein. The pattern of G inserts for each virus reflects their respective ORF possibilities, and they have a mechanism to ensure that RNA with G inserts is not packaged into virions. In contrast, *Ebola virus* slippage occurs during transcription of its non-structural glycoprotein gene to yield transcripts without inserts and with one or two additional As in the ratio 70, 25 and 5% that encode soluble glycoprotein, transmembrane glycoprotein and small soluble glycoprotein, respectively (164–166). A knockout of the slippage site in a recombinant Zaire ebolavirus significantly increased cytopathogenicity indicating a role for slippage products in reducing early cytotoxicity (165,167).

**Figure 5.**
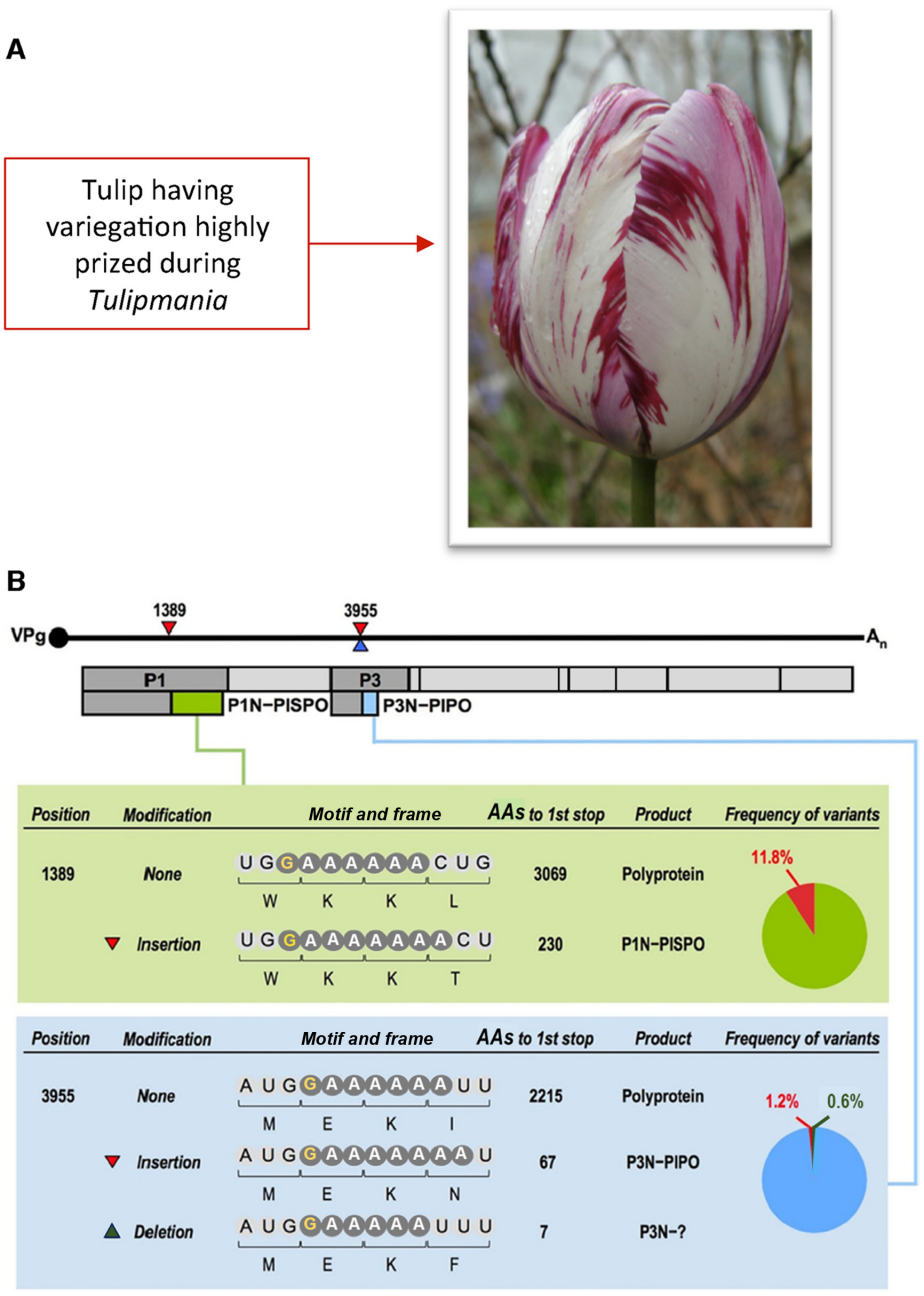
Potyvirus transcriptional frameshifting. This permits expression of the overlapping ORF pipo whose product (light blue) is part of a protein, P3N-PIPO involved in viral movement between cells, and therefore essential to virus viability. Potyviruses are important as major pathogens of agricultural crops and for their effects on other plants. The effect of tulip mosaic potyvirus on flower pattern caused one bulb at the height of tulip-mania in the 1625 to cost ‘*Four tons of wheat; eight tons of rye; four fat oxen; eight fat pigs; twelve fat sheep; two hogsheads of wine; four barrels of beer; two barrels of butter; one thousand pounds of cheese; one bed, with accessories; one full-dress suit; and one silver goblet*.’ A second instance of transcriptional frameshifting in a small subset of potyviruses yields the product P1N-PISPO (PISPO in light green) [RNA Polymerase Slippage as a Mechanism for the Production of Frameshift Gene Products in Plant Viruses of the Potyviridae Family. Rodamilans, B., Valli, A., Mingot, A., San León, D., Baulcombe, D, López-Moya, J.J., and García, J.A. *J. Virol*. (2015) 89(13) 6965-6967, doi:10.1128/JVI.00337-15, reproduced with permission from American Society for Microbiology.].

**Potyviruses** may cause more than 30% of all plant losses due to viruses and cause immense monetary loss and wasted fuel in intensive agriculture, a figure of $20 billion per annum has been cited (168). Regardless of the monetary figures, they cause severe hardship and starvation for many on subsistence agriculture. In contrast, a potyvirus caused the highly prized pattern on tulips that triggered the first economic boom bust ‘mania’ in Europe (in the 1600s), and tests for potyviruses in orchids are lucrative (Figure 5). Until recently the small single-stranded RNA genome was though to have a single ORF that encodes a polyprotein which is cleaved to yield functional proteins. However, there is a short overlapping ORF, *pipo*, that is decoded as the C-terminal end of a fusion protein P3N-PIPO (169) which mediates virus movement in plants (168,170) and is relevant to overcoming host resistance (171) and jumps in plant host range (172). Though it was initially suspected that PIPO coding sequence was accessed by ribosomal frameshifting, it is instead specific viral polymerase slippage, with insertion of a single additional base relative to the template, followed by standard translation, that yields the additional product (42–44). Irrespective of the outcome of future work to determine whether the slippage events occur during synthesis of the minus-strand replicative intermediate or the plus-strand progeny genome, the mRNA with the single extra base behaves as a novel sort of sub-genomic (actually, super-genomic) RNA. The requirement for synthesis of a complete polyprotein to confer *cis*-replication competence (173) has been suggested to mean that the RNA from which PIPO is translated, is not amplified (42). With turnip mosaic virus, the amount of deletional or +2 slippage, was very low so the amount of expression from the +1 frame was very small. However, with clover yellow vein virus, a product with its C-terminal 5AA encoded from the +1 frame, and derived from transcriptional slippage at the same site, has been detected, and shown, like P3N-PIPO, to function in cell-to-cell movement (174) (Figure 5B). Four related potyviruses infecting sweet potato utilize an additional slippage site at a different location to permit access to a second overlapping ORF and synthesis of a ‘transframe’ product that uniquely inhibits short-distance movement of an RNA silencing signal (175,176).

The sequence of the single-stranded RNA genome of **hepatitis C virus** that encodes a large polyprotein precursor has within it an alternative reading frame that specifies a product that has been linked to liver cancer, although it remains controversial as to whether the product is functionally relevant to virus growth or simply a manifestation of translational noise. A role for ribosomal frameshifting (177) or transcriptional slippage (178) in expression of the alternative product has been discussed. However, much evidence now favors internal initiation (179), though more than one mechanism may contribute to synthesis of the ‘extra’ product.

Though **dicistroviruses** are not known to utilize frameshifting, the expression of at least some of them utilizes a mechanism with similarities to that utilized in one or more cases of frameshifting. This is described in the section ‘Stimulators 5′ of ribosomal frameshift sites that act at the mRNA level’. As implied by the name for these viruses that infect bees, ants, flies, aphids, silk worms and some other arthropods, one of their characteristic features was thought to be the presence of just two ORFs separated by an intergenic IRES in their monopartite genomic RNA. However, at least the acute bee paralysis clade of dicistroviruses have an additional, and important, ORF, *orfX*, that overlaps in the +1 frame the ORF long known to be expressed from the intergenic IRES (180,181). This IRES, which has been useful as a reagent for ‘all-RNA’, i.e. factorless, and methionine-independent initiation, can mediate initiation in two alternative frames. Paradoxically, the genomes of this subgroup of dicistroviruses can now be seen to be tricistronic.

### Virus summary

Eukaryote-infecting viruses that utilize frameshifting in their expression are listed in Table 1. At present it appears that decoding of a substantial majority of all cellular and viral genomes likely productively utilizes frameshifting (inclusion of bacterial viruses is relevant). Viruses, especially RNA viruses, and other mobile elements, have comparatively small genomes, and the prelevance of frameshift utilization appears more striking than in much larger cellular genomes. As compact genomes, expecially those whose expression does not involve splicing, richly use translational versatility and controls, it is not surprising that frameshift utilization is no exception. Utilization of at least one frameshifting event in RNA virus decoding is almost more the rule than the exception. With the ongoing application of deep sequencing to viruses of terrestrial invertebrates, marine species of all types and to bacteria plus archaea in numerous isolated deep sub-surface niches, knowledge of instances of viral frameshifting seems certain to expand (with at least the bacterial and achaeal genomes providing prophage/integrated viral genome information).

**Table 1B.** Known and predicted occurrences of polymerase slippage in virus genomes. In column S, ‘+’ and ‘-’ indicate positive-sense single-stranded RNA and negative-sense single-stranded RNA viruses, respectively

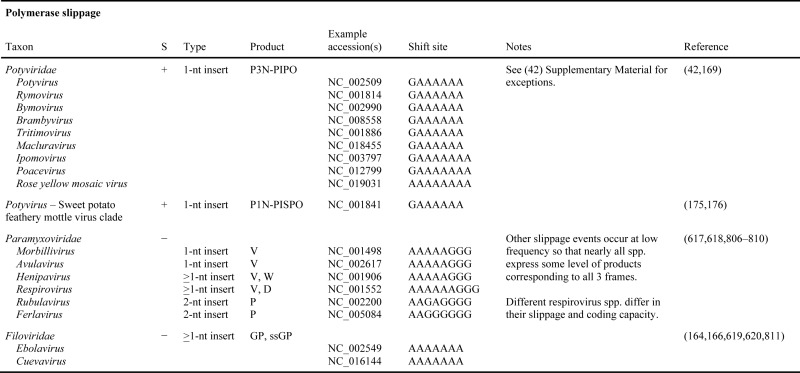							

### Mobile chromosomal genes: from IS elements to retroelements

**Figure 6.**
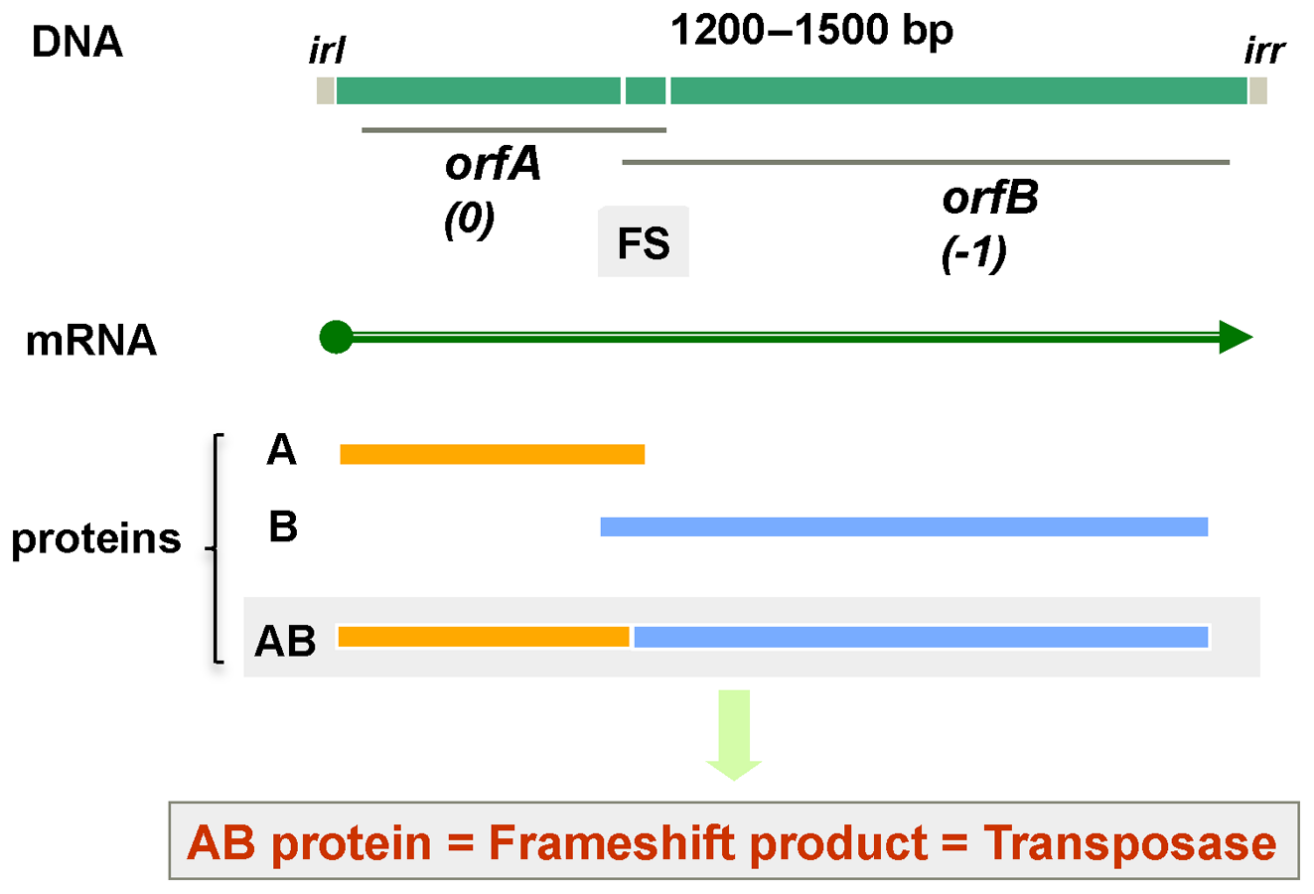
Bacterial Insertion Sequence (IS) with two partially overlapping ORFs. Many IS elements have this type of gene organization. Utilization of ribosomal frameshifting to allow a proportion of ribosomes translating *orfA* to access *orfB* has been studied in depth in a variety of IS elements, but transcriptional slippage is now known to be used as an alternative by a substantial number of IS elements.

Bacterial **IS elements** are small, 800–2700 bp, transposable elements. Sometimes they are present in only one or a few copies per bacterium, but up to more than a thousand in some cyanobacteria (182). The over 4000 known different IS elements have been classified into several families (183). While use of a frameshift event occurs in the decoding of the IS1 (184), IS3 (185,186), IS5 and IS630 families, it is very common in the IS3 family, which comprises 27% of the known IS elements (187). Frameshifting serves to fuse the product of an upstream ORF, which generally encodes a DNA-binding protein that can on its own, act as a regulator, with a catalytic domain encoded by a partially overlapping downstream ORF that is often in the −1 frame (Figure 6). The fusion has the transposase activity. Where tested, the level of frameshifting was found to determine the level of transposition (188,189). As a low level of transposase is optimal to minimize insertion-mediated host gene disruption, the frameshifting level is generally low. This mostly affects transposition frequency of the same IS element rather than copies of the same element located elsewhere on the chromosome, since transposase has a strong *cis* preference. This is related to the frameshifting itself since an associated pause signal influences *cis* preference presumably by facilitating sequential folding and cotranslational binding of the transposase (190,191).

Because of the enormous number of IS elements, and the prevalence of their utilization of frameshifting for transposase expression, they constitute one of the richest sources of frameshift cassettes. Most of the work on IS element frameshifting, including all the early studies focused on IS elements where the frameshifting is at the translational level. In particular, detailed studies by Olivier Fayet and colleagues (192–195), has provided insights into a variety of −1 ribosomal frameshifting features not revealed by high level frameshifting, including frameshifting without P-site re-pairing following dissociation, dual use of stimulatory elements and detailed knowledge of shift site usage. IS elements that use +1 ribosomal frameshifting have not been identified. However, several of the more recent studies have focused on IS elements where transcriptional realignment yields the transposase product that is encoded *trans*-frame with respect to the DNA sequence. In several of these the shift is +1 (99,196–198).

Even with the original concept of IS elements as being short and only encoding enzymes necessary for their own transposition, such ‘solo’ elements have substantial effects on chromosomal gene arrangement and expression. It has long been known that translocation of diverse chromosomal segments with different genes by flanking IS elements broadened IS significance. More recently it has been found that passenger genes, e.g. encoding transcription regulators or antibiotic resistance, can be within a ‘transporter’ IS element, and in other ways also relevant distinctions are becoming imprecise, e.g. among IS elements of *Xanthomonas* phytopathogens (199). The distinction between certain IS and the much larger ICE type elements is also becoming more ‘fuzzy’ in part because of the type of transposase used by several of the latter (183).

**ICE** (Bacterial Integrative and Conjugative Elements) are the most abundant conjugative DNA elements in bacteria. Naturally occurring non-symbiotic mesorhizobia that receive one of the well-studied elements, 502 kb ICE-*Ml*SymR7A, via conjugation can communicate interactively with the pasture legume commonly known as bird's-foot trefoil or deervetch (*Lotus corniculatus*), leading to the formation of bacteroids that fix atmospheric nitrogen into ammonia (200). Countervailing the potential population benefit of new endosymbiont formation is selection against disadvantageous occurrence to the original host of the element excision. Excision results in circularization and rolling circle replication. The element can be maintained in the multi-copy extrachromosomal state at energetic cost to the host, and in this state it has greater transfer propensity. For the vast majority of cells carrying the ICE element, this cost does not arise since in WT laboratory *M. loti* strain R7A, the excised state is only observed in 0.06% of log-phase and 6% of stationary phase cells with only ∼3 in 10 million cells acting as transfer donor. A quorum sensing system keeps the process at a very low level but when derepressed excision can occur in 40–100% of cells and the transfer rate can increase 1000-fold. The ICE element encodes components responsible for a complex system of activators and repressors for quorum sensing and also components of the excision system responsive to it (201). Crucial for excision is a product expressed from genes *msi172* and *msi171*. Plus one ribosomal frameshifting near the 3′ end of the *msi172* coding sequence causes ribosomes to enter the *msi171* ORF and synthesize Frameshifted excision activator, FseA, a master transcriptional activator of the excision system. Perhaps to modulate leaky expression, FseA itself is subject to binding and inhibition by a quorum-sensing antiactivator (202). This antiactivator's own expression is bimodal so that individual cells are either ‘on’ of ‘off’ for propensity for ICE excision. This has been proposed to be part of a ‘bet-hedging’ strategy in which only a small proportion of cells in a population are sensitive to induction of excision, with potentially deleterious consequences for those cells but potentially beneficial cell population benefits. In this scenario frameshift utilization is part of the scheme for dampening biological noise present in a quorum-sensing auroinduction circuit and ensuring that excision is not spuriously induced. Ramsay, Ronson and colleagues who discovered the frameshifting involved, anticipate that nutrient availability likely contributes to the proportion of cells that enter the ‘on’ state, with frameshift efficiency being a sensor (201,202). Identification of the potential for frameshifting in similar genes in other elements suggests more widespread utilization (202) but the extent of this awaits investigation.

Transposable elements are also important in eukaryotic genome dynamics and evolution with a substantial proportion of the human genome being composed of retroposons, retrotransposons and their remnants. Though by definition retroposons do not encode reverse transcriptase, a small proportion of them and of derivative sequences, do have some retroviral-like features. While they are significant, other retroposons are also important, e.g. some alter the genetic landscape of the human brain (203,204), another has influenced maize domestication (205) and many are important in genome dynamics and evolution.

The studies of Phil Farabaugh's group on the expression of *Transposons of yeast*, **Ty elements**, is a classic in the study of +1 frameshifting and is pertinent for other +1 *S. cerevisiae* frameshifting. In contrast to IS element usage of −1 frameshifting, *S. cerevisiae* Ty1, 2, 3 and 4 retroelements utilize +1 frameshifting near the 3′ end of their *gag*-homologue gene to synthesize the GagPol polyprotein from which Pol and ultimately reverse transcriptase, is derived. Ty1 and Ty3 are present in up to 30 copies and 1–5 copies per cell, respectively, and their frameshifting is distinctive. Ty1 frameshifting was discovered at an early stage (206–208). It utilizes a highly shift-prone site; 40% frameshifting was recorded with reporter constructs (209). Yet its ratio of GagPol to Gag is only 3% (210), much closer to that of other mobile elements. After the cap at the 5′ end of the mRNA there is a pseudoknot that includes the start of the coding sequence but it serves to only modestly reduce the level of *gag* translation (211). Though it does not yield accurate quantitation, the results from ribosome profiling for both Ty1 and another gene, ABP140, with the same shift site in their native chromosomal states clearly point toward the efficiency deduced from the reporter construct work ((212); A. Michel and PVB, unpublished). The discrepancy for Ty1 between 40% frameshifting and the much lower ratio of GagPol to Gag is currently ascribed to either a translational elongation effect in decoding the downstream *pol* gene, or reduced stability of GagPol compared to Gag (213). The frameshifting utilized by another Ty element, Ty3 is mechanistically quite distinct (214,215). Its frameshifting is not a constitutive process but is regulated over a 10-fold response window depending on carbon source. The glucose-signaling pathway involved has been investigated in depth (216).

A bioinformatic analysis of higher eukaryotic LTR retrotransposons in 2003 showed that 28 of 51 animal retroelements surveyed had their *pol* in the −1 frame relative to *gag* and many had appropriately positioned classical heptanucleotide tandem shift sites. Most were in *D. melanogaster* and none were in *C. elegans*. Only 2 of the 51 had *pol* in the +1 frame whereas in fungi the proportion using +1 compared to −1 was less skewed. At that time just two different types of plant elements were found to have *gag* and *pol* in separate frames and in both *pol* was in the +1 frame. One has a *gag pol* overlap of 310 nts. The other type is a set of sirevirus retrotransposons that comprise two sub-sets, one of which has a simple *gag pol* overlap. However, the second sub-set has a conserved UAG in the *pol* frame 10 nts 3′ of the *gag* terminator. Both sub-sets from both monocots, and dicots have a conserved sequence motif of 27 nts that can form an 11-bp stem containing covariant nucleotides and capped by a 4-nt loop (217). Puzzles awaiting resolution abound.

### Retrotransposons with acquired organismal functions

Retrotransposons play major roles in the expression of ‘host’ genes (218) and in addition some derivatives have acquired a protein coding function important for their host (neofunctionalized) together with immobility and nevertheless utilize frameshifting in their expression. Two major gene families are derived from Ty3/Gypsy long terminal repeat (LTR) retrotransposons, **PEG10** (Human Paternal Expression Gene 10, also known as EDR, embryonal carcinoma differentiation regulated) being in one of the families and PNMA related genes in the other. PEG10 is not expressed from the maternally derived gene. Its expression was shown to utilize frameshifting (219) and subsequent work identified the correct pseudoknot frameshift stimulator (220). The frameshifting occurs at a very high level, 60%, in the later stages of developing placenta and is likely important (221). Knock-out mutants of PEG10 are lethal at an early embryonic stage and PEG10 products have numerous effects including inhibiting apoptosis and mediating cell proliferation (222,223). Expression in adult tissues has been more difficult to demonstrate but it may also be expressed in the adrenal glands of mammals (224).

The protein products of the paraneoplastic mammalian antigen-like genes **PNMA3** and **PNMA5** (Ma3 and Ma5) genes are targets of an autoimmune response triggered by the ectopic expression of antigens in tumor cells (225). The effects of the indirect autoimmune response are frequently detectable before the tumor or its direct consequences. In contrast to Ma3 the evolutionary distance between Ma5 orthologs from mouse and human is far longer. Ma5 is not expressed in mouse brains and its expression in primate neocortex is highly restricted to a specific layer of an association area unlike Ma3 (226). Ma3 expression involves ca. 20% efficient −1 frameshifting at a classical heptanucletide shift site stimulated by a classical H-type pseudoknot, and though not analyzed in detail, Ma5 appears similar (227). The product of the sequence accessed by frameshifting is not similar to any known protein (neither Ma3 nor Ma5 encode any Pol domain). PNMA evolution in marsupials has yielded significantly different PNMA genes (228).

**ZCCHC5** (also known as Mar3, Mammalian Retrotransposon-derived 3) lost its ability to retrotranspose at least 100 million years ago and contains a partial *pol*-like sequence. Like other similar genes, it has evolved to encode a protein beneficial for host fitness and continues to evolve under purifying selection. Many such neogenes are under epigenetic regulation, and maybe derivatives of their retrotransposon ancestor selected for cellular defense against that ancestor. ZCCHC5 has been predicted to utilize frameshifting in its expression (229) and this has been experimentally demonstrated (N.M. Wills and JFA unpublished).

### Retrotransposon derivatives and Telomerase

*Drosophila* lacks telomerase; instead for at least 40 million years, three telomere-specific non-LTR retrotransposons have maintained chromosome ends. One of the three, HeT-A encodes only a Gag-like protein and not its own reverse transcriptase. Nevertheless, this important *gag* gene requires a frameshifting event for its expression (230,231). In another, TART(-B1), the *gag* termination codon is followed 3′ by 37-nt before a +1 frame start codon which is presumed to initiate translation of the polyprotein that includes the reverse transcriptase (232). As there is a stop codon in the +1 frame in the 37-nt ‘spacer’, if a GagPol product is synthesized via a tRNA realignment mechanism, it would likely involve bypassing rather than +1 frameshifting, but how the important polyprotein is expressed is unknown. Bypassing was considered as a possibility for synthesis of the counterpart product of mammalian LINE elements. Though only a tiny proportion of those in the human genome are active autonomously, collectively they are important in several ways. They have just three ORFs (233), with a small number of nucleotides separating the 3′ end of ORF1 from the start of ORF2 that encodes an endonuclease and reverse transcriptase activity. However, rather than bypassing being involved in ORF2 expression, some unknown and novel type of initiation, independent of the AUG present, is considered more likely for the curiously stochastic expression involved (234,235). In contrast to *Drosophila*, as described in the section on special characteristics of ciliates and *S. cerevisiae*, the ciliated protozoan *Euplotes* has telomeres with frameshifting being required in the synthesis of the telomerase protein La and the reverse transcriptase (TERT) that is the homolog of *S. cerevisiae* Est2p. In another ciliate, *Oxytricha*, frameshifting would be required for expression of transposon-derived telomere-bearing elements, whose transposase has been ‘domesticated/neofunctionalized’ for the genome rearrangement involved in generating socatic macronucleus from germline micronucleus. These elements occupy 13.3% of the micronucleus DNA, and despite the presence of pseudogenes, purifying selection suggests many are expressed (236).

#### Est3

Plus one frameshifting is required for synthesis of the *S. cerevisiae* telomerase regulatory subunit Est3 (Ever shorter telomeres 3) (237). A three-protein preassembly complex is present in most of the cell cycle and late in the cycle, after completion of DNA replication, Est3 binds to form active telomerase, which is quickly disassembled by a different pathway. Est3-mediated limitation of the time of formation and quantity of telomerase to a low level may be important for avoidance of telomerase acting at double-strand breaks where it could prevent repair that would restore genome integrity (238). The +1 frameshifting occurs at a shift site similar to that of Ty1 just before a UGA terminator at codon 94. *trans*-frame encoded Est3 is 181 amino acids. The surface of Est3 that interacts with the telomerase RNP pre-assembly complex, the ‘TEL’ patch is similar to that of mammalian protein TPP1 (239,240), and is encoded downstream of the shift site (240). Under the conditions tested, the frameshift efficiency is high in part due to an 8-fold stimulation by a 3′ modular 27-nt stimulatory sequence (237,241). Even though a mutation putting the coding sequence into a single frame had no discernible effect on growth in lab media (237), function is evident because of conservation, including of the 3′ stimulator, in diverse budding yeast for 150 million years (242).

### Mitochondria and endosymbionts: translational flexibility permitting genomic latitude/‘sloppiness’

Like viruses, mitochondria and many endosymbionts have comparatively small genomes. Though commonly they don't encode a large proportion of their own translation components, they have their own compartment specialized translation system. Vertical transmission with avoidance of gene mixing likely contributes to indels in certain sequence contexts being retained if their deleterious effects are initially even partly compensated for by translational frameshifting. Since the translation system only decodes a modest number of sequences, selection presumably has enhanced possibilities to make the ‘corrective frameshifting’ very efficient.

Some long-term bacterial endosymbionts of insects have their genomes reduced to the range of 160–790 kb and an A T-content >70% (243). In frameshifted cell wall genes of an endosymbiont associated with aphids, and a histidine biosynthetic gene of an endosymbiont associated with ants, frequent transcription slippage/realignment at long poly(A) runs yields 12–50% of transcripts with ‘corrected’ reading frames (244). The pathogenic kinetopast protists *Trypanosoma* and *Leishmania* have long been known to have mitochondrial uridine insertion/deletion type editing. But it also occurs in the mitochondria of endosymbiont kinetoplast *Perkinsela* where only six protein-coding genes have been identified despite the large size of the *Perkinsela* mitochondrial genome. Given the number of post-editing ‘problem’ mRNAs (245), potential translational ‘correction’ seems also likely. Ribosomal frameshifting can also compensate for the effects of otherwise inactivating single-nucleotide insertions at various places in the encoding DNA, and examples occur in the mitochondria of several free-living species. Single nucleotide inserts occur in a small number (currently 6 known) of essential genes in the mitochondria of species from 4 animal phyla and 1 protist. Compensatory +1 ribosomal frameshifting apparently yields an adequate amount of functional product (246). An insert occurs in the NADH dehydrogenase subunit 3 gene of the majority of birds (247,248). The homologous gene in some turtles has the same insert and the insert likely originated in a common ancestor of birds and turtles more than 200 million years ago (246). In some other turtles the insert is elsewhere (246,248). An insert also occurs in the cytochrome b genes of at least one oyster species (249) and at any of several sites in this gene in several ant species (250). However, among the currently known instances, natural mitochondrial single-nucleotide frameshifting is most prevalent in glass sponges (251,252). These siliceous spicule-containing Hexactinellid sponges have exceptionally low metabolism, live in a low oxygen environment and their mitochondrial coding sequences show evidence of relaxed selection pressure (252). The coding sequence of mitochondrial genes of several calcium carbonate spicule-containing Calcaronean sponges is largely unrecognizable and their transcripts have single or double U insertions in pre-existing short poly(U) tracts (nearly all their genes on individual and likely linear chromosomes) (253). How the inserts occur and whether extensive compensatory (‘corrective’) frameshifting is involved in the synthesis of their protein products is unknown.

From studies of numerous inserts and their loss in certain lineages, it is estimated that the average time for deletion of the insert that causes out-of-frame decoding is 114 million years, and about 5% of removal times will be >350 million years. Persistence often over hundreds of millions of years, and loss at various times has led to a mixed pattern of frameshift conservation in this ancient lineage (252). However, a cytochrome c oxidase subunit 1 coding sequence of a genus of early diverging dinoflagellate-like Protists that cause disease of marine molluscs including farmed oysters, has either 10 frameshifts or else tRNAs for decoding the quadruplet AGGU as glycine and CCCU as proline (64,254).

Inserts of not just single nucleotides but blocks of nucleotides occur at 81 positions in the mitochondrial genome of the yeast *Magnusiomyces capitatus* and involve frame disruptions of 12 of the 15 protein coding genes (65). Compensatory frame restorative translation permits mitochondrial function (65), with apparently similar inactivation avoidance in *Saprochaete clavata* (56). This raises the issue of in-frame bypassing in at least certain other mitochondria with provocative sequence features that would result in a proportion of the product lacking some internal amino acids. Such in-frame translational bypassing was experimentally demonstrated with synthetic constructs in *E. coli*, and though in-frame stop hopping could be the explanation for certain cases of what is currently thought to be natural stop codon readthrough, no occurences are known.

In contrast to mitochondrial frameshifting to render nucleotide inserts innocuous, −1 frameshifting has been reported to be required in the decoding of two human mitochondrial genes for which the codon 3′ adjacent to the last sense codon in these genes is AGA or AGG. Its proposed function is to bring a stop codon, UAG, into frame (255). But this has been debated (256–259).

## FREE LIVING ORGANISM CHROMOSOMAL GENES LACKING MOBILE PREDECESSORS OR TELOMERASE CONNECTIONS

### Antizyme: frameshifting as a sensor and effector for polyamine regulation from yeasts to mammals

**Figure 7.**
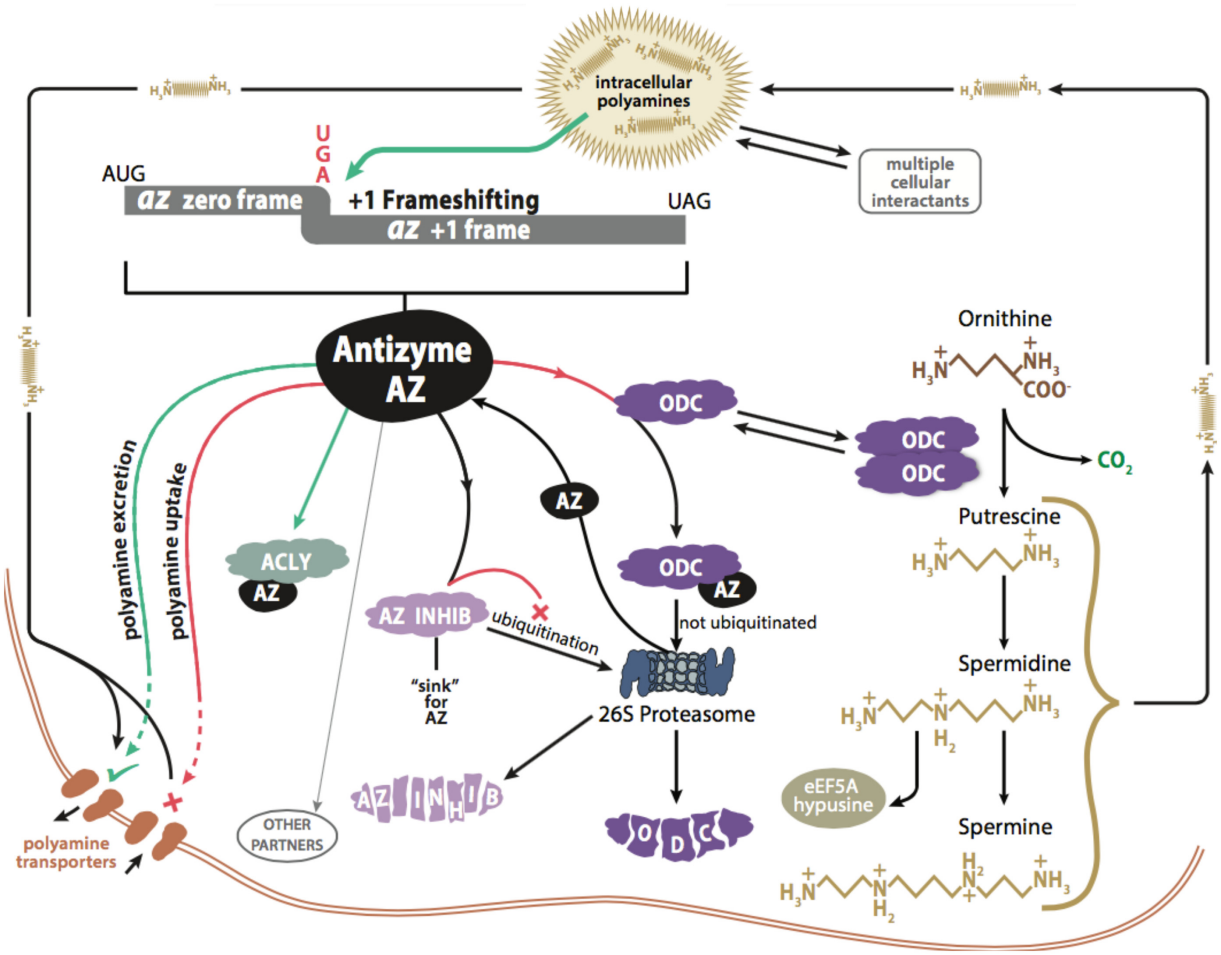
Schematic representation of synthesis of mammalian antizyme-1 (AZ) protein and the interactions between it, ornithine decarboxylase (ODC), antizyme inhibitor (AZ Inhib), ATP citrate lyase (ACYL) and polyamine transporters. Increasing polyamines enhances the ribosomal frameshifting required for synthesis of antizyme, which then acts to inhibit synthesis and uptake of polyamines. Antizyme binding to an ODC monomer prevents the ODC dimer formation required for catalyzing synthesis of putrescine from which the polyamines spermidine and spermine are derived.

The positively charged low molecular weight diamines and polyamines, putrescine, spermidine and spermine, occur in all cells. They play crucial roles in ion channels and many biochemical processes including transcription and translation (Figure 7). Not surprisingly, they have disease relevance (260). The over 80 000 publications listed in Pubmed are a reflection of the level of interest they have attracted. Though elevated levels occur in cancer cells, their levels are normally tightly controlled. A key regulator of intracellular polyamine levels is the protein antizyme whose existence was initially postulated as an ‘anti-enzyme’ inhibitor of ornithine decarboxylase that catalyzes the synthesis of putrescine from which spermidine and ultimately spermine are derived (261). Despite considerable skepticism about the reality of its existence, and practical difficulties, a mammalian antizyme gene was cloned. Two partially overlapping reading frames were identified, and the protein sequence encoded by the frame junction region was determined (262,263). Not only is +1 frameshifting required for antizyme synthesis, the frameshifting is a very significant component of the regulatory repertoire (264–268). There are three antizymes in mammals (see below), more in the fish, *D. rerio* (269–271), and one in each species from *Drosophila* to *C. elegans* to *S. cerevisiae* yeast (272–274). Frameshifting does not occur in the decoding of the antizyme gene from the ciliate *Tetrahymena* (270) where UGA is reassigned from being a stop codon. An antizyme gene is not present in extant plants. Binding of mammalian antizyme 1, but not antizymes 2 or 3, to an ornithine decarboxylase monomer causes a conformational change that, without the involvement of ubiquitination, targets the ornithine decarboxylase monomer for degradation by the 26S proteasome via its own C-terminal segment (275,276), and at least in *S. cerevisiae* the counterpart targeting is promoted by polyamine binding to antizyme (277). Polyamine binding to antizyme also influences ubiquitin-dependent degradation of antizyme itself (277), and antizyme has similarities to a key polyamine catabolic enzyme, spermidine/spermine *N*‐acetyltransferase, that directly binds polyamines.

In addition to the competitive inhibition that all antizymes exert on formation of active ornithine decarboxylase dimers, as shown by many studies (260), antizyme 1 also negatively controls polyamine extracellular uptake via receptor interaction (278,279), and promotes polyamine secretion (280). In Filarial roundworm parasites there is no functional ornithine decarboxylase but still an antizyme whose function, at least in part, is likely for regulating polyamine uptake (281). Antizyme 1 is in turn subject to inactivation by antizyme inhibitory proteins, e.g. (282) and (283–285) whose synthesis is governed via uORFs by polyamines (286).

Mammalian antizyme 1 binds to ATP citrate lyase that catalyzes the synthesis of acetyl-CoA which is involved in lipid anabolism and also in acetylation of cellular proteins. Instead of accelerating ATP citrate lyase degradation, antizyme performs an activating role. Cellular spermidine and spermine induce spermidine/spermine-*N*^1^- acetyltransferase which acetylates polyamines for catabolism or secretion. A regulatory circuit involving its substrate acetyl-CoA in downregulating spermidine and spermine through acetylation has been proposed. An influence on acetyl-CoA has wider implications, including for histone acetylation, and initial studies on cholesterol have been performed (287). Antizyme has also been reported to bind to Smad1, cyclin D1 and Aurora-A (288–290), though there has been some controversy about this. A study of the binding sites of antizyme and cyclin D1 also revealed that binding affinity was 4-fold lower than for antizyme and ornithine decarboxylase (291) for which high resolution structural information is now available (276).

Antizyme 1 mRNA is present in high abundance, and its expression is regulated to yield low levels of protein which is unstable. There are two start codons with a mitochondrial localization signal encoded between the first and favored second start 34 codons downstream (292). The frameshifting required for synthesis of functional antizyme occurs at the last codon of ORF1, and is +1.

The frameshifting increases strongly with elevated polyamine levels, and the resultant increased synthesis of antizyme serves to decrease both the synthesis and cellular import of polyamines. Thus, frameshifting is both a sensor of polyamine levels and effector of an autoregulatory circuit (265,293). Known features relevant to this are considered in the sections on mRNA elements 5′ of the shift site, mRNA structural stimulators 3′ of the shift site and especially nascent peptide modulators where unappreciated features are considered.

Before the second and more conserved antizyme gene, antizyme 2 (294), was identified as encoding an antizyme, it was recognized as one of a very small number of genes upregulated by a seizure-inducing substrate in neuronal cells (295). Antizyme 2 protein is phosphorylated and is localized more in the nucleus than is antizyme 1 (296). While antizyme 1 has nuclear import and export signals, part of which is encoded by its ORF1, it is the C-terminal region of antizyme 2 that is involved in its nuclear translocation perhaps by association with another protein that does have a nuclear localization signal (296). Forced expression of at least antizyme 1 has strong tumor suppressive properties (297).

There is also a third mammalian antizyme, antizyme 3, and it is restricted to developing male germ cells (298,299). In contrast antizymes 1 and 2 are expressed in nearly all other cells where the requirement for polyamines may be lower. Functioning antizyme 3 is required for the rigid connection of sperm heads to tails and so for male fertility (300,301). A substantial proportion of the translation initiation on antizyme 3 mRNA is at an in-frame CUG codon further 5′ with respect to the frameshift site than the AUG initiators for antizymes 1, 2 or 3, and so the product is N-terminally extended (301). Frequent termination at the stop codon 3′ adjacent to the frameshift sites can result in synthesis of the ORF1 product, p12, that has a distinct separate activity. p12 interacts with myosin phosphatase targeting subunit 3 (MYPT3), a regulator of protein phosphatase 1β, and affects phosphatase activity (301). Expression of full-length antizyme 3 is very low, and at least in heterologous tissue culture cells the frameshifting efficiency is also very low (266). In marked contrast to antizymes 1 and 2, at what is expected to be standard expression levels, antizyme 3 has a stabilizing effect on it, probably by antagonizing antizyme inhibitor. Instead of targeting ornithine decarboxylase for degradation, antizyme 3 may serve to reversibly ‘store’ ornithine decarboxylase monomers (302). This mechanism would allow a much faster restoration of ornithine decarboxylase activity than any mechanism mediated by antizymes 1 or 2, as they require *de novo* synthesis of the protein (302).

Polyamine biosynthesis occurs cyclically during the cell cycle with antizyme and shows inverse activity (303). Furthermore, polyamines regulate the circadian rhythms by modulating the interaction between core clock components (304). The tropical fresh water fish *Danio rerio*, and others of the minnow family, have a unique antizyme that is expressed in their retina and brain but absent in many other fish (271). Sequence inspection suggests that its frameshift recoding signals are weaker than the other antizyme mRNAs present in fish (or mammals other than antizyme 3) (271). As the levels of polyamines to which it is responsive are likely different from that of other antizymes, it will be interesting to see if its expression is circadian clock linked with effects on fish day night behavior.

Agmatine has long been known to affect antizyme frameshifting (305) and antizyme influences uptake of the nitric oxide synthase inhibitor, agmatine, that has neurological significance (306). The influence of antizymes, both by direct binding and indirectly, on other pathways and the potential role of factors other than polyamines on antizyme frameshifting, is the subject of several reports and of active investigation (291,307,308). The potential for polyamine levels to have specific effects on other cases of frameshifting also needs to be kept in mind especially as a certain combination of the levels of putrescine and spermidine was shown to influence the *S. cerevisiae* mobile element Ty1 frameshifting and so the rate of its retrotransposition (309). Because of the up-regulation of polyamine synthesis in important disease, much work is being devoted to identifying inhibitors. From a frameshifting perspective, the distinction between the very different situations with *S. cerevisiae* antizyme and mammalian antizyme 3 and the contrasts between each of them and the other types of antizyme frameshifting, is intriguing even with our current limited knowledge.

### Magnesium chelatase: also regulatory?

Genes for magnesium chelatase from bacterial genera such as *Pseudomonas* and gold mineralizing *Delftia* and the archaea *Methanocaldococcus* and *Methanococcus* are organized in overlapping ORFs with an A_AAA_AAR sequence evolving under strong purifying selection suggesting that it is used as a shift site for evolutionarily conserved frameshifting (198). Heterologous expression of several sequences from ORF overlaps demonstrated very high levels of frameshifting (198), and regulatory potential merits investigation, keeping in mind that, though the gene is assigned the name magnesium chelatase, binding of something else, perhaps a different cation, could be relevant. Though there is strong potential for structure formation, the sequence involved is not strongly conserved; yet the extent to which the stems are purine-rich on one strand and pyrimidine-rich on the other (the relevant sequence of *Pseudomonas protegens* is CTAGGCATCCGCCCCCGCACTGACCGGGGGGCGGATGCCAAG; nucleotides with potential for stem-loop base pairing are underlined) points to the potential for triplex formation with associated relevance for divalent cation binding.

### Copper ATPase pump and its putative chaperone

Metals are utilized by a substantial proportion of proteins as important catalytic and structural components. However, they are detrimental in excess and mammals exploit this to restrain bacterial infection. For instance, macrophages use copper intoxication to kill bacteria (310), and bacteria have an elaborate system to control copper homeostasis. Commonly, this involves a membrane-integral P-type ATPase, CopA, which pumps copper (and silver) ions from the cytosol at the expense of ATP hydrolysis. In several bacteria, but not in others, e.g. *E. coli*, a separate gene encodes a small soluble copper binding protein (metallochaperone) that transfers copper to its membrane-integral partner. In *E. coli*, a transporter-like function was proposed to be encoded by the 5′ segment of *copA* (311), but how this occurs was not understood.

Ribosome profiling has the potential to reveal frameshifting candidates, and the Supplementary Material of a classic paper on such profiling of *E. coli* identified *copA* as a candidate for a ‘novel translational event’ (312). Follow-up analysis revealed that efficient −1 ribosomal frameshifting does occur and yields a 70 amino acid product which is a putative copper chaperone (S. Meydan, A. Mankin, N. Vazquez-Laslop, personal communication), much shorter than the 834 AA product of standard zero-frame decoding.

### Anaerobic fixation of carbon dioxide and 50% efficient −1 ribosomal frameshifting

Cyanobacteria, considered to be the most abundant photosynthetic organisms (313), and some chemolithoautotrophic bacteria, employ a carbon dioxide concentrating mechanism to overcome the low affinity of the key enzyme, Ribulose-1,5-bisphosphate Carboxylase/Oxygenase (RuBisCO), for CO_2_ and promiscuous activity for competing O_2_ substrate. A key part of the concentrating mechanism is giant organelle-like structures, carboxysomes, of 200+ MDa and 80–500 nm that contain thousands of protomers, and which have some resemblance to icosahedral viral capsids. RuBisCO is sequestered within the protein shell microcompartment. Remarkably just 10 genes from *Halothiobacillus neapolitanus* are sufficient to express the α-carboxysome in the heterologous bacterium *E. coli* (314). One of these genes encodes the cargo proteins carbonic anhydrase and several encode shell proteins. Decoding of one of the genes, *csoS2*, required for carboxysome formation (315), utilizes 50% efficient −1 frameshifting (316). The product of standard decoding, Cso2B, is 869 amino acids and the product of frameshifting, Cso2A has 570 amino acids with its 3 C-terminal amino acids encoded by the −1 frame. Unlike Cso2B it cannot function in forming carboxysome. It has been speculated that the function of having two forms of Cso2 is related to partitioning of RuBisCO, Cso2B functioning primarily underneath the shell and Cso2A within the lumen of the carboxysome. But as Cso2B has only just been discovered, characterization will take some time. However, interestingly Cso2 has functional similarities to foot-and-mouth disease virus protein VP4 which binds to the interior side of the viral capsid (316). Currently 162 species are known with *cso2* genes and 79 are predicted to utilize frameshifting (316), which is therefore important for the global carbon cycle.

### *dnaX* and the 1:1 ratio DNA polymerase products produced by alternative means

The *dnaX* gene of many bacteria yields two DNA polymerase subunits in a 1:1 ratio. The longer product tau (τ) has essentially all the sequence of the shorter gamma (γ), but a C-terminal extension that comprises about one-third of its length encompasses additional functional domains. In *Caulobacter crescentus*, γ is essential and derives from proteolytic cleavage of the longer τ (317). In *E. coli*, after considerable controversy, it has recently been established that γ is part of the DNA polymerase III holoenzyme complex and is important for UV resistance and normal functioning of Pol IV mutagenesis associated with double strand-break repair (318). In a counterpart to the yeast prion [*PSI]+* considered above and below, error-prone polymerases are important for generating mutations for evolutionary fitness (319). Consistent with significance of γ is a diversity of mechanisms for its generation and these are considered next.

Two-thirds of the way through the *E. coli dnaX* coding sequence, 50% of the ribosomes shift to the −1 frame and terminate one codon later to synthesize γ (320–322). In many fewer bacteria, the product derived from ribosomal frameshifting seems to be reversed with τ being derived from frameshifting and the termination codon for γ being in the zero frame directly after the frameshift site (198). One of these candidates that has potential stimulatory signals similar to *E. coli dnaX*, is *Chlorobium phaeobacteroids* (S. Heaphy, J.F.A. and P.V.B., unpublished). Though the expression of *Thermus thermophilus dnaX* is also not like its *E. coli* counterpart, its longer product, τ, is encoded in a single ORF as in *E. coli*. Transcriptional slippage results in a population of mRNAs with extra bases. As there are nearby stop codons in both non-zero frames (with respect to the template DNA), standard translation of those with non-multiples of three extra bases, yields γ, which is again synthesized in a 1:1 ratio to τ (323). In *E. coli*, the shift site involved is highly shift-prone (94,320–322) due to the pairing of the sole tRNA^Lys^ with the second shift-site codon involved being relatively weak (324). However, in *Thermus thermophilus* there are two lysine tRNAs one for each of the two lysine codons, AAA and AAG. As one of these is expected to tightly pair, and so poorly dissociate from AAG, this may be relevant to *T. thermophilus* utilization of RNA polymerase slippage rather than the ribosomal frameshifting used to generate its *E. coli* counterpart.

### Interrupted pili genes

*Bifidobacterium breve* are among the first colonizers of the gastrointestinal tract of human newborn babies and are one of the dominant genera of breast-fed infants. As with many other bacteria, its pili are important for adhesion and colonization. Yet sequencing showed the presence of long pili genes where a single frameshift would be required to give a functional product (325). While ‘pseudogenes’ are a possible explanation, the possibility that they are not pseudogenes but functional genes (‘pseudo-pseudogenes’) utilizing frameshifting is appealing.

### Type III secretion systems

Many different Gram negative bacteria that interact with plants or animals use a Type III secretion system, or injectosome, to inject proteins into host cells for initial hijacking of their functions. Another ca. 25 proteins including transcription activators, are used to build the secretion system. In *Shigella*, which causes human dysentery, the encoding genes are plasmid-borne. After transcribing the first 58 codons of *spa13*, transcriptional slippage yields mRNA that results in 25 to 30% of the product having a C-terminal extension of 112 AA required for secretion system function. The encoding sequence for the end of this extension overlaps the start of *spa32* and transcriptional slippage in *spa13* has a major effect on *spa32* expression (326). Spa32 is very important in the determination of the length of the hollow extracellular needle that forms the injection conduit (327). Spa32's role in length determination is affected by its interaction with Spa40 that also interacts with 5 other proteins including Spa33 and MxiA (328). Expression of *spa33* and *mxiA* also involves transcription slippage but in these cases it yields truncated products (326). In addition, slippage to yield an extended product occurs in the expression of the regulator gene *mxiE* with substantial effects on the expression of the adjacent downstream gene (329). Whether the modulatory effect of the transcription slippage on the ratio of the products is influenced by environmental conditions is unknown. While this type III secretion system mechanism is different from phage lambda GT ribosomal frameshifting, expression of the *Yersinia pestis* and *Y. pseudotuberculosis* YopN-TyeA fusion protein related to function of its type III system is also at the ribosomal frameshifting level (330,331). *Y. pestis*, the etiological agent of the infamous plague, is still a major human problem in several countries (332) and *Y. pseudotuberculosis* is a less aggressive foodborne enteropathogen. After it is transmitted by fleas to warm-blooded animals and the needle part of the type III secretion system senses contact with a host cell, a signaling system influences five proteins to no longer inhibit secretion of the effector proteins that reorganize the host cell. One of these is YopN. However, while the frameshift product has a distinct impact on fitness, the reason is not understood despite extensive work on the complicated system involved (331). Polyamines are linked to controlling the type III secretion systems (333,334) and the prospect that polyamines could influence the frameshifting event leading to YopN-TyeA hybrid synthesis is considered tantalizing (331).

### Archaeal −1 frameshifting

As described above, synthesis of the G–T protein of four viruses very likely involves the translation machinery of their halophilic archaeal hosts mediating −1 frameshifting with expression of archael virus HSTV-2 involving +1 frameshifting (48,49). Also as considered above, frameshifting is likely involved in the synthesis of magnesium chelatase from the archaea *Methanocaldococcus* and *Methanococcus* (198). Other possibilities, such as in *Sulfolobus* (335) also merit investigation.

### ABP140 (alias TRM140)

Many different eukaryotic mRNAs exhibit sub-cellular localization with understanding of its significance being very incomplete and interest in especially the neuron-destined mRNAs being very high. When the yeast *S. cerevisiae* buds several mRNAs are transported to the daughter cells in an actin dependent, but translationally independent, manner by the myosin machinery. However, the conserved N-terminal 17 amino acids of ABP140 is an actin binding domain (336,337) that co-translationally causes its mRNA to associate with actin cables and be transported to the distant pole of the mother cell, rather than to the daughter cell. Unlike much mRNA transport, this trafficking is independent of mRNA structure (338). Zero frame codons 277–279 have a Ty1-like +1 frameshift site CUU_AGG_C (that involves neither Thr nor Ser tRNAs) and codon 280 is a stop codon. The doubtless substantial proportion of ribosomes diverted to the +1 frame decode a 348-codon ORF that specifies a methyltransferase, a major function of which is in formation of the 3-methylcytidine modification at position 32 of the anticodons of several tRNA^Thr^ and tRNA^Ser^ isoacceptors, hence the TRM140 (tRNA methyltransferase) designation (336,339). [Of unknown significance is that zero-frame codons 208–214 specify (DG)STSTTTS and codons 251–261 specify (DD)TTGDTTSSTTS (340) with the latter being just before a +1 frame stop codon at the end of a 171 codon ORF.] The resulting fusion of structural and enzymatic functions yields an indirect linkage of actin to tRNA modification. tRNA position 32 interacts with position 38 on the other side of the anticodon loop (341) with consequences for decoding accuracy (342). The significance of the frameshifting and presumably of location-specific methyltransferase activity is unknown but likely related to its function in the yeast's natural environment and not in lab culture. [Why deletion strains are sensitive to neomycin is obscure (343).] Nevertheless, given the time of divergence of the ancestors of the yeast species that have the characteristics of ABP140 frameshifting, the frameshifting has to have been occurring for 150 million years and be of selective advantage (242). [However, *S. castellii* has lost the frameshift site.] ORF1 is much less conserved than ORF2 which is also conserved in other organisms that do not have fused expression with an upstream ORF.

### Adenomatus polyposis coli (APC)

A computational analysis of sequences from 12 *Drosophila* species using reading frame conservation and codon substitution frequencies showed that in the coding region of the *D. melanogaster* APC gene, purifying selection switched from the zero frame to the −1 frame and subsequently back to the zero frame. It was listed as a candidate for utilized frameshifting in its expression (344). APC functions in the canonical Wnt/*Wingless* (*Wg*) signaling pathway in *Drosophila* and mammals (*Drosophila* APC is implicated in intestinal stem cell proliferation, and in mammals APC mutants are a direct cause of colon cancer). −1 frameshifting was experimentally found to occur 143 nts upstream of the computationally predicted switch of coding frame signatures (46). Zero frame conservation only of the segment 5′ of the frameshift site, and of the segment 3′ of the frameshift product termination codon, implies that the part of the product of standard decoding encoded by the intervening zero-frame sequence merely serves as a linker. Significance of the frameshifting is indicated by conservation of the new frame product. Suggestive of an ancient origin of the frameshifting, it also occurs in nematodes, but interestingly, in that case the C-terminal region of the *trans*-frame product encoded by the −1 frame is much shorter (13 AA versus 125 AA) (46). Immunoprecipitation of human APC reveals a variety of shorter than full-length products but whether any of these are frameshift derived is unknown – and not easy to analyze given the low expression level. However, a single T to A transversion that results in a run of 8 As is relatively prevalent in a specific population group where it is directly associated with colon cancer (345). Both replicational and transcriptional slippage are likely responsible for the causative protein products whose synthesis is prematurely terminated by stop codons that are not in the WT zero frame (345,346).

### The Rab-GDI displacement factor which is at least involved in endoplasmic reticulum to Golgi transport

In *Pezizomycotina* ascomycete fungi including *Podospora anserina*, frameshifting occurs near the end of a modest-sized ORF that is widely conserved even to *S. cerevisiae*. This results in ribosomes entering a longer ORF that is less conserved and only present in *Pezizomycotina*. The frameshifting has been conserved for 400 million years. In most the frameshifting is into the -1 frame, and in *P. anserina* the shift site is U_UUU_UCC with a pseudoknot starting just 2 nts 3′ of the shift site but with variation in the stimulatory sequence. In some, however, the frameshifting is into the +1 frame (347).

### uORF/Leader ORF: antibiotic induced regulatory frameshifting, also *pheL*

Translation of a short upstream ORF (uORF/leader ORF) which depends on the physiological state of the cell, can influence expression of its downstream ‘main’ gene. uORF translation can in effect be a sensor for exogenous factors, such as the presence and concentration of specific metabolites. Though macrolide antibiotics inhibit bacterial translation, bacteria have developed uORF translational systems for sensing very low levels of their presence and activating initiation of synthesis of a resistance determinant. uORF-mediated regulation of *ermC* that renders bacteria resistant to erythromycin and other macrolide antibiotics has been particularly well-studied in part because of the clinical importance of macrolides. The most recent generation of macrolides are ketolides, e.g. telithromycin. While some macrolides cause stalling during uORF translation with resulting prevention of sequestration of the *ermC* initiator, telithromycin instead, in a manner largely independent of nascent peptide sequence, induces a proportion of ribosomes to shift frame. Their continued translation in the new frame into the intergenic spacer unfolds mRNA structure and permits initiation of *ermC* expression (61). Prior to this discovery, the most promising candidate for productive utilization of frameshifting to influence downstream expression via mRNA unfolding had been *pheL*, the uORF for the downstream bacterial phenylalanine biosynthesis gene, *pheA*, (especially Enterobacteria, e.g. *E. coli*). However, the frameshifting that does occur seems to be a by-product of the features responsible for the transcription attenuation control mechanism utilized and to be incidental (19).

### Release factors: Autoregulatory in bacteria, natural prion ‘variants’ in S. cerevisiae, reassignment facilitated frameshifting in multiple Euplotes genes

Bacterial release factor 2 (RF2) mediates termination at UGA and UAA, with its termination at UGA being unique since bacterial release factor 1 mediates termination at UAG and UAA. Before their genes were sequenced the possibility was entertained that the coding sequence for RF2 might have an in-frame UGA stop codon early in the coding sequence with deficiency of RF2 leading to extra slow decoding of UGA that facilitated stop codon readthrough by near-cognate tRNA resulting in synthesis of full-length RF2. However, none of the RF2 genes from the large number of sequenced bacterial genes have such an early in-frame UGA codon (348) with ORF2 continuing in the same frame and the counterpart with UAG and release factor 1 genes is also unknown. [A giant virus of amoeba does have a release factor gene whose expression involves stop codon readthrough and separately ribosomal frameshifting (349).]

Nevertheless, about 87% of bacterial RF2 coding sequences do have an in-frame UGA early in their coding sequence (348). In the *E. coli* RF2 gene it is codon 26 (350). After the short initial zero-frame ORF, the rest and great majority of the coding sequence is in the +1 frame with +1 ribosomal frameshifting being obligatory for RF2 synthesis. At high levels of release factor 2, there is efficient termination at the UGA and the resulting short peptide is rapidly degraded. With progressively lower levels of RF2, there is increasingly more efficient frameshifting at the codon 5′ adjacent to the UGA and restorative synthesis of RF2 (54,351–353). The nt 3′ adjacent to the UGA is nearly always C and, as this disfavors strong termination at the UGA (354), it is important for this frameshifting serving a sensitive autoregulatory function.

The frameshifting cassette is remarkably conserved in diverse bacteria – even making allowance for the relatively small size of the shift cassette, many fewer variants are evident than, for instance, with the antizyme shift cassette. RF2 frameshifting was likely present in a common ancestor of modern bacteria and subsequently lost in several different evolutionary branches though it is hard to infer evolutionary relationship due to frequent horizontal gene transfer among bacteria (355).

RF2 frameshifting involves the anticodon of peptidyl-tRNA dissociating from its codon pairing and following realignment, re-pairing to mRNA at the overlapping +1 frame codon with the involvement of first codon position wobble pairing. Successive errors have been shown to follow from near-cognate P-site pairing, and binding of RF2 to mismatched complexes aided by RF3 leads to termination in what has been termed post peptidyl transfer quality control (356). As frameshifting is elevated when the RF2 level is low, it will be of interest to ascertain whether there is heterogeneity of the amino acid sequence encoded by the codons directly following the shift site (the ‘special’ conformation of the ribosomes due to the signals involved may preclude it). Bacterial release factor 1 does not play the role that release factor 2 does in quality control (357).

One of the soluble proteins prone to conversion to a prion form is *S. cerevisiae* release factor 3, eRF3. In cells with the prion form, [*PSI+*], there is slow decoding of stop codons. This has been demonstrated to lead to elevated frameshifting in the decoding of a gene that requires ‘shifty stop’ frameshifting (in that case there is UGA in the ribosomal A-site), and this has substantial physiological consequences as it lowers polyamine levels (12).

In contrast to transitory decreased termination in bacteria that is alleviated by regulatory frameshifting, there may be a permanent counterpart in the ciliate *Euplotes* (for image see (813)). Studies of specific individual *Euplotes* genes have revealed many where frameshifting is involved in expression, including protein kinases (34,35,358) and the telomerase components described above (31). In *Euplotes*, UGA is reassigned from being a stop codon, perhaps derived by selection as a defence against invading pathogens including in their food (359). It has been proposed that the accompanying alteration of release factor recognition (360) results in poor decoding of UAA (in the ribosomal A-site) thereby enhancing the opportunity for frameshifting when the P-site tRNA has potential for re-pairing in the +1 frame (361,362). Though this is likely relevant, rampant frameshifting is not known in ciliates other than *Euplotes* even though they also feature stop codon reassignment (of UAG in some). Intriguing mysteries abound and the discovery that WT release factor can be involved in 4-base stop codon recognition may be relevant (363,364).

### Amyloid, iterations including triplet repeat expansion neurodegenerative disease and vision mutant dogs that are not blind

With some analogy to the spread of viruses, transcellular propagation of protein pathogens was thought in mammals to be restricted to such diseases as Creutzfeldt–Jakob, scrapie and bovine spongiform encephalopathy (BSE). However, it has recently been realized that the amyloid assemblies of Aβ peptides and of tau proteins in Alzheime'rs disease and the α synuclein assemblies in Parkinson's stochastically spread in a prion-like manner (365). A small proportion of Alzheimer's disease is inherited and due to mutations in the gene encoding amyloid precursor protein. A small proportion of it is encoded transframe with respect to its DNA sequence (366). Further, in both Alzheimer's and Spinocerebellar ataxia 3, after formation of intranuclear aggregates, some ubiquitin B has C-terminal Gly replaced by a Tyr followed by a 19-AA extension encoded *trans*-frame with respect to the DNA (366,367). This aberrant ubiquitin triggers neuronal apoptosis and mitochondrial impairment that likely contributes to at least Alzheimer's progression (368). Though called UBB^+1^ in the publications just cited, in the notation generally used, including here, the C-terminal extension comes from the −1 frame (369). Though the *trans*-frame encoded protein is being studied intensively, the nature of the event involved in its generation needs further investigation (369,370).

In contrast, at least RAN Repeat-Associated non-ATG translation (RAN) is known to be involved in the important expression of all frames of the G-quadruplex forming hexanucleotide expansion of a GGGGCC repeat in what had until recently been considered the 5′ UTR of the mRNA for C9orf72 that is key to the disruption of nucleocytoplasmic transport in the motor neuron disease ALS (371,372). With the neurodegenerative diseases spinocerebellar ataxia 3 (SCA3) and Huntington's, where expansion of the trinucleotide CAG in the standard coding sequence is key to the disease, ribosomal frameshifting, at least, occurs (373–376). The polyalanine containing product of −1 frameshifting is likely very important medically (377) due to the formation of α-helical clusters rather than the amyloid-fibrils of polyglutamine (378). Counterpart frameshifting in Kaposi sarcoma and Epstein Barr viruses is considered above. Since a number of other chromosomal genes, including those for several transcription factors, have substantial runs of repeats (379,380), potential relevance elsewhere and perhaps not even incidental, is possible. In addition to, or perhaps in some cases instead of, potential ribosomal frameshifting, there is the possibility of at least G quadruplexes influencing oncoming RNA polymerase to mediate a certain level of transcriptional realignment that could by standard translation yield products with alternative C-terminal regions. Doubtless this will be a topic for future work.

As introduced above with *dnaX*, transcription slippage in bacteria on runs of 9 As or Ts results in mRNAs with up to 6 extra nts, with translation of the subset of 3 or 6 additional nts generating zero frame encoded product containing one or two extra amino acids. Among other relevant cases of slippage are those in *Shigella flexneri*, human TGFBR2 and ATRX (381), and the endosymbionts *Buchnera aphidicola* and *Blochmannia pennsylvanicus* (244).

Especially interesting for two reasons is an analysis of dogs of different breeds with poly(A) inserts in a gene essential for vision, as it illustrates the potential for iterative effects at multiple levels in gene expression. With an insert giving a poly(A) tract of 28 As the overall total of shifting to the +1 frame with respect to the genomic sequence is 40% (382). Identification of a modifier that has a big effect on the level of frameshifting (383) should be informative. In addition to the long known ribosomal frameshifting on runs of A to an alternative frame, realignment by −3 has recently been described to yield a zero-frame product with an extra lysine (384). There is just a single AAA_AAA_AAA sequence in *E. coli* coding sequence (50-fold less common than extrapolated from the number of AAA codons) [Where ribosomal frameshifting to the +1 frame occurs by shifting either −2 or +1 nt, the frameshift product of the former has an extra amino acid compared to the latter. Retrograde realignment (toward the mRNA 5′ end) has also been demonstrated with a cassette with a derivative of the T4 gene *60* bypassing sequence region; in that case, with coding resumption occurring in a different frame so that one ribosome decoded a sequence segment and, following transient peptidyl-tRNA anticodon: codon dissociation, realigned backwards to read the same sequence in a different frame to synthesize a single product (385).]

### Ribosomal frameshift sites, tRNAs and their balance: an extreme case, *S. cerevisiae*

Significant frameshifting generally occurs at a discrete site, though when occurring at multiple repeat codons as in the decoding of huntingtin, multiple sites are involved (376). Site-specific mRNA pseudouridylation occurs and is regulated by environmental conditions such as nutrient status (386) with ΨAG decoding being distinctive (387). Further, hydroxymethylcytosine performs an essential role in *Drosophila* brain mRNA (388) and there have been numerous studies on *N*^6^-methyladenosine in mRNA (389). However, it is unknown if any utilized frameshifting occurs at an mRNA modification site, or is dependent on ribosome heterogeneity. Frame transitions generally involve realignment. Sites at which ribosomal frameshifting occurs exhibit considerable variation in their inherent ‘shiftiness’. Many studies have shown that one relevant feature for the great majority of 1 or 2 nt ribosomal frameshifting is the balance between propensity for anticodon:codon dissociation and potential for re-pairing to mRNA at an overlapping codon in a new frame with tRNA modifications being relevant (54,324,390–392), as described for one in detail at the end of this section.

The minimal sequence for nearly, but not quite all, forms of −1 frameshifting is a tetranucleotide of the form Z_ZZN and involves codon: anticodon dissociation and the anticodon re-pairing to mRNA at the overlapping −1 codon. Though noted as occurring at the sequence (G_UU)A_*A*AG where substitution of a C with the italicized *A* created a −1 shift site at which the frameshifting compensated for a nearby −1 frameshift mutation (393), A_AAG frameshifting was first extensively studied (394), and later systematically explored, with synthetic constructs (195). Such −1 frameshifting occurs incidentally in *Bacillus* at A_CGA_AAG to give an extended cytidine deaminase (18) and at G_CGA_AAG is productively utilized by IS1222 to synthesize its transposase (189), and with drug-resistant mutant herpes viruses (6). Studies with it showed that the efficiency of this type of relatively low level −1 frameshifting is dependent on the identity of the two flanking 5′ bases. Studies in *E. coli* of these nucleotides in the sequence NNA_AAG focused on the degree of anticodon loop flexibility and base 34 modification of the tRNAs decoding the 5′ codon (an underscore separates the zero frame codons). Codons whose cognate tRNAs exhibited greater wobble propensity gave higher frameshifting levels probably due to increased liberation of the third codon base, A, for the re-pairing of tRNA^Lys^ in the −1 frame (193). *E. coli* tRNA^Lys^ can also promote a single slippage type event with wheat germ ribosomes whereas *E. coli* tRNA^Asn^ can only promote double-type slippage events at its counterpart codon in both *E. coli* and wheat germ systems, illustrating the subtleties involved (395). A different type of experiment provided evidence that peptidyl-tRNA re-pairing is not required to give a very low but detectable amount of product (396).

When the hexanucleotide motif is extended to become a heptanucleotide so that the tRNA decoding the 5′ codon of the pair of adjacent codons also has the potential of re-pairing to mRNA in the −1 frame, then the frameshifting propensity substantially increases. This was discovered in retroviral frameshifting studies (102). The motif has the form X_XXY_YYZ. While XXX often represents any three identical nucleotides, there are intermediates with the hexanucleotide situation just described; e.g. X_XX is G_GU in phage T7 gene 10 decoded by bacterial ribosomes (397), cardioviruses, e.g. encephalomyocarditis virus (58) and some luteoviruses; G_GA in many insect specific flaviviruses (119,123,124), some umbraviruses and dianthoviruses and in mesoniviruses (398); G_UU(A) in equine arteritis virus (104,134); C_CA(A) in IS3 (399) and U_CC in some members of the Japanese encephalitis serogroup of flaviviruses (70,124). Initial studies in the contexts of retroviral frameshifting and coronaviral frameshifting showed that among the most shifty −1 heptanucleotide sequences with mammalian cytoplasmic ribosomes are A_AAA_AAC, G_GGA_AAC, U_UUA_AAC and G_GGU_UUU (62,104). Following on from the shiftiness described in the last paragraph for A_AAG in *E. coli*, further work with synthetic constructs, also in *E. coli* showed that A_AAA_AAG is much more shift-prone than A_AAA_AAC, A_AAA_AAA, U_UUU_UUA or U_UUU_UUU (94). Shortly afterward A_AAA_AAG was found to be the naturally utilized shift site for *E. coli dnaX* frameshifting (320–322). Approximately half of known bacteria including *E. coli*, have just one lysine tRNA for decoding both AAA and AAG. Its wobble position, base 34, S, which pairs with the third codon base, is U hypermodified with 5-methylaminomethyl-2-thiouridine. While the conserved base 3′ adjacent to the anticodon, N6-threonylcarbamoyladenosine (t^6^A), is critical for recognition of codons with first position A, it is the modification of U34 which is important for discrimination against asparagine codons, AAC and AAU, and also AUA, (400) and refs therein. NMR studies showed that these two modifications remodel an otherwise dynamic loop (401). X-ray crystallographic studies with 70S ribosomes and long mRNA revealed a novel type of base-pairing interaction with third codon base G that broadens known wobble position geometries. In it the modified U is moved toward the minor groove in contrast to standard Crick-proposed wobble geometry in which the pyrimidine is displaced toward the major groove of the codon:anticodon minihelix (400). This work highlights the role of the larger subunit, in *E. coli* ribosomes helix 69, for positioning and stabilization of the anticodon loop (helix 69 forms intersubunit bridge B2a relevant to regulation of groove monitor A1492 (402)). Further, the work is regarded as important evidence for their proposition that steric complementarity is predominant over the number of hydrogen bonds for third base discrimination (400). As the degree of stability of third codon position pairing is highly relevant to the codon:anticodon dissociation central to at least most frameshifting, several earlier discussions about frameshift sites and in particular the less restrictive third codon position, were likely overly focused on hydrogen bonding.

In *E. coli*, in addition to A_AAG 3′ of A_AA being a −1 shifty heptamer, C_CCA_AAG and G_GGA_AAG are also highly shift-prone (195). The shift site for the −1 frameshifting in *g-t* expression in the viruses HCTV-1, -2 and -5 that infect halophylic archaea are G_GGA_AAC, A_AAA_AAC and G_GGA_AAG respectively (49), a mixture of bacterial and mammalian-like second codons.

The initial proposal for heptanucleotide −1 frameshifting involved tRNAs in both the ribosomal P- and A-sites shifting simultaneously before the peptidyl transfer reaction (102). However, it was quickly instead proposed that the shift occurs after peptidyl transfer and perhaps even during translocation (94), and numerous proposals have since been advanced including sequential slippage. The greatest departure from the notion of simultaneous −1 shifting is that just peptidyl-tRNA is involved in the slippage process with the incoming aminoacyl tRNA having the opportunity to also pair in the −1 frame. Experimental evidence for this has been obtained with a variant of the *E. coli dnaX* −1 shift cassette (403). However, discussion of the relationship of that study to other recent biophysical studies and their combined implication are delayed until after the description of stimulators below, since they are key to the non-canonical ribosome states critical for understanding what happens at the shift site.

With the *E. coli dnaX* shift site sequence, A_AAA_AAG, the sole tRNA^Lys^ has the potential to pair with AAG or the −1 frame AAA, with pairing to the latter being stronger. There is no amino acid level distinction between two tRNAs pairing, dissociating, realigning and both re-pairing to mRNA in the −1 frame from the entering aminoacyl tRNA pairing directly in the −1 frame. However, with U_UUU_UUA, the HIV shift site, cognate tRNA^Leu^ dissociating from UUA and re-pairing to mRNA at the overlapping −1 frame UUU would result in a different amino acid compared to acceptance of incoming aminoacyl tRNA^Phe^ into the A-site following a −1 shift involving the tRNA that had paired to zero frame UUU. Indeed the protein product from −1 frameshifting on the HIV shift site is heterogeneous with the U_UUA encoding 20–30% Phe and 70–80% Leu (94,404) and analyzed in depth (97). [Only a trace of a product corresponding to the minor product is seen with human T-cell lymphotropic virus type 1 (HTLV-1, also known as human T-cell leukemia virus) *pro-pol* frameshifting (83), and an unspecified level of counterpart was detected with alphavirus frameshifting (137).] Further HIV frameshifting product heterogeneity comes from a proportion of the frameshifting being −2 rather than −1. Though this has only been detected so far in reticulocytes lysates and transfected cells (98), it is likely also in infected cells.

The identity of the 3 nucleotides, GGG, 3′ of the HIV shift site codon UUA are important for frameshifting efficiency, and it has been proposed that a part of the frameshifting to give the Leu-containing product occurs while the GGG is in the (perhaps distorted) A-site (405). The umbravirus, pea enation mosaic virus, shift site 5′G_GAU_UUU (_UGG_UAG) has a stop codon 3 nts 3′ of it. Changing the UAG to another stop has no effect on frameshifting but changing it to a sense codon has a drastic effect that is not attributable to effects on a 3′ structure (406).

Depending on the realignment possibilities present, ribosomal frameshifting can be prone to occur when the A-site codon is slow-to-decode, due to limitation of the relevant aminoacyl-tRNA. Arising from his group's studies of amino acid starvation on single-nucleotide frameshifting (407–409), Jon Gallant referred to such codons as ‘hungry’ codons. A slow-to-decode A-site is more generally important for +1 frameshifting than it is for −1 frameshifting. A very interesting occurrence involves the expression of Ty elements in *S. cerevisiae* and related yeasts. The Ty1 shift site, CUU_AGG_C on its own mediates a remarkable 40% +1 frameshifting. The *S. cerevisiae* shift site for *ABP140* is identical and that for *EST3* (CUU_AGU_U) is similar. The anticodon of the tRNA^Leu^ that decodes CUU in the Ty1 shift site is ^3′^GAU^5′^ which has its wobble base 34, U, unmodified. This allows the tRNA *in vivo* to read all 4 CUN codons including CUU (410). Where U:U apposition is involved, it is akin to the 2-out-of-3 reading identified elsewhere (411,412). Poor zero-frame codon pairing by peptidyl-tRNA^Leu^ facilitates dissociation permitting re-pairing to mRNA at the overlapping +1 frame UUA. Slow recognition of A-site AGG by low abundance cognate tRNA^Arg^ (anticodon ^3′^UCC^5′^) facilitates the frameshifting (209,210). The high abundance of tRNA^Gly^ (anticodon ^3′^CCI^5′^) (413) cognate for the overlapping Ty1 +1 frame codon, GGC, is also important for efficient frameshifting. The implied competition for A-site decoding is mechanistically informative (414).

**Figure 8.**
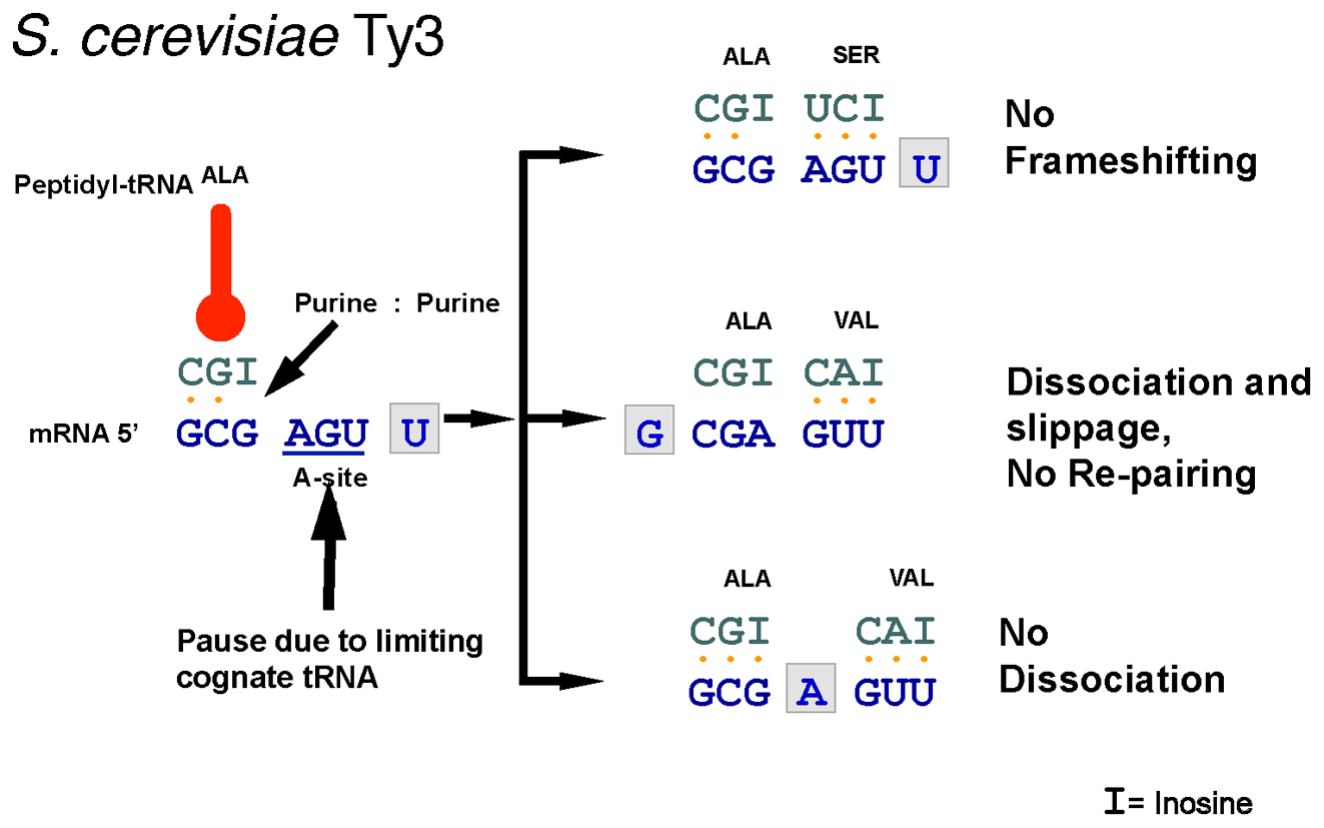
Absence (or minimal) potential for re-pairing to mRNA at an overlapping +1 frame codon in *S. cerevisiae* Ty3 frameshifting. Biophysical studies are needed to determine whether the tRNA^Ala^ anticodon ^3′^GCI^5′^ (where I is inosine) dissociates at the time of frameshifting. Only a small number of codons can substitute for GCG, including CGA (422). See text and (453, 814) for discussion of this and other candidate occurrences.

While the *S. cerevisiae* Ty3 shift site (GCG_AGU_U) also features a slow-to-decode A-site codon, AGU_U is less effective than its Ty1 counterpart and the 5′-adjacent codon in Ty3 is GCG instead of CUU (214,414) (Figure 8). As discussed in relation to Ty3 frameshifting (415), *S. cerevisiae* is unusual in lacking a tRNA^Ala^ (anticodon CGC) that provides a good match with codon GCG which is instead decoded by tRNA^Ala^ (anticodon ^3′^CGI^5′^). While inosine pairs strongly with U and C, its apposition with A allows weaker decoding (416,417) and its purine:purine apposition with G is surely relevant to the frameshifting. Discussion of potential relevance of I pairing through its Watson–Crick side with the Hoogsteen face of A with the A base in the syn conformation with respect to the ribose, and counterpart I:G pairing (418) has been revived (387,419) and may be pertinent. (The IS1222 frameshifting mentioned at the start of this section also involves wobble position I:A apposition.)

Unlike Ty1, with Ty3 the peptidy-tRNA does not have realistic standard pairing to the +1 frame codon as C:C, G:G, I:A would be involved. One interpretation is that slippage is not involved but there is some level of occlusion of the 3′ base, A, facilitating incoming aminoacyl-tRNA pairing with GUU rather than AGU (214,414). Alternatively, there may be peptidyl-tRNA dissociation but not re-pairing to mRNA following slippage (420). [The different 3′ stimulators for Ty3 and EST3, see below, can act largely indiscriminately on Ty1 and Ty3 shift sites (241,421).] Regardless, it is clear that Ty3 frameshifting yields Ala Val (422). Surprisingly *S. cerevisiae* antizyme frameshifting (shift site GCG_UGA_C (273)) also shows +1 frameshifting even though there is –2 re-pairing potential (423). In *Kluyveromyces waltii* yeast, which unlike *S. cerevisiae* does have tRNA^Ala^ (anticodon CGC), the shift site is CCG_UGA_C. However, it lacks a corresponding tRNA^Pro^ (anticodon ^3′^GGC^5′^) (423). Despite *S. cerevisiae* Ty3 and antizyme shift sites both having GCG, when both are tested in the same reporter system, and extrapolated from measurements in the absence of Ty or antizyme context effects, the intrinsic shiftiness of GCG_UGA_C (antizyme) is 37% and GCG_AGU_U (Ty3) is 2%, showing the greater effectiveness of the stop codon (214,414). [For comparison the shiftiness of CUU_AGG_C (Ty1 and ABP140) is 43%.] Correspondingly, as considered below, in its natural yeast context antizyme has inhibitory modulators while Ty3 has a stimulatory modulator.

As extrapolated from the extent of its conservation, antizyme, *EST3* and *ABP140* frameshifting has been conserved in budding yeasts for about 150 million years (242,423). However, the possibility that the frameshifting may have driven selection pressure on the tRNAs present needs to be treated cautiously since the tRNA population in *S. cerevisiae* is distinctive in ways other than considered above, though fortuitous for a search for new occurrences (424). Despite the caution, the nature of the tRNAs present is likely to have influenced the frequency of +1 shift prone heptanucleotides since the sequences used for Ty1, Ty3 and certain others are strongly under-represented in *S. cerevisiae* coding sequences (424).

In *E. coli*, when tandem rare codons, e.g. AGG_AGG or AGA_AGA, with correspondingly sparse tRNAs occur, frameshifting has been detected. When mRNAs containing such sequences are over-expressed, for instance when certain heterologous (mammalian) genes were expressed from multi-copy plasmids for biotechnological purposes, up to 50% frameshifting was detected (425). However, with low mRNA levels the amount of frameshifting is greatly reduced, i.e. the amount of frameshifting is mRNA expression level dependent (426,427).

Limitation of a specific aminoacyl-tRNA can also occur in certain triplet repeat expansion situations. With disease level expansion of CAG repeats in huntingtin, frameshifting at multiple sites has been demonstrated, but in this case it is difficult to distinguish the relative effects of aminoacyl tRNA limitation from a stimulatory effect of a 3′ structure formed from the repeat sequence (376). This study importantly illustrates significance of relative expression levels of a relevant tRNA in different brain tissues. An independent study showed that mutation in a brain-specific tRNA causes neurodegeneration (428). A subset of aminoacyl-tRNAs are tightly associated with translating ribosomes (429,430). To what, if any, extent this is relevant to CAG repeat, and other, frameshifting, is unknown. The issue is to what extent localized aminoacylation limitation for a ribosome translating, for instance a run of repeat codons, is a reflection of overall cellular limitation for that aminoacyl tRNA. In addition to extreme situations, such as when silk fibroin is being synthesized in silk glands, the pool of aminoacyl tRNA isoacceptors changes when different classes of mammalian mRNAs are being translated (431). However, for the only virus tested where frameshift utilization is known, influenza A virus, it is the particular relevant local pool of isoacceptors that is selected rather than the tRNAs overall being adjusted (432). Though some viruses encode their own tRNAs, whether any are relevant to frameshifting is unknown.

Natural perturbation of aminoacyl-tRNA levels occurs under conditions of amino acid starvation, a common situation in nature especially for a high proportion of bacteria, and is exacerbated in *relA* mutants when tRNA undermodification is more prevelant (433). The effect of this on frameshifting has been studied extensively in *E. coli* (409). What is unclear is under natural conditions, the extent to which such ‘hungry codon’ frameshifting leads to out-of-frame termination with subsequent proteolysis contributing to recycling of scarce amino acids for the synthesis of proteins that are vital under such conditions. [While this type of frameshifting could contribute to the synthesis of *trans*-frame encoded proteins that have novel function specific for starvation conditions, no examples are known.]

Almost all tRNAs have standard anticodon loop sizes. Among the few exceptions are a mitochondrial tRNA from yeast *S. cerevisiae* (434), tRNAs in a phage (435) that has many relatives, (436) and a mitochondrial tRNA from the glass sponge *Iphiteon panacea* (252). The *S. cerevisiae* tRNA has an extra base in its anticodon loop but neither it nor the other two, are known to mediate frameshifting. However, frameshifting does occur naturally in expression of several mitochondrial genes in *Polyrhachis* species and *I. panacea*. One of the *I. panacea* mitochondrial tRNAs whose anticodon loop very likely has 9 nts is tRNA^Gly^ and it has 4 bases in the anticodon location that matches the UGGA codon at which the shift to the +1 frame occurs. The frameshifting properties of mutant tRNAs with 9 or 10 nt anticodon loops has been studied in *E. coli* with spontaneously arising mutants (437) or ones sought in code expansion studies (438); though enlarged anticodon loops do not necessarily imply other than triplet pairing inside WT ribosomes (439,440), the use of the anticodon loop of a tRNA switching stacking to present an alternative offset triplet anticodon has been proposed (441). However, even if quadruplet pairing or an anticodon loop ‘roll’ occurred in a few cases of mitochondrial frameshifting, it would be exceptional and not the general explanation. Nevertheless, while much of the natural frameshifting in mitochondrial expression to circumvent potential deleterious consequences of mutational inserts follows the same principles as just described, some of the features are mitochondrial specific. At most of the insert sites in *Polyrhachis* ants, the zero-frame codon 3′ adjacent to that for the peptidyl-tRNA^Gly^ involved in the switch are AGY codons. Its cognate tRNA has a characteristic restricted to a subset of mitochondrial tRNAs in that it lacks the DHU stem, and has a distinctive structure. Perhaps it is slow-to-decode. The +1 frameshifting in decoding several genes in glass sponges occurs at extremely rare UGG_(A), or CGG_(A) codons (with all the inserts being T, or rarely C, 5′ adjacent to a GGA codon). Though most of their frameshifting has been similarly interpreted to that of Ty3 (252), other possibilities merit consideration (mitochondrial ribosomes also have an E-site, despite an earlier proposal to the contrary, and so dissociation potential of the corresponding codon: anticodon interaction may be relevant).

Apart from in mitochondria, identification of +1 shift sites is often more difficult than spotting tandem -1 shift sites. An early surprise was in bacteria even without an adjacent 3′ stop codon, the tetranucleotide UUU_Ynn is especially prone to +1 frameshifting (17,442), and appears to be the site for the *Y. pestis* frameshifting yielding the functional YopN-TyeA product (330).

As described above, the influenza A virus minimal frameshift cassette, UCC_UUU_CGU has been used to tentatively identify the shift site of previously identified occurrences of viral frameshifting where the precise location and identity of the shift sites involved was obscure. Utilization for searches of cellular gene utilized frameshifting is also possible (125). Mutating the UCC codon to AGC (serine) or to GGG, CCC or AAA resulted in a 40 to 70% reduction in the frameshifting efficiency (39), giving credence to the possibility that UCC plays an important positive role in the E-site or at least 5′ of the codon to which peptidyl-tRNA is paired at commencement of the shift (125). Though it is somewhat of a ‘special’ case because of the nature of its stimulatory element (see below), prior work on *E. coli* release factor 2 frameshifting lead to the proposal that weaker ‘E-site’ pairing disturbance favored higher +1 frameshifting levels (443–446). However, though likely different for −1 frameshifting, E-site relevance was also demonstrated for HIV (405,447,448), pea enation mosaic virus (406) and other cases of frameshifting. Additionally, in yeast, bioinformatic evidence suggests that when X_XXY_YYZ is in the P and A ribosomal sites, the identity of the two nucleotides immediately upstream in the E site are highly biased, suggesting an extended signal (449). Fluorescence and FRET studies either involving single ribosomes or ensemble approaches with the stopped-flow technique showed that E-site dissociation is delayed on ribosomes stalled by the *E. coli dnaX*-, or a coronaviral, 3′ −1 frameshift stimulatory element (403,450). Nevertheless, single molecule studies with zero-mode waveguide tracking of ribosomes show that the E-site tRNA is released before the ribosome rotates backward, both with *E. coli dnaX* −1 frameshifting and especially with T4 gene *60* bypassing (403,451).

**Figure 9.**
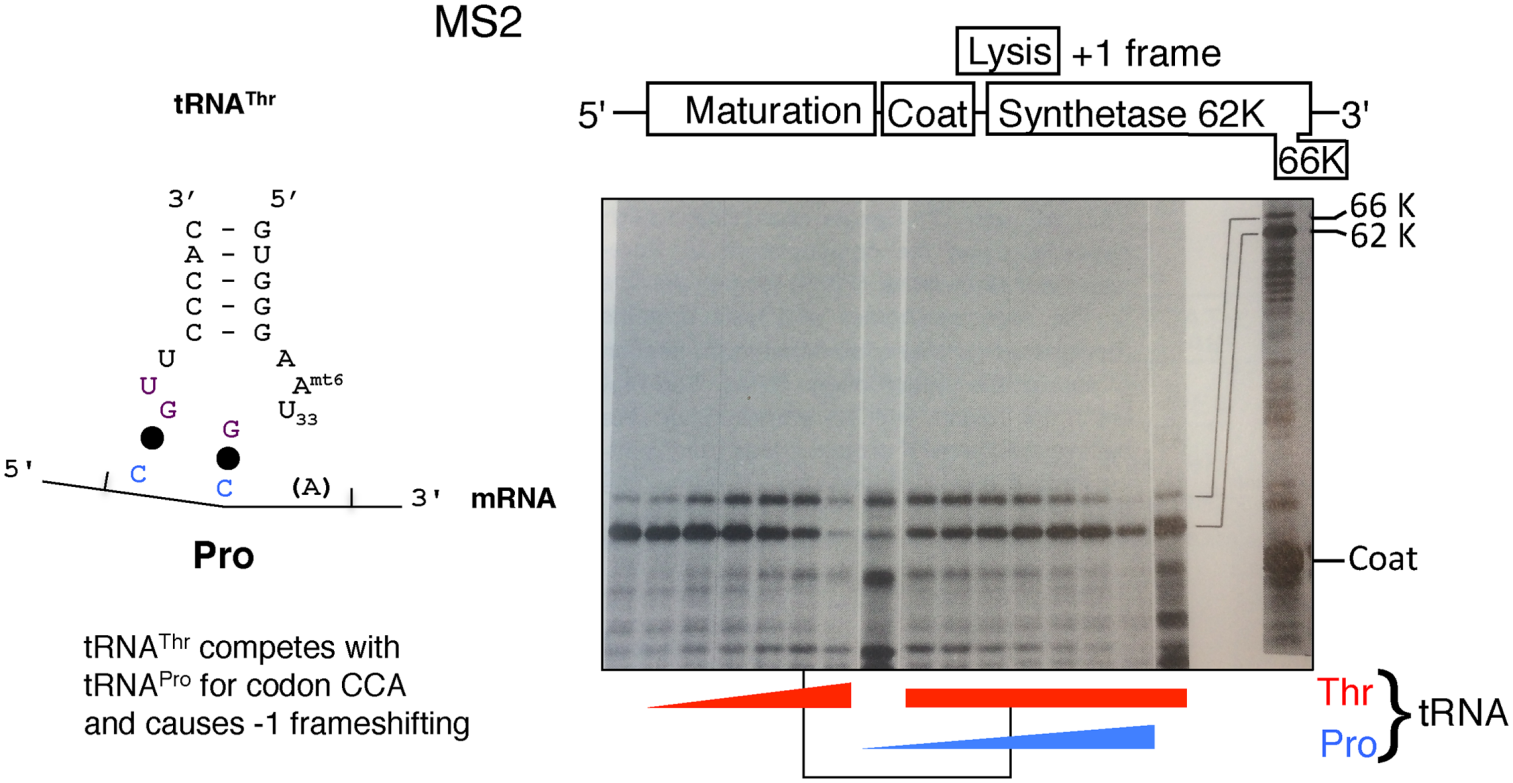
Non-cognate frameshifting. In extracts of *E. coli* with unperturbed tRNA balance, tRNA^Thr^ decodes a proline codon to cause −1 frameshifting that results in synthesis of a 66 K form of the viral encoded component, synthetase, of replicase. Increasing the relative amount of tRNA^Thr^ (red wedge) increases the proportion of 66 K with respect to the product of standard decoding (62 K). With elevated levels of tRNA^Thr^ (uniform thickness red line) increasing the amount of cognate tRNA^Pro^ (blue wedge) decreases the relative amount of 66 K. Anticodon bases 35 and 34 are complementary to the first and second proline codon bases. The normal anticodon of tRNA^Thr^ is in purple. A model with 1:6 anticodon loop stacking is discussed in (453). Reprinted in part from Atkins, J.F., Gesteland, R.F., Reid, B.R. and Anderson, C.W. (1979) Normal tRNAs promote ribosomal frameshifting. Cell, 18, 1119–1131 with permission from Elsevier.

With delayed or sub-maximal A-site pairing, and dissociation of at least peptidyl-tRNA pairing being important for, at a minimum, the majority of productive frameshifting cases, thoughts turn to the extremes of where either the frameshifting is completely non-cognate or where the mRNA slides freely. Non-cognate −1 frameshifting was shown in an early *in vitro* study that revealed frameshifting significance of the balance of tRNA. With an unperturbed balance of tRNAs, an ACC decoding tRNA^Thr^ was shown to read a CCN proline codon to generate an elongated form of phage MS2 synthetase (73,75) (Figure 9). The identity of just 4 anticodon loop bases is important for this frameshifting (452), but distinction between two alternative mechanisms remains unresolved (453) and whether there is relevance for other cases of frameshifting remains to be seen. [The only other tRNA found in that study to mediate non-cognate frameshifting was a tRNA^Ser^ and it is these two tRNAs that decode ΨAG codons (387,454).] The best known case of a different type of extreme recoding involving mRNA sliding, is in decoding phage T4 gene *60*. Peptidyl-tRNA dissociates from codon 46, GGA and re-pairs to mRNA 47 nts 3′ at GGA with coding resuming at the 3′ adjacent UUA, codon 47. This and other cases involving stimulators are considered below. However, sliding on runs of A has recently been reported (384,455).

This section on shift sites will conclude with the autoregulatory frameshifting in bacterial release factor 2 synthesis. The highly conserved shift site is CUU_U, even though re-pairing to mRNA in the +1 frame involves first position G:U pairing. Other shift sites have doubtless been sampled repeatedly over the course of evolution, and UUU_U does occur in a small minority of species. Significantly, in the release factor 2 genes that do not utilize frameshifting in their expression, the first position of the codon corresponding to the CUU is not conserved (443). The most extensive substitution analysis of the CUU shift site, 32 variants, was performed with the 3′ adjacent codon being UAG rather than UGA (390), though the values for the 6 substitutions that are the same as those used in an earlier smaller study with UGA (54), are consistent. CUU_U was found to be by far the most efficient shift site raising the possibility that the simple, m1G, modification of the anticodon base 3′ adjacent to that in the cognate tRNA^Leu^ that pairs with the first codon base C, is important for the much higher levels than with UUU_U where the counterpart base 37 of the cognate tRNA has the bulky adduct threonine in carbamoyl linkage to the 6-amino group of A37 (390). While elaborate modification of base 37 of tRNA^Phe^ helps restrain first position wobble in standard decoding, and first position wobble would not be relevant with a UUU_U shift site, nevertheless its presence may be inhibitory for frameshifting. Several studies, including (354), have shown that the identity of the base 3′ adjacent to the ORF1 stop codon UGA is also very important, and a C at this position is highly conserved.

## STIMULATORS OVERVIEW

With the exception of specialized cases such as *Euplotes* and *S. cerevisiae* Ty1/antizyme-like +1 frameshifting, all high level frameshing including much productively utilized frameshifting, involves stimulatory elements that enhance the proportion of ribosomes that shift frame at the shift site. Stimulators can act singly or in combination with other recoding signals whose effects are either inhibitory or stimulatory on their own with structural transitions and the degree of spacing of oncoming ribosomes being important in several cases. They can act synergistically as illustrated by IS911 where one of the stimulators also serves a role independent of frameshifting (194). Stimulators can act in a variety of ways involving (i) certain interactions of the newly synthesized nascent protein chain with the ribosome peptide exit tunnel on its way to the exterior of the ribosome, (ii) pairing of the mRNA with rRNA close to the mRNA exit path, (iii) mRNA structure 5′ of the recoding site stimulating bypassing and perhaps also being able to stimulate at least +1 frameshifting, (iv) likely mRNA pairing with rRNA close to the mRNA entrance channel, and (v) intra-mRNA structure 3′ of the shift site. *trans*-acting factors include polyamines, protein release factor competing for the A-site, protein binding to a 3′ mRNA sequence and miRNA. While codon usage (456) and pause sites further upstream could influence ribosome ‘traffic’ spacing and so potentally frameshifting efficiency, this has been established for initiation rate. HIV encoded Tat interaction with Tar in the 5′ UTR can influence initiation frequency with substantial consequences for downstream frameshifting efficiency (457), though the existence of a relevant IRES in the 5′ UTR has been challenged (458).

In certain circumstances herpes viruses avail of a parallel, and dramatic 100-fold, effect on frameshifting efficiency of not having a stop codon on the new frame (6). In this case the spacing of ribosomes is relevant, though how is unknown. However, the issue is more general as the influence of ribosome spacing, mostly but not exclusively dictated by ribosome loading rate at initiation, on diverse instances of frameshifting is relatively under-studied despite its likely relevance.

Though the shift sites in various cases of productively utilized ribosomal frameshifting vary in intrinsic shiftiness, the final level of shiftiness they exhibit in combination with a recoding signal is what is selected, and there may be specificity involved rather than the components acting as modular ‘plug-in’ cassettes.

### Relevant amino acids encoded by the shift site and as part of nascent peptide frameshift modulators - Polyamines

Certain C-terminal amino acid sequences, e.g. in bacteria Asp-Pro, contribute to an unusually long pause at termination, and some combinations, also involving proline, have marked effects during the elongation phase of protein synthesis (459). While prolonged pausing in bacterial decoding can facilitate ribosome rescue by tmRNA/SmpB and ArfA, it is not in direct competition with the frameshifting (or readthrough) that pausing can also facilitate (460). While the pausing aspects are considered below, the relevant fact here is that amino acid identity and not just shift site: anticodon interactions, can be important.

Frameshifting is utilized in the decoding of phages PSA, A118 and A500 of the food pathogen *Listeria* which is low GC and where only 5% of the proline codons are CCC. [At least in *Salmonella*, the levels of tRNA^Pro^ decrease under conditions of hyperosmotic shock and tRNA^Pro^ levels regulate a key gene for pathogenicity that is encoded just upstream of a magnesium transporter (461).] For the *tsh* gene of all three *Listeria* phages, utilized +1 shifts are at (AAA) CCC_UGA and additionally in the PSA *cps* mRNA at the sequence ACA_CCC_(UCC_GAA) (153,154).

Another reported early was the carlavirus, potato virus M (462). However, as there are reasons for caution (463), this will not be considered further here.

Frameshifting at the last codon of the leader peptide encoding sequence, *pheL*, of an *E. coli* K12 mRNA central for phenylalanine biosynthesis occurs at 15%. However, shifting at the site involved, CCC_UGA, only occurs at 1.9% in *Salmonella enterica* – possible reasons for the difference are discussed in the original publication (19). In *E. coli* K12, not only is peptidyl-tRNA^Pro^ relevant to the level of frameshifting, the identity of the penultimate amino acid, phenylalaine, is significant and that of the amino acid encoded 10 codons 5′ is also relevant. So specific amino acid effects occur not just at the time of polymerization but well within the ribosome peptide exit tunnel. The frameshifting in this case appears an incidental accompaniment of features important for transcription attenuation. Still it is interesting that when tested in *E. coli* K12, the nascent peptide signal utilized to promote the efficient bypassing of 50 nucleotides in decoding phage T4 gene *60* (considered below) stimulated 60% +1 frameshifting at the CCC_UGA (19). Though CCC_UGA is not known to be utilized for frameshifting in *E. coli*, it is not under-represented in K-12 with 19 genes terminating with it and in half of them the frameshifting occurs at more than 1% (464).

**Figure 10.**
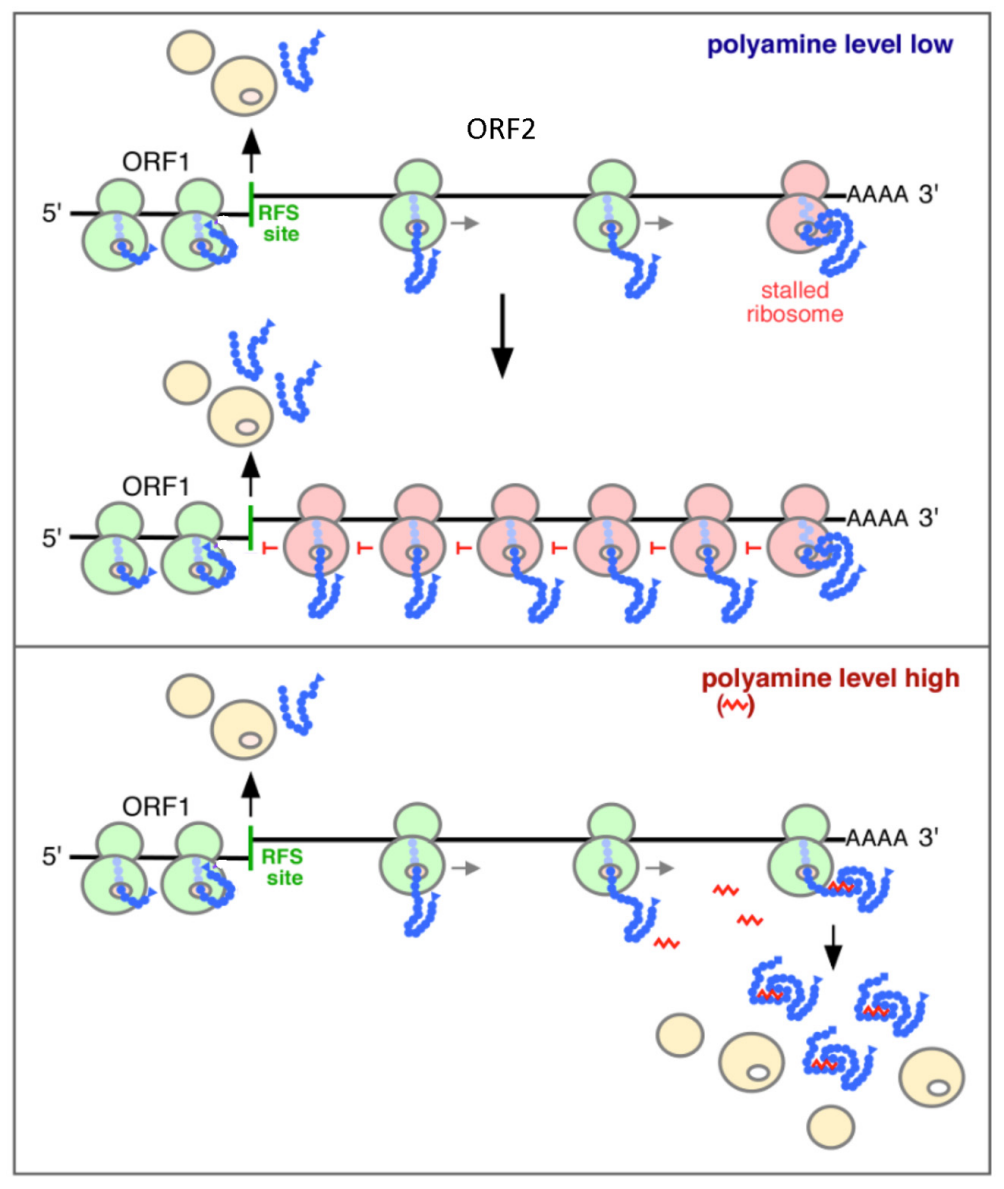
Scheme for *S. cerevisiae* antizyme mRNA +1 frameshifting. The top box represents low cellular polyamine concentrations, where ribosomes pause at the frameshifting site (RFS) with the ORF1 stop codon in the ribosomal A-site. Some terminate, while others continue translation in the +1 frame. An inhibitory element near the N-terminus of the nascent peptide, encoded near the start of ORF1, keeps frameshifting rates low. When a ribosome approaches the 3′ end of ORF2, a newly synthesized part of the nascent peptide, causes this ribosome (pink) to stall. Following translating ribosomes (green) encounter the stalled ribosome and their movement is also blocked (line 2 with multiple pink ribosomes). When polyamine levels are high (lower panel) the nascent peptide encoded near the 3′ end of ORF2, binds polyamines and is then unable to cause stalling. This allows the ribosome containing it to proceed to termination and release of functional antizyme. Reprinted by permission from Macmillan Publishers Ltd: Kurian,L., Palanimurugan,R., Godderz,D. and Dohmen,R.J. (2011) Polyamine sensing by nascent ornithine decarboxylase antizyme stimulates decoding of its mRNA. Nature, 477, 490–494.

Antizyme mRNAs with the CCC_UGA shift site have naturally emerged in evolution but are in a tiny minority as only two were known at the time of an extensive survey (270,293). For the substantial majority of antizyme genes the +1 shift site is UCC_UGA. When CCC_UGA was tested in place of UCC_UGA in a specific cassette in mammalian cells, significantly reduced frameshifting was monitored (465). Of greater interest is nascent peptide relevance. As just discussed, the *S. cerevisiae* antizyme shift site GCG_UGA_C, is anomalously shift-prone. When accompanied by its flanking sequence the frameshifting level of 65% has been measured (268). In contrast to organisms other than budding yeast, dynamic regulation is achieved by controlled reduction of the frameshifting level instead of controlled enhancement. As the discoveries on *S. cerevisiae* antizyme regulation by Dohmen and colleagues (268) are not only intriguing in their own right, their implications for the mechanism in other organisms make it desirable to consider first (Figure 10). At low polyamine levels inactivation of the frameshift inhibitory signals leads to a 20-fold increase in antizyme synthesis. A crucial inhibitory component is a nascent peptide encoded near the end of the coding sequence and only synthesized by a ribosome that frameshifted 669 nts (223 codons) upstream. At low polyamine levels, this nascent peptide interacts with the ribosome peptide exit tunnel to stall the ribosome. This blocks progression of following ribosomes and so of antizyme synthesis. However, at high levels polyamines bind to the nascent peptide precluding it from causing a stall. For the leading ribosome to stall at low polyamine levels, there has to be adequate distance from the following ribosome and the frameshifting provides a partial ‘choke’ to decrease ribosome density and increase the spacing. [An additional more mysterious larger structural element specified upstream of this inhibitory signal but downstream of the frameshift site is also relevant.] A second inhibitory element is also a nascent peptide sequence, but it is encoded so far upstream of the frameshift site that it is expected to be well outside the ribosome at the time of its modulatory action. Changing the Ile encoded by codon 5 to Phe had a major effect and inactivation of the signal resulted in frameshifting efficiency increasing to 62%. Though it acts independently of the distal element, it has been proposed to facilitate the frameshifting pausing involved in adjusting downstream ribosome number for the distal sensor (268). How it acts is unknown.

One of the intriguing facets is that increasing ribosome spacing by changing initiation rates to various degrees did not recover the full 4-fold polyamine induction observed with the wild-type construct bearing the ribosomal frameshifting site. This was taken to indicate that regulation of ribosome density is not the only relevant effect caused by the ribosome frameshifting site (268). We will return to this shortly, see also Table 2.

**Table 2.** Amino acids encoded by codons eight to five 5′ of the frameshift site in diverse antizyme mRNAs. The antizyme 3 frameshift region is very different from all others in that it appears to lack all known stimulatory signals, and in heterologous mammalian cells gives a very low level of frameshifting. The identifiable signals in Zebra fish, *D. rerio*, retina and brain specific antizyme mRNA are intermediate in potential strength. For those mRNAs with a pseudoknot frameshifting stimulator 3 nts 3′ of the shift site, the pseudoknot is expected to be at the unwinding site and at the outer edge of the mRNA entrance channel when codons −2 to −4 are at the ribosomal P-site. For Cardiovirus -1 framshifting see Figure 3B).

		**−8**	**−7**	**−6**	**−5**	
Saccharomyces cerevisiae		G	G	F		
Schizosaccharomyces pombe	P	A	G	G	A	
Coprinopsis cinerea (Mushroom)		S	G	G	P	
Caenorhabditis elegans		P	G	D	V	G
Drosophila melanogaster		G	V	G	P	
Danio rerio – long form (fish)	P	C	P	G	P	
Danio rerio – short form		G	P	G	P	
Danio rerio – brain & retina		A	P	G	P	
Xenopus laevis (frog)		G	P	G	P	
Gallus gallus (bird)		G	P	G	P	
Mus musculus antizyme 1		G	P	G	P	
Homo sapiens antizyme 1		G	P	G	P	
Mus musculus antizyme 2		V	P	G	P	
Homo sapiens antizyme 2		V	P	G	P	
Mus musculus antizyme 3		P	C	S	C	
Homo sapiens antizyme 3		P	R	S	C	
Saffold cardiovirus						
−1 frameshifting	T	N	P	G	P	

The key feature of this control mechanism is a metabolite, polyamines, binding to a nascent autoregulatory sensor protein to regulate the decoding of its own mRNA. One aspect of its relevance to the non-budding yeast cases considered next is that polyamine binding assays done in parallel with *S. cerevisiae* antizyme shows that human antizyme 1 also directly binds polyamines. So even where the intrinsic level of frameshifting is much lower and stimulators rather than inhibitors are used, direct binding of polyamines to nascent peptide has the potential to be generally important for the regulatory aspect.

So far the only evidence for this comes from a study of antizyme +1 frameshifting in the Agaricomycotina class of *Basidiomycota* fungi. A nascent peptide encoded between codons 14 and 4 5′ of the shift site, UUU, is highly conserved at the protein identity specific level (270,466). It is an important stimulator and serves a polyamine-sensing role for modulating antizyme frameshifting levels and so for the autoregulatory circuit (466). It is perhaps even likely that part of the mRNA encoding this nascent peptide signal also functions as a recoding signal at the mRNA level in the mRNA exit channel and while it appears that the identity of the nts immediately 5′ of the shift site are important at the mRNA level, effects at the amino acid level may in addition be relevant (466). The Agaricomycetes are the only examples so far where bioinformatic analysis has pointed to a nascent peptide stimulator. However, we consider it likely that rather than Agaricomycetes being exceptional in that respect they may instead be exceptional in having reduced, or no, importance for the nucleotide sequence encoding a nascent peptide stimulator also having an important stimulatory role at the level of mRNA in the ribosomal mRNA channel. In this hypothesis, the counterpart nascent peptide sequence is also important in higher eukaryotes, but it is not apparent bioinformatically due to lack of third codon position variation because of the stimulatory action at the mRNA level also. Despite no pause being evident just 5′ of the shift site at the end of ORF1 in ribosome profiling experiments, perhaps because of sensitivity reasons, the hypothesis raises the issue whether prolines and glycines are encoded in a relevant position upstream of the antizyme frameshift site. Some interchangeability between the imino acid proline which is very different from glycine could be informative in relation to the distinction between potential nascent peptide decoding effects and those due to either product function outside the ribosome or nucleotide sequence effects during decoding.

Excluding the special case of antizyme 3, there is frequent occurrence of either a glycine or proline, as in human antizyme 1, encoded 7 codons 5′ of the shift site and flanked on one or other side by a proline or glycine (Table 2). The location of the proline in human antizyme 1 in a complex of antizyme and some of its interacting partners is now known (276,291). Because antizyme has other interacting partners, caution is needed about function in intact protein except to note that proline would be very different from glycine. However, though context dependent (see below) tandem proline or proline glycine (see below) can be slow-to-decode. Proline is an exceptionally poor donor and acceptor for peptide bond formation for steric rather than electronic reasons (467). Polyproline containing peptidyl-tRNA is prone to destabilization leading to drop-off (467), and the ribosomes are trapped in a pre-translocational state with a free E-site (468). In eukaryotes this is recognized by a protein with a tRNA-like shape, elongation factors eEF5 (formerly eIF5A), that aids polymerization, Reviews (469,470). Distinct from the type of association between polyamines and antizyme, in eEF5 a key lysine residue is uniquely post-translationally modified with polyamine-derived hypusine. Hypusine, and different modifications on the bacterial counterpart of eEF5, EF-P, likely evolved for the stabilization of the CCA-end of P-site tRNA (470,471) [Importance of CCA characteristics are shown by tRNA with CCA mutated to GCA or ACA causing frameshifting at a specific sequence and this being enhanced by mutants of *hrpA* (440,472) that encodes an RNA helicase (473). EF-P is also relevant to frame maintenance (474).] eEF5's hypusine moiety interacts with the backbone of the CCA-end of the P-site tRNA and entropically steers the substrate into a more favorable position in the peptidyl-transfer center (467,468,475). Reduced hypusination of eEF5 due to limiting polyamines, would likely lead to a greater stall presumably with increased drop-off (whether the third codon base in the key codons preferentially has non C or G in the third codon base to facilitate such hypothetical regulatory drop-off has not been addressed). Irrespective, perhaps reduced hypusination can influence the nascent peptide already in the peptide tunnel or some other feature of the ribosome that has an effect on frameshifting 6 codons later. Studies in bacteria have shown that the extent of stalling at proline codon stall sites is strongly influenced by the identity of the amino acids specified 5′ up to a distance of 3 to 5 codons, though the effect decreases with distance. Though the pattern is complex depending on the identity of the stall site, codons for H, K, Q, R or W enhance stalling in contrast to those for C, G, L, S or T (476,477) (Note in the StopGo mechanism, below, the flanking amino acid on the N-terminal side is N, and UGG encoding W is strongly conserved as the second codon 5′ of the antizyme shift site). Features of the nascent peptide just inside the peptide exit tunnel are likely relevant. The identity of the codon directly 3′ and the extent of spacing between ribosomes can also be relevant (476,478). [A progressive effect over several codons of the gene *60* bypassing nascent peptide signal is described 2 paragaraphs down.]

Sequence 5′ of the mammalian antizyme 1 shift site was shown to be important for the sensing of polyamine levels that influence frameshifting efficiency, though it was not distinguished whether the effect was direct or via its encoded product (465). However, in these experiments the polyamine levels were varied more drastically than in the experiments in *S. cerevisiae* and Agaricomycotina (268,466). Further work will be required to assess whether the mammalian cell findings in (465) reflect a basic system that was later refined for the sensing of subtler physiological variations in polyamine levels.

The codons directly 5′ of human antizyme 1 ORF1 stop specify NLGPGPRWCS (where S is encoded by the shift codon UCC, see Table 2 just above). This is similar to the sequence, NPGPVQS, directly 5′ of the tandem −1 frameshift shift site utilized in expression of Theiler's murine encephalomyelitis and Saffold cardioviruses (58,59). The first part of this sequence can be written NPG/P to signify that the G is the C-terminal amino acid of the upstream-encoded protein that is liberated by the action of StopGo. The P is the N-terminal amino acid of the protein whose synthesis involves frameshifting. [One of the names for the phenomenon of nascent peptide mediated release, without a stop codon, of upstream encoded protein and continued translation (without initiation) to specify a downstream encoded protein (479) is StopGo (480).] Whether the nascent peptide context required for StopGo serves to exclude eEF5 prior to facilitating hydrolysis of the ester linkage required for liberation of the upstream encoded protein is unknown. Under the conditions tested, which do not include altered polyamine levels, StopGo has not been shown to influence the frameshifting (58,59). However, whether naturally there is some influence of polyamine levels remains to be determined.

### Polyamine effects on other frameshifting

Despite its conservation, the stimulatory role for the Shine Dalgarno-like interaction by translating ribosomes on bacterial release factor 2 +1 frameshifting is deduced not to mask a role for the commonly encoded glycine since the sequence GG_GGG is sometimes GG_AGG which is also a good Shine Dalgarno type sequence but which does not encode glycine. Nevertheless, in the absence of a nascent peptide modulator and independent of mRNA:rRNA interaction stimulation, increasing polyamine levels, such as occur at the early logarithmic phase of growth, enhance release factor 2 frameshifting (481). While polyamines preferentially enhance the levels of quite a number of proteins whose synthesis does not involve frameshifting (481,482), this does not detract from significance of their stimulation of a subset of frameshifting, e.g. *E. coli dnaX* −1 frameshifting is not stimulated by polyamines (481). Ty1 +1 frameshifting can also be stimulated but it requires both elevated putrescine and greatly diminished spermidine (309). Considerations derived from these analyses are relevant to the point mentioned above about potential involvement of polyamines in type III secretion system frameshifting involving YopN-TyeA hybrid synthesis (331).

### Nascent peptide stimulators (continued)

The ICE introduced above that converts nonsymbiotic rhizobia into nitrogen fixing symbionts of leguminous plants utilizes +1 frameshifting in its expression. The 4th codon 5′ of the shift site UUU is a conserved tryptophan codon and it is flanked on its 3′ side by a glycine codon for which only the identity of the first 2 nts is conserved. Reasonably, it has been suggested that the encoded WG may influence the frameshifting (202). Though not noted earlier for the likely bypassing in decoding the terminase gene of *Streptomyces* phage Hau3 and *Streptomyces* prophage Strep C.1 (161), the codons 5′ adjacent to the key UUA codon specify KGWG, i.e. in this case glycines flank the tryptophan codon, and nascent peptide influence on the bypassing is likely.

Efficient bypassing of 50 non-coding nucleotides in decoding phage T4 gene *60*, is very dependent on a nascent peptide stimulator (160) that promotes the codon:anticodon dissociation that is a prelude to mRNA sliding during bypassing (451,483,484), and also affects the fidelity of coding resumption (483). The nascent peptide recoding signal causes progressively slower translation of the last five codons before the site in gene *60* mRNA of codon:anticodon dissociation. The bypassing step takes 10 to 20 times longer than a standard cycle step, and after coding resumption in the new frame, step times take several cycles to return to normal. The initial progressive slowing is very important for ribosomes assuming the non-canonical rotated state that is central to bypassing (451). Such progressive slowing is distinct from the prior prevailing thought that pausing sites in general would be single discreet sites. Unlike its *secM* counterpart the most important part of the gene *60* nascent peptide signal, KKYK, is 25 amino acids from the P-site when its effects on translational slowing starts to become apparent and 30 amino acids from the P-site at the time of codon:anticodon dissociation (451). Consistent with the number of amino acids from a key part of the nascent peptide signal to the P-site being important, while retaining the 14 gene *60* codons closest 5′ to the codon: anticodon site, replacing the further 5′ codons with *secM* sequence natively just 5′ of their pause site, still resulted in significant bypassing (451). However, some charge features of amino acids close to the P-site at the time of dissociation are also relevant (A. Coakley, N. Wills, P. O'Connor, G.L., J. Weissman, P.V.B. and J.F.A. in prep). At the time of nascent peptide mediated peptidyl-tRNA codon: anticodon dissociation there is a stop codon in the ribosomal A-site. Another stimulator of gene 60 bypassing is described next.

### Stimulators 5′ of ribosomal frameshift sites that act at the mRNA level

**Figure 11.**
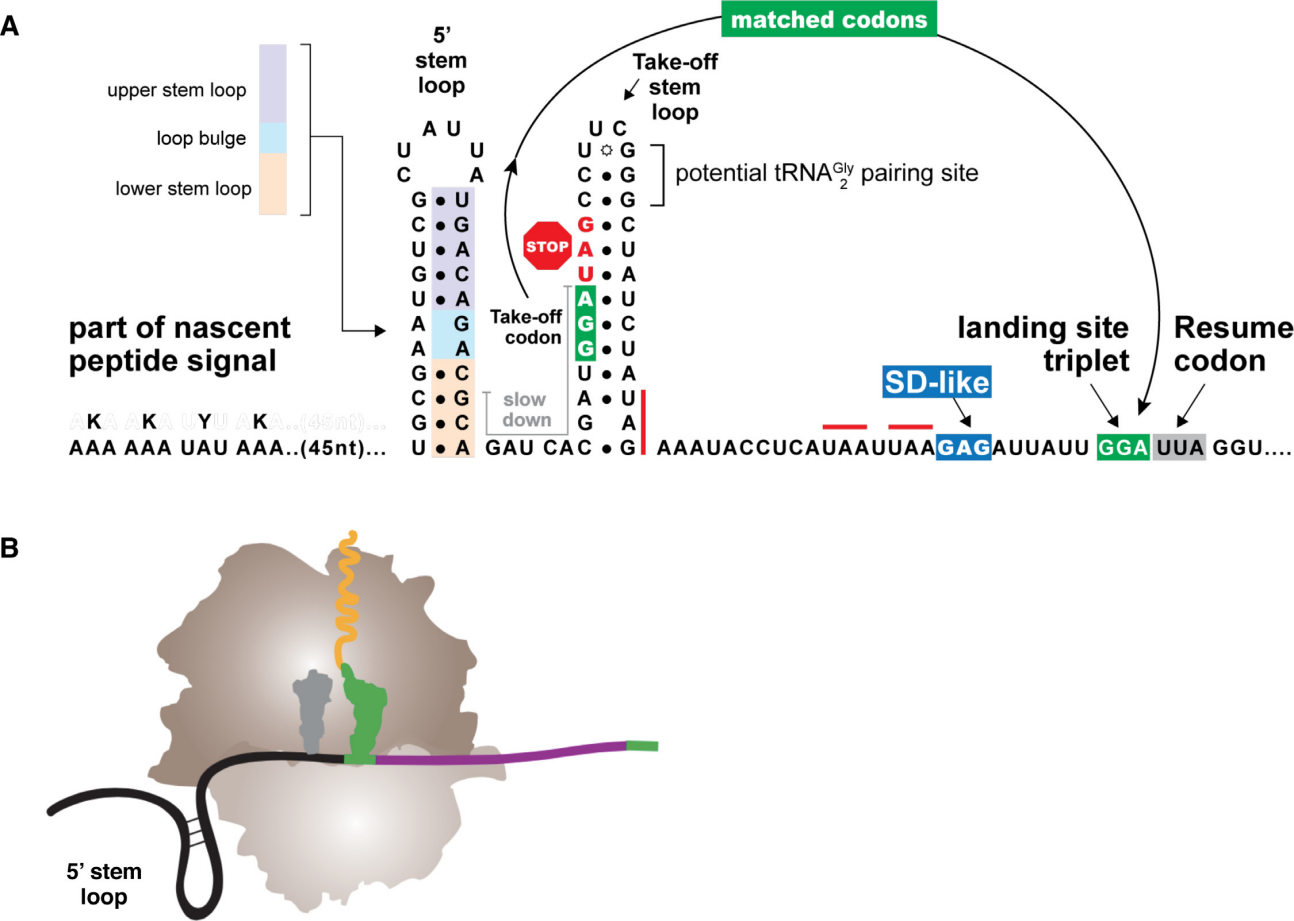
(**A**) Features important for translational bypassing in decoding T4 gene *60*. The matched take-off and landing codons, GGA, are shown in white letters in dark green boxes; the UAG stop codon immediately 3′ of the take-off site is in red letters next to the stop sign; stop codons within the coding gap are overlined in red; and sequences that slow ribosomes on approach to the second stem loop from the left, the take-off stem loop, are overlined in black. A Shine–Dalgarno-like sequence is shown in the blue box, and has a modest influence on landing site selection. The translational resume codon is indicated by the gray box. (**B**) Model depicting the predicted positioning of the 5′ stem loop and nascent peptide interaction when the GGA take-off codon is in the ribosomal P site. Part B Reproduced in part from Chen, J., Coakley, A., O'Connor, M., Petrov, A., O'Leary, S.E., Atkins, J.F. and Puglisi, J.D. (2015) Coupling of mRNA Structure Rearrangement to Ribosome Movement during Bypassing of Non-coding Regions. Cell, 163, 1267–1280 with permission from Elsevier.

Continuing with gene *60* bypassing: The take-off codon is paired to peptidyl-tRNA at the start of bypassing and so is unavailable for intra-mRNA pairing. Later, together with its flanking sequence, it forms a functionally important stem loop, designated ‘take-off stem loop’ (160,385) (Figure 11). Part of the sequence involved is 5′ of the take-off site. The time, or times, at which formation of the take-off stem loop is important remains to be determined – it has recently been discussed in detail (451). Sequence 5′ of that which becomes involved in the ‘take-off’ stem can form an independent stem loop, designated 5’ stem loop (485). Evidence is accumulating that by the time the take-off codon has reached the ribosomal P-site, the ‘top’ 5 bp of that stem have already paired just outside the ribosomal mRNA exit channel. The current model is that after liberation of the mRNA on P-site codon: anticodon dissociation, completion of pairing with 4 additional base pairs in the 5′ stem loop serves as a ‘motor’ by pulling mRNA 5′ to start bypassing movement (451,485) (A. Coakley, N. Wills, P. O'Connor, G.L., J. Weissman, P.V.B. and J.F.A. in prep). [Such mRNA structure formation mediated ribosome positioning has some parallels to the dicistrovirus InterGenic Region (IGR) IRES forming an extended structure that directs a proportion of the ribosomes on Israeli acute paralysis virus RNA to commence initiation in the +1 rather than the zero frame (181,486,487).]

Though the stop codon 3′ adjacent to the take-off codon in gene *60* mRNA is an important feature of bypassing at normal concentrations of release factor, it does not compete to mediate termination. The efficiency of take-off is very high (451,483,488). However, some ribosome drop-off does occur before retained ribosomes resume decoding (451,489). The anticodon of peptidyl-tRNA retained within the ribosome during bypassing does not scan at least the first half of the coding gap (385), but does appear to scan near the site of its re-pairing to mRNA at a matched GGA codon – the landing site (451). Pairing of a minimal internal Shine Dalgarno sequence 6 nts 5′ of the landing site with its rRNA complement in bypassing ribosomes has a modest effect on landing site selection (385) prior to coding resumption at the adjacent codon (a similar function for a Shine Dalgarno sequence has been proposed for the likely *Streptomyces* phage bypassing (161) though it is 5′ of the start of the much shorter coding gap).

**Figure 12.**
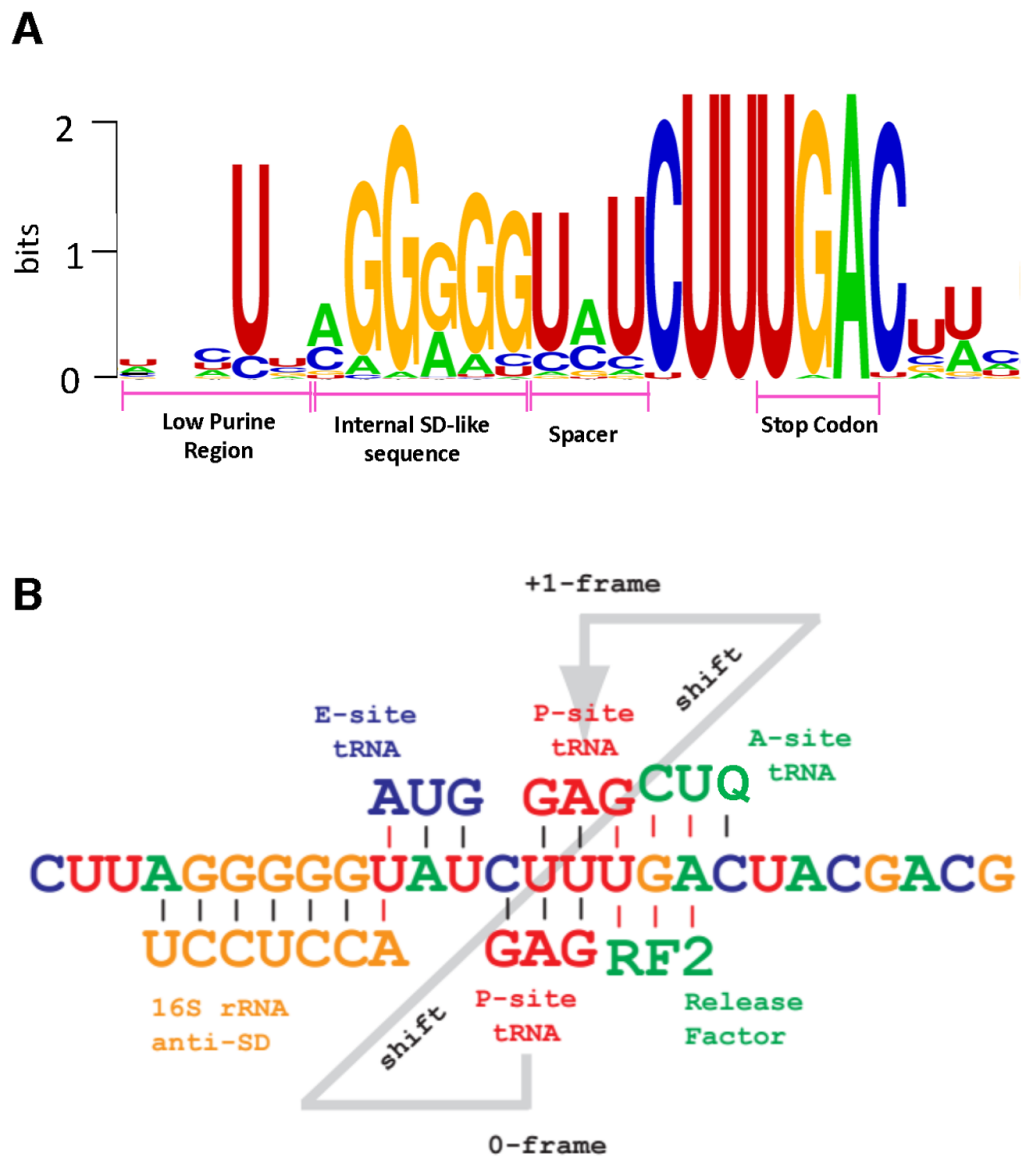
The features of release factor 2 autoregulatory frameshifting are highly conserved in diverse bacteria. The level of the release factor for the underlined ORF1 stop codon UGA(C) governs the efficiency with which the anticodon paired to the 5′ adjacent codon, CUU, shifts +1 to pair with UUU permitting continued translation to synthesize release factor 2. The frameshift stimulatory internal Shine Dalgarno (SD) sequence is preceeded by sequence unable to pair with the anti-Shine Dalgarno sequence of translating ribosomes, permitting availability of the anti-Shine Dalgarno to form the correctly positioned frameshift stimulatory hybrid with mRNA. The figure was generated with Weblogo3 (815) using RF2 frameshift sites obtained from ARFA (348).

While the role of the Shine Dalgarno sequence in this case is relatively unimportant, internal Shine Dalgarno-like sequences are of major importance for bacterial single nucleotide frameshifting both −1 and +1. The number of nucleotides between frameshift stimulatory Shine Dalgarno sequences and the shift site at which it exerts its effect, depends on the directionality of the shift. For +1 frameshifting, it is 3 nts (54,490) and for −1 frameshifting it is 9–14 nts (491). It was known since 1975 (492), that 30S ribosomal subunit recognition of Shine Dalgano sequences to position initiation was due to pairing of a sequnce near the 3′ end of 16S rRNA. However, it was not suspected that this ‘anti-Shine Dalgarno’ sequence in translating 70S ribosomes scanned mRNA being decoding for potential complementarity. The finding of Shine Dalgarno-like stimulators for frameshifting showed that such scanning must occur (54,493). [It has also been recently shown for threading-mediated 70S-scanning initiation (494), and is also likely important for translational coupling where the Shine Dalgarno sequence for initiation of a 3′ adjacent gene is before the terminator of the 5′ gene.] The −1 frameshifting assays led to the deduction that, after formation, the hybrid stays intact as the ribosome translates 5–7 nts and only then does it rupture (491). Consistent with this, and the need for the anti-Shine Dalgano to be available to pair with its frameshift stimulatory complement and not be sequestered by pairing to a 5′ counterpart, the sequence directly 5′ of the stimulatory Shine Dalgarno lacks potential competitor Shine Dalgarno sequences (394) (Figure 12). Direct supportive evidence for hybrid maintenance for several translation cycles comes from the extended number of nucleotides protected from ribonuclease digestion, at internal Shine Dalgarno containing sites revealed by a ribosome profiling/footprinting study (495).

A 5′ Shine Dalgarno stimulator is a major feature of the autoregulatory +1 frameshifting in bacterial release factor 2 synthesis (54,443,493,496) and as illustrated (Figure 12), it is highly conserved. It is also a major feature of the −1 frameshifting in the expression of *E. coli dnaX* (491) and an estimated 56% of IS elements (187).

There is a Shine Dalgarno sequence just before the end of many genes in bacterial and phage polycistronic mRNAs. Potentially its pairing with the anti-Shine Dalgarno sequence of ribosomes approaching the termination codon, could serve to retain at least the 30S subunit to restart without diffusion when the stop and start codons overlap or are otherwise close together on translationally coupled genes.

One Shine Dalgarno sequence serves as both an initiator and frameshift stimulator in an IS element (194). Mechanistic studies of their action are described in a separate section below.

Though eukaryotes do not have Shine Dalgarno interactions, an mRNA sequence 5′ of the shift site in many antizyme genes acts directly to stimulate antizyme frameshifting (264,270). There is substantial evidence that it acts at the mRNA level without intra-mRNA pairing, though cryoEM information would be invaluable. Independent from this, there is suggestive evidence for mRNA: rRNA pairing within 80S ribosomes in some cases of initiation/reinitiation (497–500) and shunting (501,502).

A stem loop structure 5′ of the barley yellow dwarf virus shift site was found to have a 50% effect on *in vivo* frameshifting and is conserved in all members of the *Luteovirus* and *Dianthovirus* genera (110). These upstream stem loop(s) were suggested to either slow the ribosome in advance of the shifty site to enhance frameshifting, or serve as ‘insulators’ to prevent improper folding of the shifty site or 3′ long range structural element with upstream sequences (110). This finding emerged close to the time of an early report of sequence immediately 5′ of the HIV-1 and HTLV-2 shifty sites influencing frameshifting efficiency (503). The stem loop structure noted above that is 5′ of the gene *60* take-off site region and important for bypassing, has been proposed to propel ribosomes through the 5′ segment of the coding gap. It is explicable by an mRNA zippering effect as mRNA emerges from the mRNA exit channel (451,485).

### A 5′ inhibitor for −1 ribosomal frameshifting can be a stimulator for +1 frameshifting

Recently a natural stem loop structure 4 nts 5′ of the SARS coronavirus −1 frameshift site was reported to attenuate the −1 frameshifting level involved but to be able to stimulate +1 frameshifting in tests with synthetic constructs (504). It is mimicable by a paired antisense oligo (505). However, its functioning is still mysterious. An inhibitory stem loop has also been reported for pea enation mosaic virus frameshifting (406). Earlier the effect of inserting a palindromic sequence that could form a stem loop structure was tested on feline immunodeficiency virus frameshifting (506). To what extent inhibitors serve to link ribosome loading to frameshifting efficiency, and for virus infection potentially with phase of the infective cycle, remains to be seen.

**Figure 13.**
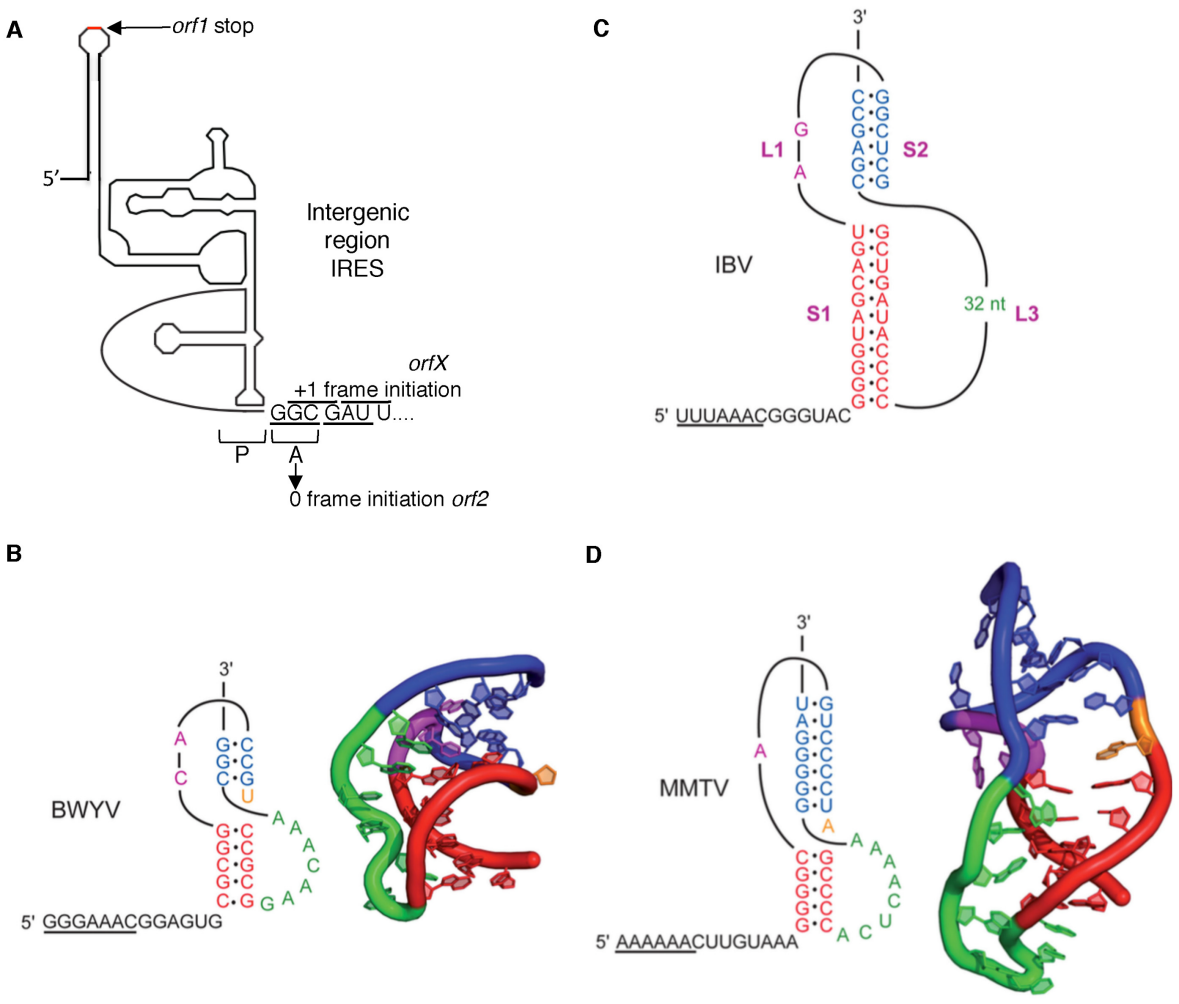
2D and 3D models of RNA structures that promote access to alternative reading frames in viral genes. (**A**) The intergenic Internal Ribosome Entry Site (IRES) of the dicistrovirus, Israeli acute paralysis virus, causes most ribosomes to initiate 1 base 5′ of where the others initiate. Stimulators for ribosomal frameshifting are shown for (**B**) beet western yellows virus (BWYV), (**C**) infectious bronchitis virus (IBV) and (**D**) mouse mammary tumor virus *gag/pro* (MMTV). Slippery sequences are underlined. Reproduced in part from Brierley, I., Gilbert, R.J.C. and Pennell, S., Pseudoknot-Dependent Programmed -1 Ribosomal Frameshifting: Structures, Mechanisms and Models (2010) In: Atkins, J.F. and Gesteland, R.F. (eds.), Recoding: Expansion of Decoding Rules Enriches Gene Expression. Springer New York, New York, pp. 149–174, with permission from Springer.

Alternative conformations of a 5′ intra-mRNA structure dictating which of two overlapping codons is used for initiation and so framing is of course, very different, though some parallels justify inclusion here of the dicistrovirus case introduced above (Figure 13A).

### ‘Spacers’

The word ‘spacer’ is most commonly used to describe the nt distance between the shift site and a 3′ structural stimulator, but is more generally used to signify the distance between a recoding signal and the site at which a non-standard event occurs, e.g. between a 5′ stimulatory Shine Dalgarno sequence and the shift site. However, especially for nucleotides between the shift site and a recognized 3′ stimulator, ‘spacer’ nt identity has to be treated carefully since it may be significant (405). This has been explored systematically in *E. coli* (192) with attention being paid to stacking potential of spacer nucleotides. That study used an A_AAA_AAG shift site. Independent of utilization for frameshifting, AAA is used ∼3 times more commonly than AAG except when the 3′ adjacent base is C when AAG is preferred (507). However, in the frameshifting study though 3′ adjacent CC was the most shift-prone of the dincleotides, 3′ C was in general more shift-prone than U (192). Spacer nucleotide effects has also been studied with a variety of cassettes to a lesser extent in eukaryotes (104,508) where in some cases an initially unrecognized ‘lower’ part of an identified structure, was thought to be ‘spacer’ nts, and, as just described, potential to interact directly with ribosomal components is a comparable issue.

Many studies have focused on −1 frameshifting at heptanucleotide shift sites with a spacer distance of 5–9 nts from the 3′ end of the shift heptanucleotide to the 5′ end of a 3′ intra-mRNA structural stimulator thought to be at the unwinding site of the mRNA entrance channel at the start of frameshifting. Associated with this has been an under-appreciation of the variety of spacer lengths. As discussed in other sections these range from 3 nts for mammalian antizymes 1 and 2 +1 frameshifting to the 11–14 nts for arterivirus −2 and cardiovirus frameshifting. (Relevance of spacer length to shift directionality is dealt with in the next section.)

### Intra-mRNA pairing stimulators for ribosomal frameshifting – much ado about knotting

**Figure 14.**
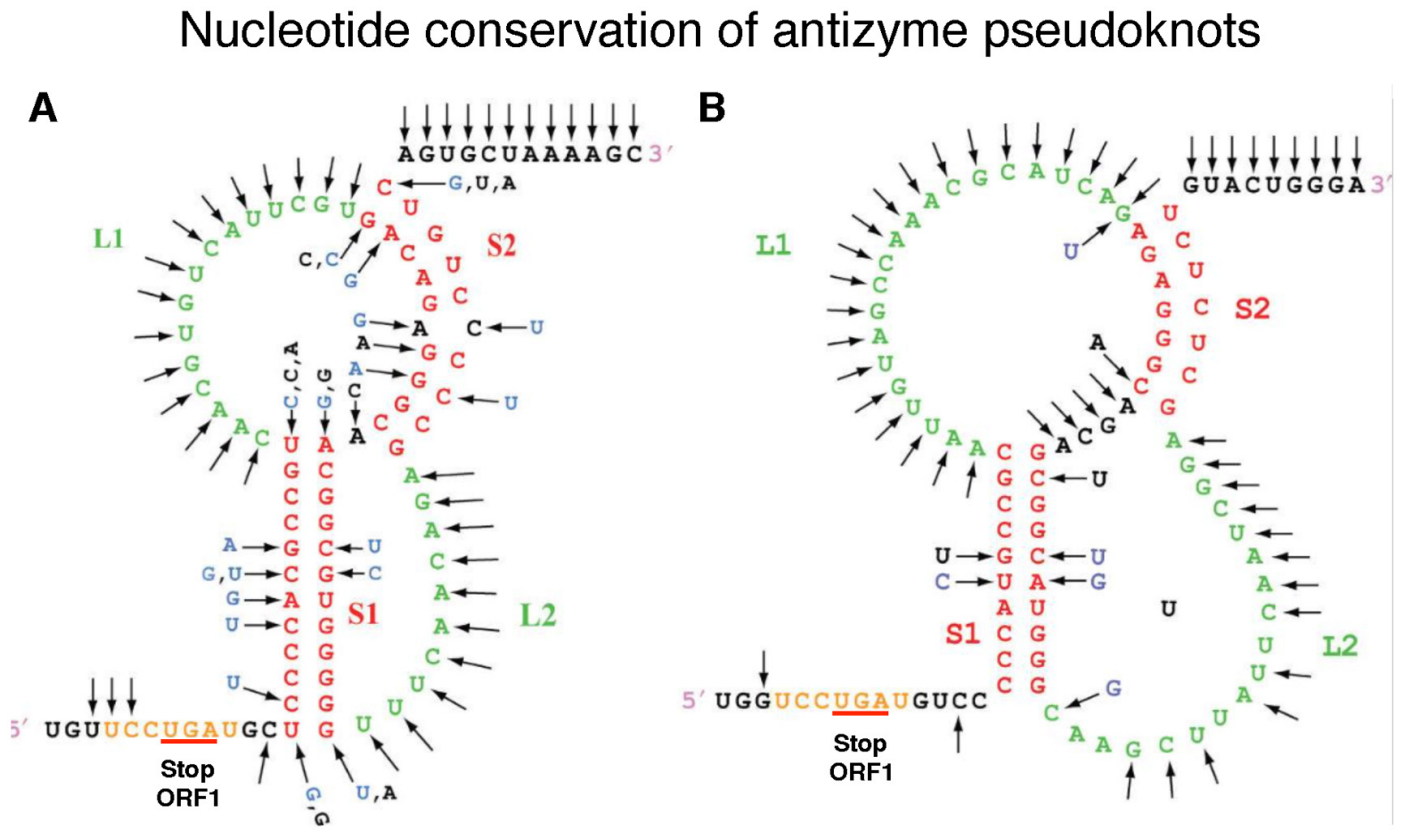
Sequence variability of antizyme frameshift stimulatory pseudoknot single-stranded regions contrasts with that involved in stem formation. The illustration is from pseudoknots from invertebrates (an oyster and an aphid) that differ from their mammalian counterparts. (**A**) *Crassostrea gigas*, (**B**) An aphid. The frameshift site is indicated with orange letters. Black arrowheads represent substitutions deduced from phylogenetic comparison to orthologous genes. Non-compensatory changes in the stems are shown in black letters; compensatory changes are shown in blue letters.

HIV also illustrates pairing of sequence just 5′ of the shift site with sequence 3′ of the shift site to form a structural base ‘anchoring helix’ that facilitates a functionally important transition between two alternate forms of the upper part of the structure mostly specified 3′ of the shift site (509). Other recent HIV experiments are also relevant (135,510). Despite this case nearly all 3′ recoding stimulators known are specified wholly 3′ of the shift site, even though in a few cases their extreme proximity to the shift site, just 3 nts for mammalian antizyme 1 +1 frameshifting, would require at least unwinding of the ‘lower’ part of the structure before the shift site is in the ribosomal P-/A- sites. Whether the important feature is a ‘set up’ for the remaining part of the structure or an alternative considered in the nascent peptide section above, is unknown. Irrespective of whether the antizyme pseudoknot stimulates the shift directly or indirectly, the extent of conservation of its constituent stems, but not its unpaired components, illustrates the value of what can be termed ‘phylogenetic probing’ for structure function inferences (270) (Figure 14). The diversity of modulators 5′ of the shift site is at least matched by that of 3′ stimulators. As mentioned, the majority of intra-mRNA 3′ stimulatory signals, have their 5′ ends 6–9 nts 3′ of the shift site and are thought to be at the mRNA unwinding site within the mRNA entrance tunnel (511,512) when the shift site is in the P-/A- sites. Expression in heterologous systems (513,514) and in homologous systems (98,406,515), have revealed differences between bacteria and eukaryotic systems in optimal spacer lengths or within eukaryotes for specific 3′ stimulators, in one case with implications for frameshift directionality. For a minority of 3′ stimulators their 5′ ends are 12–15 nts 3′ of the shift site and are likely to have their influence at the outer edge of the mRNA entrance channel. These stimulators can involve either intra-mRNA structure or ‘linear’ mRNA with the characterized ones being associated with *trans*-acting factors (23,58,59).

While the effectiveness of stimulators in general is selected to act at different efficiencies in different cases, the upper ‘strength’ limit for 3′ intra-mRNA structural stimulations is that they must not act as a complete ‘roadblock’ to ribosome progression (516). The type of 3′ structures range from simple stem loops, as with *E. coli dnaX* −1 frameshifting (392,403,517,518), likely plant sobemoviruses (463,519–521) and explored systematically with the simian retrovirus shift site (522), to more elaborate stem loop structures such as in bacterial IS911 (188) and HIV (509,510) to compact pseudoknots as in poleroviruses, e.g. beet western yellows virus (Figure 13B), potato leaf roll virus and sugarcane yellow leaf virus (523,524), larger pseudoknots with all components specified nearby (see below), to pseudoknots in which a lateral bulge-loop of an extended stem-loop structure 3′-adjacent to the frameshift site interacts with an apical loop of a distant stem-loop structure (ALIL) (45,110,525). A subset of related sequences can use either stem loop or pseudoknot stimulators, for instance among IS elements (187), different alphaviruses (140), and probably different flaviviruses (70,120,123,124). The great majority of the frameshift stimulatory pseudoknots studied are for −1 frameshifting, but some are known for +1 frameshifting. Multiple features of 3′ intra-mRNA structural stimulators may influence frameshifting including thermodynamic stability, stem and loop lengths, torsional resistance, susceptibility or not to a protonated dependent switch and specific three-dimensional structures.

The involvement of pseudoknots as frameshift stimulators was first shown for the coronavirus, infectious bronchitis virus (526) (Figure 13C) and predicted to be widespread (527,528) ∼4 years after the discovery of retroviral frameshifting. The great majority are H (hairpin)-type pseudoknots meaning that a sequence of nucleotides within a hairpin loop base-pairs with a complementary region external to the hairpin. All H-type pseudoknots contain two helical stems, S1 and S2, and two non-equivalent loops, L1 and L2. Some also contain a third loop, L3. The IBV frameshift stimulatory pseudoknot features a long stem 1 which cannot be functionally reduced to less than 11 base pairs, significantly equivalent to one turn of an RNA A-form helix (106), and other pseudoknots such as those in the alphavirus, western equine encephalitis virus also have long stems (140). In contrast, the MMTV *gag-pro* frameshift stimulatory pseudoknot (529), features an unpaired intercalated ‘wedge’ adenosine between two short stems of similar length (Figure 13D). This frameshift stimulatory pseudoknot was the first to be characterized by NMR (530). Thermodynamic and structural insights into the role divalent metal ions play in stabilizing the structure followed (531,532). The intercalated, or ‘wedge’ A gives a bent, ‘kinked’, structure that is important for function (533,534), though it contributes only modestly to stability (532). A wedge A base is also present in several other frameshift stimulatory pseudoknots (85,86,227) including the related simian retrovirus-1 *gag-pro* pseudoknot (535,536) where, despite the wedge A, NMR analysis highlighted the importance of stem coaxial stacking and loop L2-stem S1 interactions (537). Other studies focused on the importance of the triple helical feature and several interactions (538,539).

Highlighting the contrast between the IBV and MMTV *gag-pro* type pseudoknots just considered, a functionally inactive mutant of the former with 6 bps in stem 1 and 8 nts in loop 2 could be converted to the counterpart of an active MMTV *gag-pro* pseudoknot by addition of an unpaired and presumably intercalated adenosine at the helical junction, and an adenosine in the 3′ terminal position of loop 2 (540). The efficiency of the infectious bronchitis virus pseudoknot with its long stem 1, is unrelated to the thermodynamic stability (106) or its mechanical unwinding properties (541). The first cryoEM studies of ribosomal response to a frameshift stimulatory pseudoknot utilized the avian infectious bronchitis virus pseudoknot and revealed distorted tRNAs in two different A/P hybrid states (108,542). The more recent major improvements in cryoEM sensitivity means that invaluable insights will be forthcoming.

A number of other coronaviruses utilize a different type of pseudoknot termed ‘elaborated pseudoknot’ or ‘kissing stem loops’. Initially found for human coronavirus 229E (109), it is also utilized by viruses such as transmissible gastroenteritis virus and porcine epidemic diarrhea virus (543). The pairing involves apical loops, and counterpart pairing involving an internal loop on one or other of the stems is described in the next paragraph. In contrast, the pseudoknot of the coronavirus that mediates SARS disease is an H-type pseudoknot, like that of infectious bronchitis virus. However, that of SARS virus differs in having part of its counterpart of loop 3 folded into a stem loop (543–546). This is involved in intermolecular kissing loop: loop pairing that functions in viral genomic RNA dimerization that in turn affects frameshift efficiency with likely regulatory consequences (547). A pseudoknot stem 3 is also known to be significant for Rous sarcoma virus *gag-pol* frameshifting (548,549). An additional sub-structure between the main stem loops known as an ‘Interstem Element’ was characterized in the Visna–Maedi retrovirus frameshift stimulatory pseudoknot where it unusually comprises a 7-nt loop between the two stems and is essential for frameshifting promotion (550). This pseudoknot features a long loop 1 (550). By extrapolation an interstem element of 3 nts also occurs in the pseudoknot of the 1a/1b frameshift stimulator of the arterivirus lactate dehydrogenase-elevating virus (550,551).

For the luteovirus, barley yellow dwarf virus, a 6-nt internal loop on the 3′ side of a stem loop 6 nt 3′ of the shift site pairs with the apical loop of a stem loop 4 kb 3′ of the shift site (110,552,553). A similar arrangement, involving what is termed an ALIL pseudoknot, was also predicted for viruses in two *Tombusviridae* genera, *Dianthovirus* and *Umbravirus* (406,463). This was experimentally demonstrated for a member of each, red clover necrotic mosaic virus (554) and the RNA 2 (406) of pea enation mosaic virus (whose taxonomically unrelated RNA 1 is dealt with below). For RNA 2, the long-distance interaction modifies the lower stem of the stimulatory structure close 3′ to the shift site, perhaps by strengthening its stability. However, the same study left open possible relevance of the interaction bringing close other sequence near the 3′ end (406). It is unknown if it is relevant that RNA 2, like the RNAs of many positive-strand RNA viruses, lacks a 5′ cap structure and its cap-independent translation features a sequence in the 3′UTR binding eIF4E and so recruiting eIF4F to mediate initiation via a long distance interaction (555). (In barley yellow dwarf virus, the distal apical loop involved in long distance pairing to the frameshift stimulatory structure just 3′ of the shift site is about 200 nts 3′ of the element that permits cap-independent initiation.) Regardless, quite different considerations apply for the structure involved in bacterial IS3411 ribosomal frameshifting, and at least 27 other IS elements (45). The counterpart involves pairing of the apical loop of a similarly positioned stem 1 with an internal loop of a stem loop 2 that is positioned in the range of 44 to 104 nts 3′ depending on the IS element (45). Remarkably the second component can, to some extent, act *in trans* (45). With only two variant types currently known, others remain to be found. In addition, some unrelated long range effects remain to be explained including for phage T7 gene *10* frameshifting, where a CCCC sequence in the transcription terminator over 200 nts 3′ of the shift site is involved (397).

Elaborate structures do not just involve pseudoknots. The bacterial transposable element IS911 stimulatory structure has a three-way junction and constituent stem loops (188,194). The 3′ stem loop important for *H. neapolitans* carboxysome *cso2* frameshifting is more complex (316) than its *dnaX* counterpart (517), but there appears to be no 5′ Shine Dalgarno stimulator in that case. (Just 2 substitutions in the *E. coli dnaX* stem loop can increase frameshifting to 176% of the WT level, so selection can readily yield the optimum efficiency.) While most work on bacterial mRNA structural stimulators has been done with stem loops, pseudoknots are also employed for productive frameshifting by bacterial ribosomes both −1 (146,399) and +1 (153,154).

The frameshift stimulatory pseudoknot of the poleroviruses, beet western yellows virus (556) (Figure 13B), which is similar to that of its relative potato leafroll virus (557), was the first frameshift promoting structure to be determined at atomic level and is remarkably compact (558). In addition to species of the genera *Luteovirus* and *Polerovirus*, the *Luteoviridae* family of viruses also contains the genus *Enamovirus* of which RNA-1 of pea enation mosaic virus is a member. The frameshift stimulatory pseudoknot of this RNA-1 has remarkable features including extensive triple-strandedness (559,560). The sugarcane yellow leaf virus frameshift stimulatory pseudoknot, which is similar to its beet western yellows virus counterpart, also has compact features that involve extensive non-Watson–Crick pairing and a base quadruple. It has an extended triplex between the minor groove of stem 1 and loop 3 involving interactions that have features in common with A-minor interactions (523,561). In this class of pseudoknots, the lowest energy ‘ground state’ does not correlate with frameshifting efficiency, but rather an enhancing of certain reduced stability (463,560). It exhibits pH-dependent, frameshift activity (523), that by analogy to the structural switch revealed by an NMR study of the pseudoknot stimulator of Moloney murine leukemia virus *gag* readthrough, is very likely due to a protonation dependent switch that induces the active frameshift stimulator (562).

The 3′ frameshifting stimulator closest to the shift site in the RNA 2, and umbravirus, component, of pea enation mosaic virus likely also undergoes important conformational state switching (562). The binding of an internal loop of this structure (3rd last paragraph above) to a 3′ distant apical loop may well be related to this.

Studies of HIV recoding signal conformational changes were introduced at the start of this section because of the evidence for relevance of sequence both 5′ and 3′ of the shift site. Distinct from the techniques employed in its analysis, optical trapping has been used to reveal a dynamic ensemble of conformations of the mRNA encoding CCR5, a coreceptor for HIV-1, but their relevance to frameshifting requires further work (563). However, it joins optical tweezers and others in the arsenal of techniques available. In contrast to several earlier proposals, optical tweezers experiments have strongly pointed to conformational dynamics (plasticity) being of prime relevance to frameshifting efficiency rather than the degree of pseudoknot resistance to mechanical unfolding (564). Consistent with this, not all stable pseudoknots stimulate frameshifting and both structural (88,565,566) and mutational analysis of those that do, and do not, promote frameshifting has been insightful. There are many analyses of both categories but one of the mutational analyses is of the compact stimulatory pseudoknots of beet western yellows virus and potato leafroll virus where evidence has been obtained for certain bases being functionally significant but lacking substantial stability effects (567). Studies with two pseudoknot stimulators show that frameshift efficiency is sensitive to the reading phase in which the translating ribosome encounters the pseudoknot but is not simply correlated with the extent of the associated translational pause (107).

Many inferences have been made about the mechanism of recoding signal action, especially 3′ mRNA structural stimulators where resistance to unwinding (511), is highly relevant (108,568). Though mostly considered as tension in mRNA, it can alternatively be considered as the transitory impediment to standard translocation resulting in tension for the anticodon: codon pairing of ribosome bound tRNA. Spacer length is relevant to this. With the HIV frameshift cassette, when the number of nts between the shift site is reduced, the ratio of −2 to −1 frameshifting increases (98). While a mammalian antizyme 1 frameshifting cassette yields +1 frameshifting in mammalian cells (264) and mostly +1 in the fission yeast *S. pombe* (569), it yields predominately −2 frameshifting in budding yeast *S. cerevisiae*, except when the 3′ pseudoknot is moved 3 nts further 3′ when the frameshifting is 50% −2 and 50% +1 (513).

In bacteria, stem loop structures whose 5′ end is within 15 nts 3′ of a stop codon, are frequent in many stress-related genes. However, as yet, there is no hint of any of these acting to promote frameshifting. (In these cases, and also after other slow-to-decode codons, tmRNA action is triggered with resultant mRNA decay (570), but possible effects of tmRNA on occurrences of frameshifting have only been tested to a very limited extent so far.)

In studies with synthetic constructs G-quadruplexes have been shown to also stimulate frameshifting (571,572). However, natural usage of G-quadruplexes to stimulate productive access to new reading frames either by affecting transcription or translation or both, is not currently known but is anticipated.

**Figure 15.**
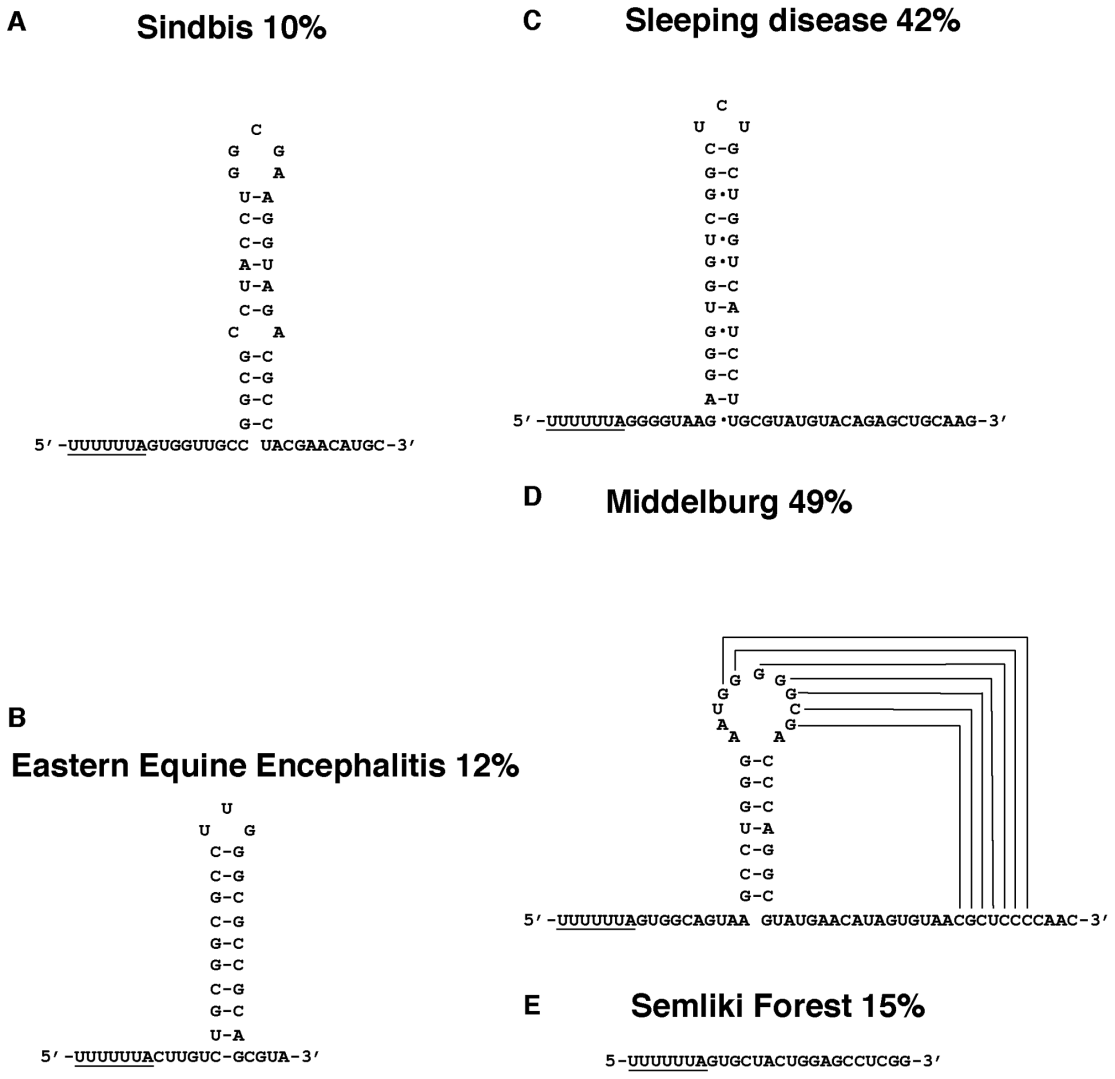
Alphavirus ribosomal frameshift stimulators are diverse. A single stem loop is the frameshifting stimulator for (**A**) Sindbis virus, (**B**) eastern equine encephalitis virus, (**C**) sleeping disease virus, and (**D**) Middelburg virus. Experimental support has been obtained for the second stem of the pseudoknot shown. (**E**) The stimulator for Semliki Forest virus frameshifting does not appear to involve intra-mRNA pairing, and is a candidate for exerting its effect via pairing with rRNA in the mRNA entrance channel. A similarly acting 3′ sequence may be acting as well as the pseudoknot, to stimulate the efficiency of frameshifting in Middelburg virus genome decoding to the high level of 49%. Reproduced from Chung B.Y.W., Firth A.E., Atkins J.F. (2010) Frameshifting in Alphaviruses: A Diversity of 3′ Stimulatory Structures, Journal of Molecular Biology, 397, 448–456 with permission from Elsevier.

While this section has illustrated the wide variety of intra-mRNA structural frameshift stimulators, a reminder of specificity comes from the numbers of modest mutational changes that greatly reduce frameshift enhancement. Further, an illustration of the potential for an effect of mutations very close to a 3′ structural stimulator not necessarily reflecting the structure directly but perhaps an inter-relationship of 3′ stimulators comes from a study of the alphavirus, Middelburg virus, that uses a 3′ pseudoknot frameshift stimulator (potentially with a 7-bp stem 2). This analysis was provoked by the study described at the start of the next section below, on a different alphavirus (as mentioned above the frameshifting in different alphaviruses uses diverse stimulators (137,140)) (Figure 15).

### 3′ stimulators for ribosomal frameshifting that do not act via intra-mRNA pairing

Alphaviruses continued: Though the sequence 3′ of the shift site in the chikungunya-related virus, Semliki Forest virus can be drawn as a weak stem loop structure, mutagenic analysis showed no evidence for a relevant functional 3′ secondary structure. Instead it was suggested that within the ribosome's mRNA entrance channel, it may act by pairing to rRNA (140). The sequence between the shift site and the 3′ pseudoknot frameshift stimulator in Middelburg virus, that in other circumstances would be called a spacer, is similar to that of Semliki Forest virus. Indeed mutating this sequence caused a drastic reduction in frameshift efficiency (140). Further work is needed to ascertain if Middelburg virus frameshifting involves both pairing to rRNA and a pseudoknot.

The 3′ stimulator for *S. cerevisiae* Ty3 +1 frameshifting identified earlier is also thought to act without intra-mRNA pairing in the ribosome mRNA entrance channel. A functional model involving non-base pair interactions was considered (214,215). This 14-nt sequence has a 7.5-fold stimulatory effect on Ty3 frameshifting (214). A conserved 3′ stimulator with different sequence but similar effectiveness for *EST3* +1 frameshifting has also been mutagenically characterized, and is almost twice as long (241). While the EST3 and Ty3 3′ stimulators can act on both the Ty1 and Ty3 shift sites, interestingly, both are ineffective with a stop codon instead of a slow-to-decode sense codon in the A-site (as in the *S. cerevisiae* antizyme site GCG_UGA_C) (241,421).

Porcine reproductive and respiratory syndrome virus (family *Arteriviridae*) frameshifting (22) is trans-activated by a protein complex composed of a virally-encoded replicase subunit, nsp1β and a cellular poly(C) binding protein interacting with the mRNA sequence CCCANCUCC 11 nucleotides 3′ of the shift site, and so the 3′ stimulator potentially permits temporal control during the course of viral infection (23; S. Napthine, E.Treffers, S. Bell, I. Goodfellow, Y. Fang, A.E.F., E. Snijder and I. Brierley, submitted). Binding of the complex mimics the effect of a structured mRNA frameshift stimulator. Earlier work showed that nsp1β interacts with a ribosomal protein, RpS14 (573). This protein is adjacent to RpS3 which is thought to function in mRNA unwinding activity (512).

A completely different type of 3′ signal influences *S. cerevisiae* antizyme +1 regulatory frameshifting (268). At low polyamine levels when synthesis of the negative regulator antizyme is not beneficial, the interaction of a specific nascent peptide sequence encoded far downstream of the frameshift site with the ribosome exit tunnel leads to a stall. This stall is thought to block the transit of following ribosomes with a trail-back effect on frameshifting. However, at high polyamine levels, the polyamines are proposed to interact with the downstream-encoded nascent peptide while within the ribosome and prevent formation of the stalling interaction. Then absence of a ribosome pile-up is proposed to allow the intrinsically high level of frameshifting to occur (268).

### Stimulator effects on ribosome excursions. Classical pausing studies and mutants of other potentially relevant components

After peptidyl transfer, the ribosome becomes ‘unlocked’ as small subunit counterclockwise rotation, coupled with backwards tilting, facilitates temporary withdrawal of rRNA ‘codon gating’ and mRNA: tRNA translocation across the ribosomal sites. Ribosome-tRNA and tRNA-mRNA interactions are weaker at that stage before clockwise rotation back and relocking (574,575). Even in standard decoding there is fluctuation in the unlocked state (576–578). Uncoupling of translocation from back rotation and formation of some non-canonical state, either intermediate or hyper-rotated, is emerging as relevant to a well-studied case of bacterial frameshifting.

Single molecule FRET and small angle X-ray scattering experiments on the *E. coli dnaX* shift cassette (403,518,579), optical tweezer experiments and detailed product characterization (392) and parallel ensemble FRET kinetic experiments on a variant of the infectious bronchitis virus frameshift cassette (450), have greatly refined knowledge of −1 frameshifting, part of which will be discussed after some comments on the systems used (also see (580)). One of the smFRET experiments used a strengthened variant of the *dnaX* stem loop structure with a G inserted opposite a bulged C, and the A of an A:G apposition changed to a C (518), and the other used a stem with the same number of base pairs without any unpaired bases, though with a different sequence (403). The single ribosome trajectory experiments studied by optical tweezers, and the associated mass spectrometric analayses of products were performed with mRNAs with potential to form a stem of either 25 or 55 base pairs (392), instead of the 11 base pairs (including an A:U at the base of the stem) found to be functionally relevant *in vivo* (517). The calculated strength of variant *dnaX* stem loop structures correlates directly with frameshift efficiency (517) and even without the 5′ Shine Dalgarno stimulator just substituting one base in the otherwise WT stem to allow uninterrupted pairing, causes 160% of WT frameshifting (80% of ribosomes shift frame). While further strengthening the stem may facilitate molecular tweezer experiments, it may also approach the ‘roadblocking’ effect seen with extra strong pseudoknots, especially given the expected interchangeability of simple stem loops and pseudoknots. Accordingly, any extrapolations from the finding of −4 frameshifting in the studies of (392) to biological relevance needs to be treated with caution. The ensemble FRET experiments (450) used a shift site, U_UUA_AAG, that is different from that of IBV, U_UUA_AAC and of *dnaX*, A_AAA_AAG and the identity of the different P-site tRNAs influences the type of shift occurring (395). [The whole pseudoknot or just its stem 1, can function in such heterologous expression systems (395,581) though with differing effectiveness.] It is unknown to what extent these, or other differences in the systems used, account for the discrepancies reported. One set of the *dnaX* results is interpreted to imply uncoupling of EF-G catalyzed translocation from standard ribosomal reverse rotation, leaving the ribosomes in a non-canonical rotated state during which the incoming aminoacyl-tRNA has potential to pair in the 0 or −1 frame (403) (see also (108)). In contrast, the other papers advocate slippage during tRNA–mRNA translocation at the second codon of the shift site (392,450,518). Also relevant to these studies is the distance between the initiating Shine Dalgarno sequence and the internal frameshift stimulatory Shine Dalgarno sequence since pairing is maintained for translocation over several nts and adequate space has to be present to prevent interference (394,491,495).

These studies have revealed a hyper-rotated ribosomal state that has widespread implications, and have vastly improved knowledge of the elongation pausing compared to non-frameshifted ribosomes. In one of the studies the overall pause was determined to be 10-fold longer (403) and in another EF-G was found to dissociate >15 times more slowly than from a sequence lacking a frameshift consensus sequence (450). The stimulatory role of the ribosomal RNA of the translating ribosome pairing with the internal Shine Dalgargo sequence 10 nts 5′ of the shift site has been clarified and found to cause large scale back and forth movements of the ribosome ≥1 codon on average specifically around the shift site region (392,403,518). These stochastic translocation attempts are not seen in its absence, i.e. the 3′ stem loop or the shift sequence alone does not cause such fluctuation.

**Figure 16.**
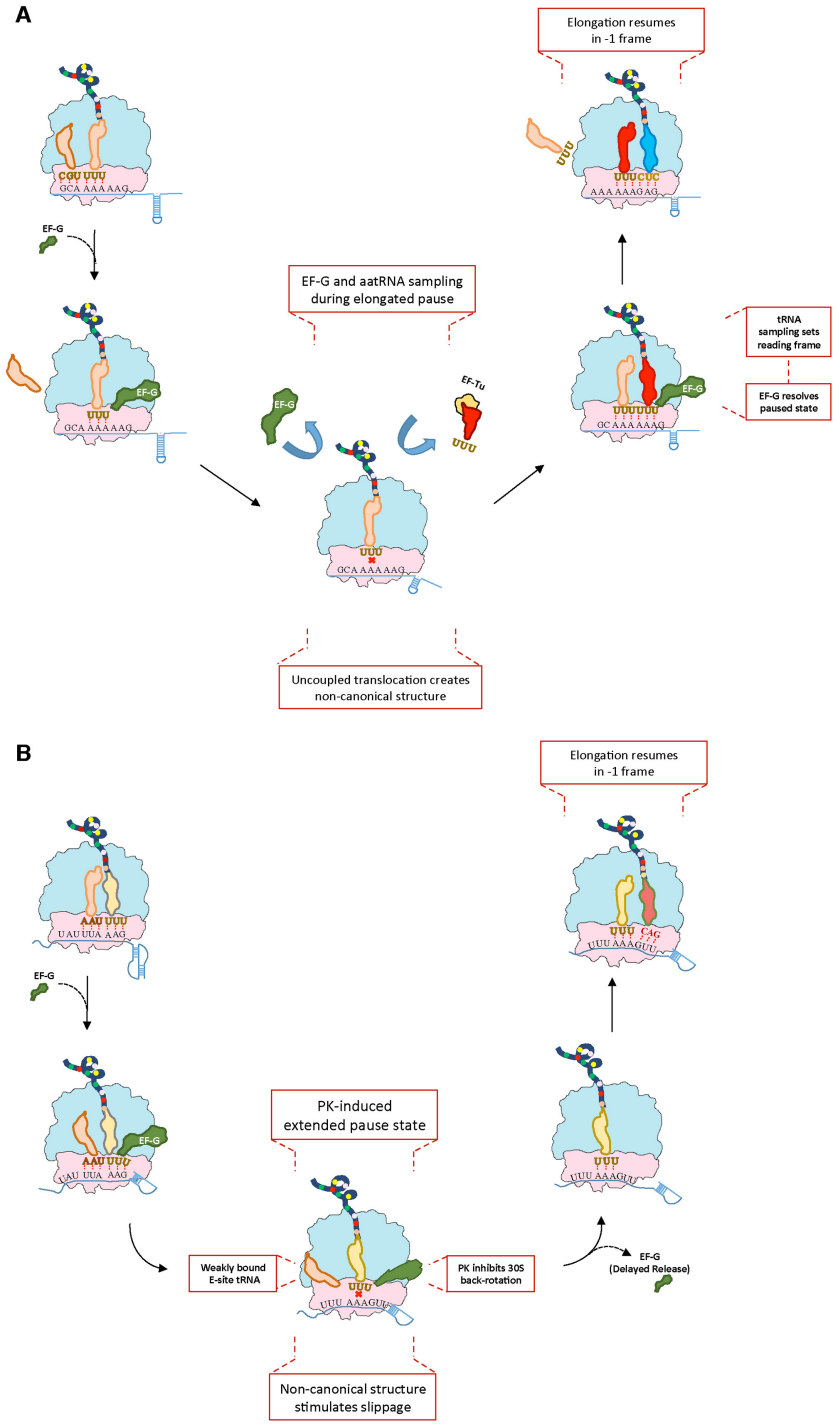
Model suggesting −1 slippage during uncoupled translocation. smFRET experiments on a *dnaX*-derived motif show that slippage occurs during a state of single tRNA occupancy, where the P-site tRNA^Ala^ remains after translocation is uncoupled from E-site tRNA exit in an elongated pause state. This leads to a non-canonical structure of the ribosome where EF-G samples the ribosome >5 times per codon, in parallel with A-site sampling by the incoming tRNA^Lys^. tRNA^Lys^ sampling defines the reading frame, and the non-canonical state is resolved by EF-G mediated translocation, resuming elongation in the −1 frame.

Studies on the IBV-derived system pointed to dissociation of EF-G being the rate limiting step for the incorporation of the incoming amino acid in the −1 frame. In a *dnaX*-derived system, the stem-loop and SD sequence aid the formation of a paused intermediate where translocation is uncoupled from reverse-rotation and E-site tRNA departure. As E-site tRNA exits, leaving only the P-site tRNA in place, EF-G samples the ribosome multiple times during the extended pause state. EF-G sampling is concurrent with continued Lys tRNA sampling at the A-site while slippage occurs. EF-G then finally catalyzes reverse rotation of the ribosome, resolving the unusual intermediate state to resume elongation in the −1 frame (Figure 16).

The ribosome excursions identified in these studies are thought to involve conformational excursions of the 30S ribosomal head, reflecting sampling of different reading frames. Structural studies have shown that ribosome head tilting rearranges the mRNA binding groove by disengaging the 16S rRNA residues that intercalate on the mRNA. The head swivelling and rotation is presumably relevant to the tRNA pairing issues introduced above. The detailed nature of the non-canonical states and how recoding signals, not just for frameshifting but also for dynamic codon redefinition, influence them seems tantalizingly set to emerge soon.

Once a ribosome has embarked on the frameshift pathway, the nucleotide composition of the shift region determines the outcome. One of these studies on *dnaX* frameshifting found supportive evidence for relevance of ribosomal P-site editing (392). This study used bacterial strain MRE6000, which has an *E. coli B*-like release factor 2, i.e. ‘WT’ (M. O'Connor, personal communication; (582)). P-site editing was initially reported (356,583) in a strain containing a partially defective release factor 2, which, on its own, would make general significance extrapolations problematic (584).

Before the recent application of relatively new biophysical techniques, including that used to identify the progressive slowing over several codons discussed in the nascent peptide section, there were many studies on pausing and emerging results from ribosome profiling are highly relevant. Frameshift generated IS911 transposase activity is ∼200-fold higher when it acts in *cis* than when supplied in trans. Pausing associated with the frameshifting influences *cis* preference presumably by facilitating sequential folding and cotranslational binding of the transposase (190). This in turn influences the (*cis*) preference for transposase to act directly on the IS element that encoded it rather than on other copies of the same element (190). Selective rational is provided by cells potentially having many copies of IS elements, and the need to tightly control transposition. Despite the elegance of this example, early pausing studies proved difficult. For a long time, some considered pausing as the key feature of programmed frameshifting with the nature of the shift site being the main determinant of the outcome of the non-standard event facilitated by the stall. Others hoped that the study of stimulators was not merely a study of different ways to induce a pause, with considerations of potential susceptibility to vacant A-site specific nuclease and tmRNA etc (see the 3rd next paragraph).

Classical pausing studies revealed a complicated picture. Several early studies investigated the relationship between pausing and frameshifting (78,80,585). One major paper on infectious bronchitis virus frameshifting concluded that ribosomal pausing at a frameshifter RNA pseudoknot is sensitive to reading phase but shows little correlation with frameshift efficiency (107). In several cases 3′ structures that do not stimulate frameshifting cause similar pausing (78,107,585) (a subset of these were mutants of stimulatory structures). With the IBV pseudoknot, optical tweezer results reporting a correlation between the force for mechanical unwinding and stimulatory effectiveness (586) were later challenged (541). [A different situation pertains with simpler stem loop stimulators.] High resolution ribosome profiling of infected cells has just been performed but at the resolution of the technique no pausing at the frameshift site was detected though it was detected at other positions in the coding sequence (105). Few studies have focused on structural features that may influence ribosomes in a different way, but one is of an isolated luteoviral frameshift stimulatory pseudoknot. This work suggested that a segment not central to pseudoknot stability may influence frameshifting by direct interaction with ribosome components (524).

**Figure 17.**
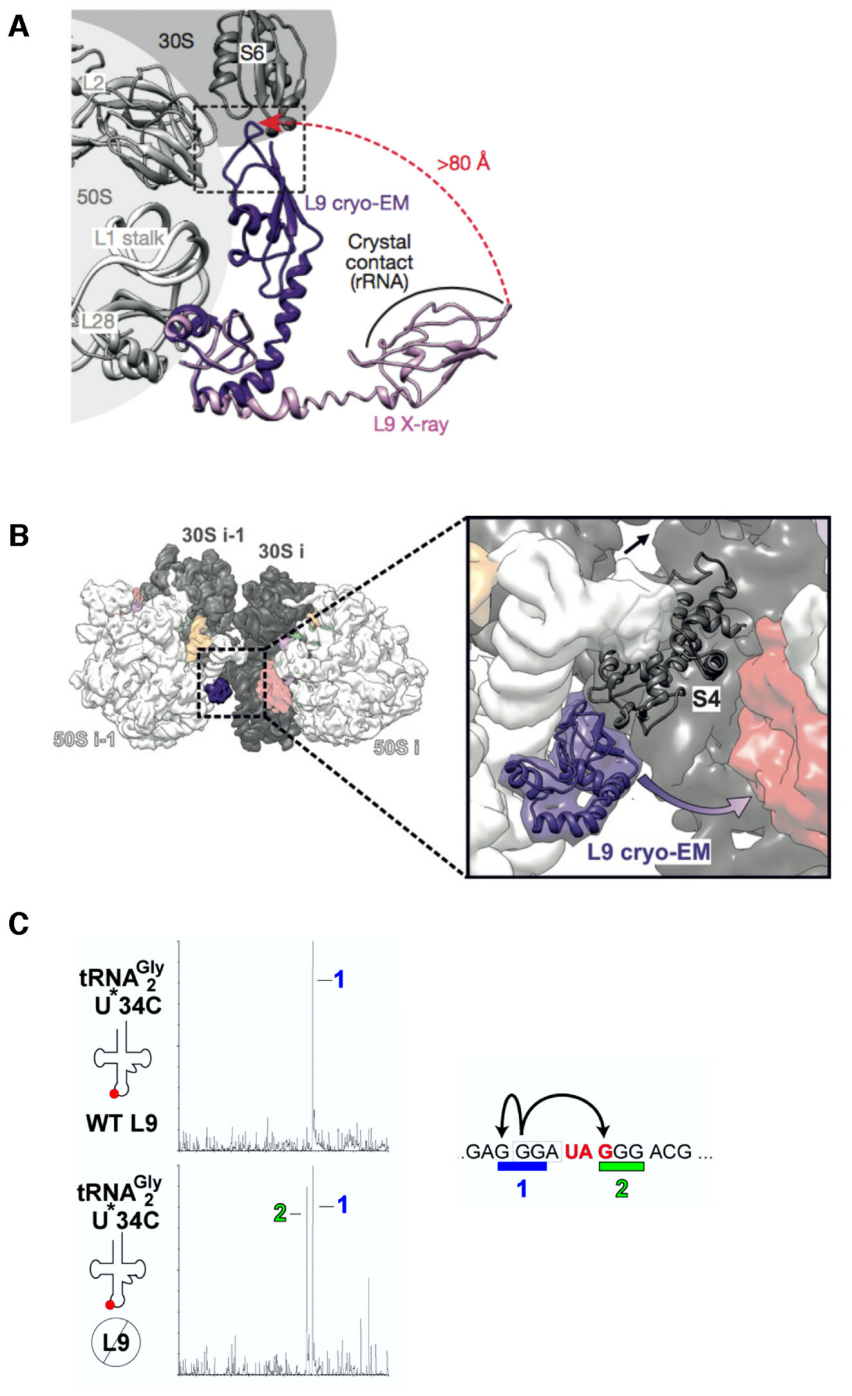
The mobile bacterial ribosome protein L9 that restrains forward mRNA slippage. Its N-terminal domain binds to the large subunit L1 stalk relevant to E-site tRNA egress. (**A**) Cryo-EM visualization of the L9 carboxy-terminal domain contacting ribosomal proteins (L9 is purple) versus the elongated form of L9 (in pink) detected in crystals. In crystals, instead of the L9 carboxy-terminal domain interacting with the 30S subunit of the same ribosome, it interacts with the 16S rRNA of a neighbouring ribosome. Whether this is a crystal artifact or indicative of a ‘strut’ function in polysomes relevant to frameshifting is unknown. (**B**) Model of L9 in the context of polysomes. This shows the arrangement of neighboring ribosomes (i-1 and i) in the major t-t form of *E. coli* polysomes, with the conformation of L9 (blue) revealed by cryo-EM. L9 is located close to protein S4 of the neighboring 30S subunit (30S i) according to the polysome model. The purple arrow indicates the rearrangement of L9 from the cryo-EM conformation to that seen in crystals. The black arrow denotes the location of the mRNA entry channel in the 30S subunit i. Images A and B are from (590). (**C**) Mass spectral analysis of protein products shows evidence for L9 functioning to restrain forward ribosome slippage on mRNA. Inactivation of L9 (lower panel) shows an additional product (2, in green) due to peptidyl-tRNA anticodon dissociation and re-pairing to mRNA at a 3′ position (indicated on sequence at right). The system used had additional mutants that exaggerate the effect. It involved polysomes, and the effect has not yet been tested with low ribosome loading (484) [Reprinted by permission from Macmillan Publishers Ltd: NATURE (Fischer, N., Neumann, P., Konevega, A.L., Bock, L.V., Ficner, R., Rodnina, M.V. and Stark, H., Structure of the *E. coli* ribosome-EF-Tu complex at <3 Å resolution by Cs-corrected cryo-EM, *Nature*, 2015, 520: 567-570), copyright 2015.].

To gain insights into frameshifting, mutants of translation components have been sought that cause altered levels of programmed frameshifting, act as suppressors of frameshift mutants or else were later found to influence them (440,587). A systematic study of this for Ty1 has just been completed (213) and only a few will be briefly mentioned here. Roles emerged for proteins such as Asc1/RACK1 (588), and the mammalian and bacterial ribosomal proteins L4 and L9, respectively (484,589). There is an intriguing connection between L9, whose N-terminal domain binds to the L1 stalk and which stretches via a long alpha helical region 80 Ǻ around the outside so that its C-terminal RNA binding domain can be near S6 on the small subunit (590) (Figure 17), and the bacterial counterpart, EF-P, of hypusine containing eEF5 (591). [The C-terminal domain of L9 restrains forward mRNA slippage (484) (Figure 17) and L9 is required for the growth of EF-P deficient cells (591).] Such studies also pointed to the care needed to avoid indirect effects, though they can be insightful for other processes. Searches for mutants of *S. cerevisiae* that increase frameshifting on an MMTV shift cassette sequence, and independently of L-A virus frameshifting (with loss of a satellite virus that encodes a killer toxin), led to the isolation of mutants of the UPF1 and UPF2 genes (592,593). Follow up work led to controversy that was only resolved with the demonstration that UPF mutants do not affect frameshifting, though they do affect product levels (594,595). [UPF genes are key mediators of NMD (nonsense mediated decay) and their name is derived from ‘up-frameshift mutant suppressor’ to reflect one of the paths taken to their discovery (596).]

### Discontinuous decoding via translational ORF joining: Coding resumption after short distance bypassing and trans-translation

Since the bypassing of 50 non-coding nucleotides in phage T4 gene *60* is, as considered in the nascent peptide section above, so efficient, it has long been presumed that counterparts would exist elsewhere – perhaps involving shorter distances but with retention of the feature that the landing site 5′ adjacent to the resume site, would match the take-off codon. Work with synthetic constructs revealed ‘stop-hops’ where matched codons flank a stop and 9 nts specify one amino acid (54), pointed to the significance of slow-to-decode A-site codons. Over-expression, for biotechnological purposes, of a mammalian gene in *E. coli* soon thereafter showed that heterologous expression mediated specific aminoacyl-tRNA limitation led to counterpart hopping over a sense codon (597). Another group studied in-depth the requirements for counterpart bypassing both in starvation conditions and in normal growing cells (598–601). Lack of follow-up on reports of a 29 nt translational hop in decoding of an adhesion gene of the oral bacterium *Prevotella loescheii*, that is of dental interest (602), was partly due to difficulty in getting it to function in *E. coli*. The possibility of hopping in the expression of *Leishmania* RNA virus LRV2-1 was considered (603) but not investigated. Much work was required to show that two separate reports of bypassing utilization were spurious (604,605).

The paucity of known occurrences of bypassing utilization was overturned by the 81 instances of the translational ‘correction’ of short blocks of nucleotide inserts in decoding mitochondrial mRNA of the yeast *Magnusiomyces capitatus*. Though involving an unused codon in the ribosomal A-site, this bypassing seems to be independent of the mRNA context and reflects a substantial relaxation of mitochondrial coding constraints (56,65). The absence of perfectly matched take-off and landing codons in several cases and perhaps a greater propensity for codon: anticodon dissociation, are likely related to relaxed wobble rules with two-out-of-three base pairing and features of mitochondrial ribosomes (56,65), for which earlier studies with *Mycoplasma* are relevant (412). Studies of other bypass candidates (56,217) are awaited.

Since both the *M. capitatus* and T4 gene *60* bypassing serve to avoid otherwise inactivating inserts, it would broaden interest to discover instances where bypassing serves a regulatory function. The best candidates so far are in *Streptomyces*, where usage of UUA is distinctive (162). The suggestive findings with *Streptomyces* phages (161), give credence to this possibility.

In contrast to coding resumption occurring 3′ of the take-off site on the same mRNA, bacterial tmRNA action causes coding resumption at a specific internal site in its own RNA. [This has been termed *trans*-translation to reflect protein synthesis commencing on one mRNA and completing on a second mRNA, with tm reflecting its tRNA and mRNA-like properties (606).] Pairing of peptidyl tRNA to tmRNA is not involved and so there is no involvement of a matched codon. Instead part of a protein that serves as an anticodon mimic, and certain features of sequence moderately close, but not adjacent, to the resume site are involved in resume site selection (607,608).

In addition, another situation that also provides a stark contrast to the topics being reviewed here, merits mention as well. In certain circumstances when translating mammalian ribosomes encounter no A-site mRNA nts (or 3′ mRNA), polymerization of multiples of two amino acids can occur (609). Bacterial tmRNA also commonly functions with no A-site mRNA nts, but can also do so with several mRNAs occupying part of the length of the ribosomal mRNA entrance channel (610).

### RNA polymerase and Reverse transcriptase slippage: sites and stimulators

**Figure 18.**
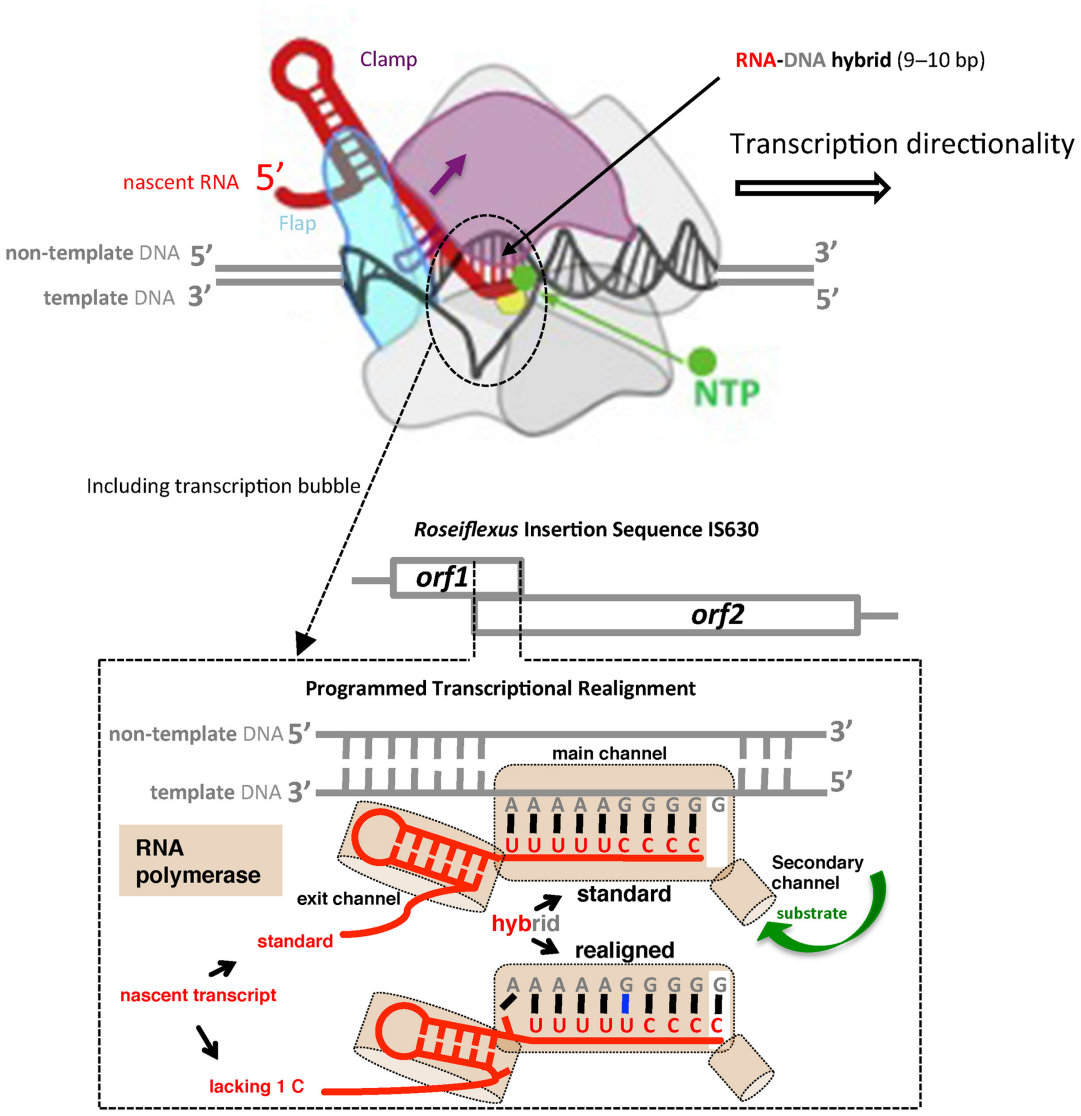
Nascent RNA stem-loop formation stimulates DNA-dependent RNA polymerase slippage. In the top segment (adapted from (614)), the transcription bubble with the unpaired dsDNA and the 9–10 bp RNA–DNA are shown within the dotted ellipse. The position of the polymerase active site is marked in yellow and the incoming NTP substrate is in green. The RNA exit channel is between the Flap domain (blue) and the clamp domain (purple). The lower segment illustrates the nascent RNA stem loop dependent transcription slippage required for synthesis of the transposase of a *Roseiflexus* insertion sequence (IS). The DNA template strand slippage motif, 3′A5G5, hybrid with the growing RNA U5C4 in the post-translocated state with the catalytic center positioned at the 5th G. Formation of a nascent RNA stem loop adjacent to the hybrid and within the mRNA exit channel, has been proposed to melt the upstream part of the hybrid (99) and to open the polymerase clamp (614). In addition to stimulatory effects on forward realignment of the 3′ end of the RNA with respect to the template, the stem loop can also potentially stimulate the slippage by preventing RNA polymerase backtracking and favoring forward polymerase translocation.

**Figure 19.**
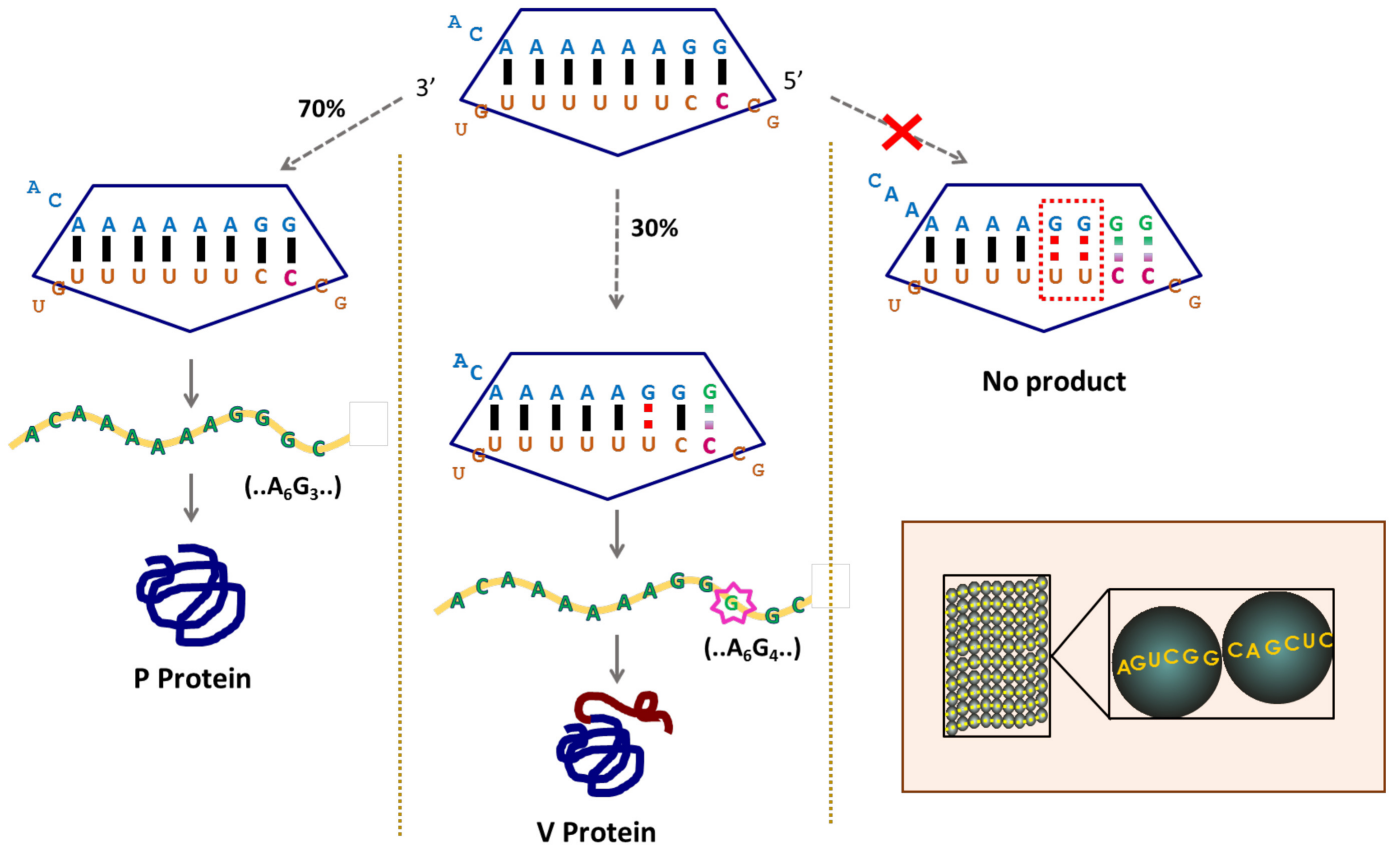
Programmed transcriptional frameshifting in members of the *Paramyxovirinae*. In the P-gene mRNA of Sendai virus, the insertion of a G occurs over the minus strand template RNA slippery sequence UUUUUUCCC. In 30% of the population (middle panel), a pause in RdRp over the slippery sequence promotes slippage of a G-C bond to form G:U pairs (in red dotted lines). Polymerization resumes by addition of a G (in green) over the critical C (in pink), thus encoding mRNA for the V-protein in an overlapping translational −1 frame compared to genomic template. The panel to the right shows that the potential insertion of two G's has very poor likelihood due to multiple G-U pairs not being tolerated. Inset: Hexamer phasing in members of the *Paramyxovirinae*. The ribonucleoprotein (RNP) structure is a left-handed coil where each nucleocapsid protein binds precisely 6 nucleotides.

The following is intended to complement the contents of a different type of review (611) of this topic. In contrast to triplet tRNA anticodon: codon interaction, the hybrid lengths central to DNA dependent RNA polymerases, and RNA dependent DNA polymerases, are much longer and of course without the counterpart of an adjacent tRNA (*Thermus thermophilus* RNAP hybrid oscilates between 9 and 10 bp during forward and backward translocation on DNA). Post initiation region slippage by bacterial RNA polymerase was initially detected *in vitro* (612) and shown to occur with high efficiency *in vivo* with a run of 9 As or Ts (613). Productive utilization of slippage at 9 Ts in the *Thermus thermophilus dnaX* gene yields a heterogeneous population of mRNAs with 1 or multiple additional As in a diminishing proportion such that standard translation yields about 50% of the product being full length (323). Without context features, slippage is not detectable at TTTTTCCCCC (T5C5). However, an IS element from *Roseiflexus* bacteria utilizes context-dependent slippage at this sequence to generate transcripts lacking a C residue within the corresponding U5C4 mRNA. Standard translation of these mRNAs yields its transposase (99). The enabling context feature is formation of a nascent RNA stem loop structure from the inverted repeat sequence, GCGGGCgcaaGCCCGC 5′ adjacent to the U5C5/U5C4 motif (99) (Figure 18). [This is just one of the types of regulatory events that can come from RNA polymerase: nascent RNA structure interactions, with RNA polymerase progression involving a ‘rugged kinetic landscape’ (614).] This programmed unidirectional specific slippage occurs uniquely at the C4 position of the T5C5 motif. Inviability of more than one rU:dG and even one rC:dA mismatch prevents a two-base deletion and any insertions, respectively, making it quite distinct from the slippage on 9 Ts in *T. thermophilus dnaX*. While transient realignment occurs on a broad variety of heteropolymeric sequences, rapid reversal prevents deletions and insertions in the mRNA unless additional sequence elements that inhibit the reversal are present. *S. cerevisiae* RNA polymerase can also slip on a cassette with the same T5C5 sequence (99). The proposed mechanical model involving the RNAP translocation state differs from that proposed for *Paramyxovirinae* ‘programmed transcriptional frameshifting’, where, following random movement, efficiency is mainly dependent on the stability of the new realigned hybrid. The Sendai virus slippage sequence is 3′ (Uug)UUUUUUC**C**C 5′ and 30% of the mRNA synthesized from the P-gene has an additional G residue that causes ribosomes, by standard translation, to enter the coding sequence for the host-defense inactivating V protein. This is the sole Sendai virus alternative product to P, an essential viral polymerase co-factor, for synthesis that initiates with the P-gene start site (615). However, with parainfluenza virus type 3 there is an additional product due to the realignment within its P-gene yielding separate RNAs that permit standard translational access to both alternative ORFs. This realignment involves 1 to 6 Gs being added with approximately equal frequency. Most of the relevant features are in its (Uaa)U_6_C_3_ slippage sequence and when Sendai's UugU_6_C_3_ is replaced within the Sendai virus context by UaaU_6_C_3_, the slippage becomes parainfluenza virus type 3-like. The relevant context feature is the phasing of the binding of multiple nucleocapsid proteins (N) that sheath the genomic negative-strand (and positive-strand antigenome) of these viruses. Such binding prevents complementary progeny positive-strand mRNAs from annealing with the genomic RNA. Each adjacent N protein binds precisely 6 nts of genomic RNA (615), and adjusting the phasing of hexamer binding to two particular phases, causes the pattern of G inserts in progeny RNA to revert to the Sendai-virus pattern. Even when N is displaced from the genomic RNA by polymerase, it apparently remains closely associated with the polymerase and its hexamer phasing is thought to influence polymerase pausing at the slippage site with realignment consequences (616,163). The genome length is such that there is full phasing coverage and for the different species their respective phasing number is coordinated with that required for realignment by the relevant polymerase (Figure 19). N binding is also relevant to avoidance of G insertion genomic fixation, with deleterious consequences for P protein synthesis, as only genomes with length an exact multiple of 6 nts are efficiently replicated (616). A few paramyxoviruses, including Nipah virus, a member of the less well known *Henipavirus* genus, exhibit hyper transcriptional slippage, with some unresolved mechanistic issues (617,618).

Realignment in transcribing the genome of Ebola viruses (genus *Ebolavirus*) is different and with a significant issue remaining unresolved (99). Unlike paramyxoviruses, the precise length of Ebola virus genomes is not constrained and different lengths of the realignment site have been reported on transition between guinea pigs and tissue culture cells derived from a different host (619,620).

In plant potyviruses, the polymerase realignment that permits synthesis of the extra protein P3N-PIPO, yields about 2% of the transcripts having an extra A in the sequence GAAAAAA (42,44). For these viruses, a distinctive mechanism for avoidance of replication of slippage-derived transcripts has been proposed (42). It is based on translation of a region near the 3′ end of the polyprotein ORF being required for the genome to be used *in cis* as a replication template (173). Ribosomes translating slippage-derived transcripts will encounter stop codons before reaching that region, putatively precluding RNA with the extra A from being efficiently replicated. Though the slippage-derived RNA is 1 nt longer than the genomic RNA, it is the functional equivalent of a sub-genomic RNA in that it is (putatively) not replicated and is template for the synthesis of a subset of viral products. Since in positive-sense RNA virus transcription, unlike in negative-sense RNA virus transcription, the template: nascent RNA duplex is likely to extend a considerable distance behind the RNA polymerase footprint (possibly only being dissociated when the next polymerase passes), potyvirus slippage may require formation of a bulge nucleotide (42).

### Growth or developmental phase, carbon source

A few experiments have compared frameshifting rates under different growth phases. Some bacterial frameshifting is known directly or by inference, to greatly increase when cells enter stationary phase (17,202,621–623). Synthesis of a tRNA relevant to a *Streptomyces* phage candidate bypassing (161), is mainly restricted to the stationary phase that is irrelevant to phage infection (162). Dependence of some plant (e.g. luteovirus), and animal (cardioviruses and HIV), viral frameshifting on viral life cycle stage or cell status is described above. Frameshifting to synthesize *S. cerevisiae* Ty1 and/or its close relative Ty2, GagPol changes in a growth-stage dependent manner (624,625). Cell cycle stage relevance of EST3 and antizyme frameshifting is also described above.

Ty1 frameshifting decreases 4-fold in glycerol-lactate grown cells compared to cells grown in media with the preferred carbon and energy source, glucose (625), and glucose level effects with bacterial IS1 have also been reported (623). Ty3 frameshifting efficiency varies up to 10-fold depending on the carbon source, but in contrast to Ty2, it is lower in glucose grown cells than in poorer carbon-source media (625). Previously it was shown that Ty3 Pol and transposition decreases dramatically with high temperature or growth in ethanol (626) where the frameshifting rate is greatly reduced (216). (Possible cold shock effects have in general not been studied to date.) Genetic studies strongly indicate that under growth in the poorer carbon sources, one or two kinases phosphorylate the ribosome-associated protein Stm1p which in turn affects the Ty3 frameshifting level (216). Stm1p is involved in the regulation of eEF3, a yeast-specific elongation factor that facilitates binding of aminoacyl-tRNA to the ribosomal A-site. How many other cases of retrotransposon frameshifting are responsive to alterations of the translation elongation process is a significant unresolved issue. Perhaps mechanistically quite distinct, but an indication of the potential diversity is that there is elevated *D. melanogaster* 1731 retrotransposon *gag-pol* frameshifting in males compared to females (627).

Several papers have addressed the potential for altered modification status of the frameshifting relevant tRNAs during the course of retroviral infection to occur and relate to *gag* frameshifting efficiency (628–630). It is clear from two major studies and earlier work cited that in bacteria certain tRNA undermodification enhances frameshifting (391,631) and metabolism and growth conditions influence modification levels (632). Of relevance to ribosomal frameshifting at U-rich sequences are studies in thermophilic archaea (633,634) and in *Drosophila* (635) which have shown organism-specific differences in the degree of conversion of 1-methyl G to wye in the tRNA^Phe^ anticodon loop. Studies in *S. cerevisiae* with the frameshifting utilized by the double-stranded RNA virus L-A found increases with reduced wye modification of tRNA^Phe^ leading the authors to wonder if the degree of modification in some organisms is related to finely tuned tRNA^Phe^ -dependent frameshifting (636).

### Alternatives to frameshifting and combinations with other types of recoding and interchangeability of recoding signals

The regulatory frameshifting required for bacterial release factor 2 and eukaryotic antizyme synthesis has not been substituted in any known organism with regulatory dynamic stop codon redefinition (stop codon readthrough) involving ORF2 being in the same frame as ORF1. However, in a number of retroviruses, including Moloney murine leukemia virus (MuLV), GagPol is synthesized via in-frame readthrough of a UAG stop codon (92,93) rather than via frameshifting. It uses a stimulatory pseudoknot that is only rather ineffectively substituted by its counterpart mouse mammary tumor virus frameshifting stimulatory pseudoknot even though they are similarly positioned 3′ of the recoding sites (637). Substituting instead the HIV frameshift stimulatory signals resulted in readthrough levels only 2- to 3-fold lower than WT (638). Experiments with two plant viruses that utilize readthrough to express their RNA dependent RNA polymerase, showed that recoding signals of one of them, carnation Italian ringspot virus (genus *Tombusvirus*) was partially functional for stimulating frameshifting whereas that of the other, tobacco necrosis virus D (genus *Betanecrovirus*), was not (639). The similar recoding signals are bipartite and analogous to frameshifting stimulatory counterparts in barley yellow dwarf virus (genus *Luteovirus*) (110) and red clover necrotic mosaic virus (genus *Dianthovirus*) (554) with the 4 kb distant pairing.

Despite the above, a lot remains to be learned about the extent to which stimulatory signals are switchable between the different types of recoding. One whose spacing and structural comparision with 3′ stem loop mediated frameshifting that would be especially interesting is that utilized by the Hymenoptera SPS1 gene (640) and counterparts in a subset of selenoprotein mRNAs (641,642).

Different types of recoding may have sequential, or possibly even coordinated, functions. Efficient frameshifting in cardiovirus decoding, especially of the theiloviruses, serves to divert a large proportion of ribosomes to quickly terminate so that only a small proportion continue to synthesize the enzymatic proteins. This frameshifting occurs very close downstream to a StopGo sequence that results in release of the upstream structural components (58,59). While in that case the StopGo sequence appears to have a coordinated function, in certain cases involving a secretion signal, it has been selected to itself lead to termination of a significant proportion of ribosomes (643).

Just one of the non-recoding mechanisms that can, in some cases, have parallels to a type of recoding, is shunting by scanning 40S subunit complexes en route to initiation. The parallel in that case is with whole translating ribosomes undergoing bypassing/ hopping.

In conclusion, and as expected, frameshifting is no exception to the continuum of biological processes and ‘fuzzy’ boundaries between different categories.

### Distribution of shifty sites in whole genomes: Contrast between highly and lowly expressed genes; Occurrences detected in phage display; Finding undetected cases of productive frameshifting

The finding that much programmed −1 frameshifting occurs at specific heptanucleotide shift sites prompted searches for unknown occurrences of such heptanucleotides as a way to find candidates for new cases of utilized frameshifting. The follow-up by members of the same group to the pioneering study by Hammell *et al*. (644) in *S. cerevisiae* is described above. A different type of search focussing on +1 frameshifting in *S. cerevisiae* had the goal of finding the most under-represented heptanucleotides on the basis that they may reflect the most efficient frameshift sites and so would be selected against except where utilized. This revealed several candidates (424).

**Figure 20.**
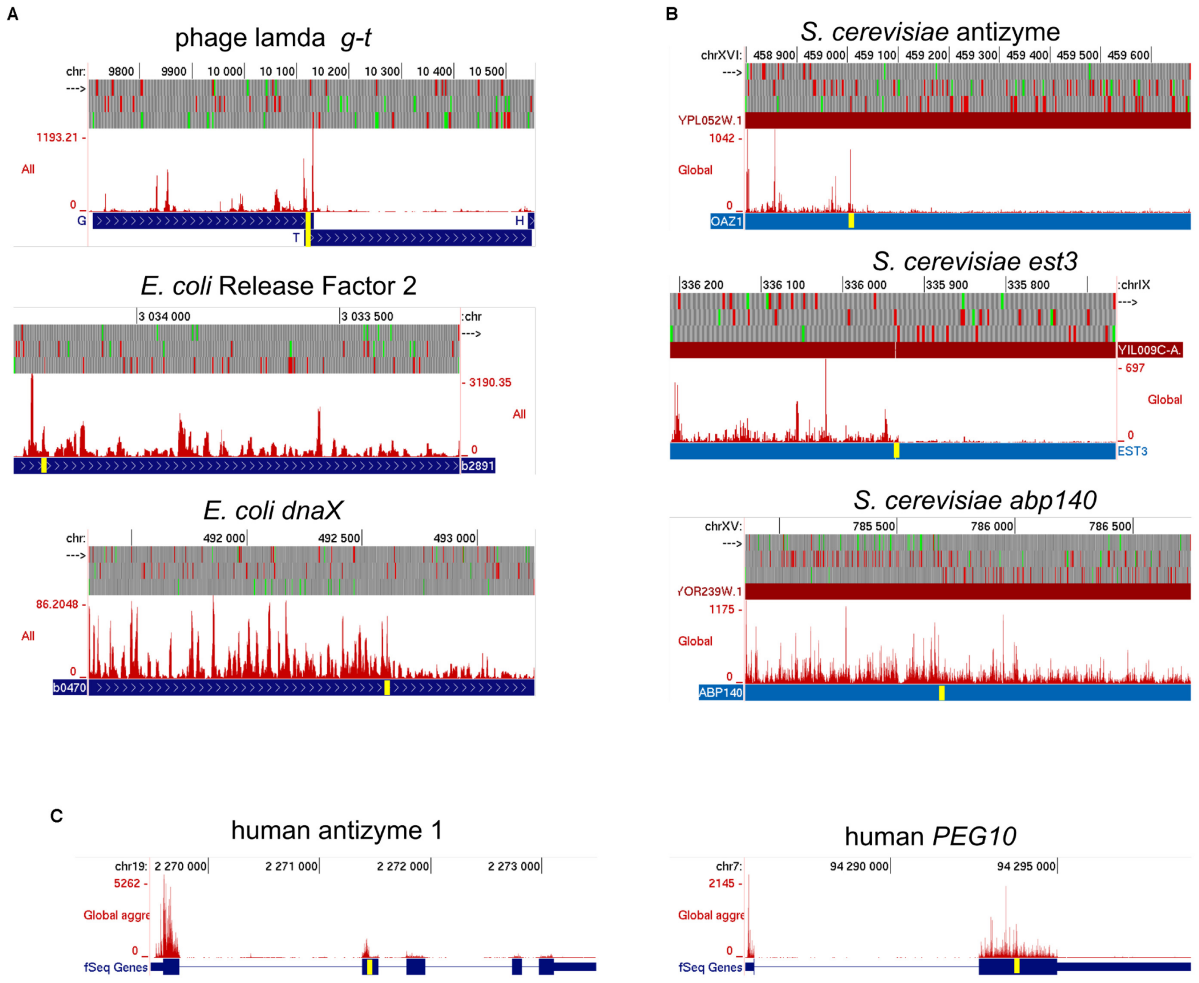
GWIPS-viz (816) screenshots of profiles of ribosome footprint densities. Expression of the selected genes involves ribosomal frameshifting from (**A**) bacteria, (**B**) yeast and (**C**) humans. Blue bars at the bottom represent annotation of protein coding genes according to the corresponding genomes (A and B) and RefSeq (C) with yellow boxes indicating positions of frameshifting sites. The density of ribosome footprints is shown as red columns corresponding to inferred positions of the A-sites. The top plots (A and B) show ORF organisation in genomes (green ticks are for ATG and red for stop codons). For more details on notations used see GWIPS-viz browser at http://gwips.ucc.ie

Ribosome profiling studies performed with certain ribonucleases provide framing information that allows illustration of known frameshifting cases (Figure 20). Framing information also allows for the potential to identify new cases of frameshifting.

The energetic cost for the cell of occurrences of the most shift-prone sequences where frameshifting at these sites is not productively utilized, will in general be greater in highly expressed genes than in lowly expressed genes. Since only a modest proportion of genes are highly expressed, confidence in assessments of significance of the number of reading frame transitions in them is less than in lowly expressed genes. Shift pattern under-representation featured prominently in an analysis of occurrences of A_AAA_AAG in *E. coli* where it is one of the most ‘shifty’ sequences. It is only moderately selected against, though it is absent from the 306 most highly expressed genes (464). In lowly expressed genes, quite a number of occurrences of unselected cases of frameshifting appear to have a marginal effect at most and may be effectively of neutral consequence (464). A further study of 7 *Shigella*, 1 *Salmonella* and a number of *Escherichia* genomes found AAAAAAA and UUUUUUU, to be underrepresented in all three frames (195). The poor frameshifters C_CCG_GGC and C_CCG_GGU were also found to be underrepresented and U_UUC_CCG to be markedly overrepresented. However, as considered in that publication other factors related to particular pairs of codons are also relevant.

A surprise for some early practitioners of phage display was that some active encoding sequences had frame disruptions. For instance, biopanning of a random peptide library on a filamentous phage for sequences that would bind to a growth hormone binding protein yielded sequences whose decoding was deduced to undergo substantial levels of either +1 or −1 frameshifting (645), and corresponding results have been seen in other phage display studies (646,647). Recent systematic exploration of shift-prone sequences in *E. coli* (195), and searches in other bacteria (198) is relevant to the anticipation, and understanding of, such results.

### Databases and annotation

Several databases related to frameshifting have been developed (648–654) as well as software for the identification of frameshifting (24,348,655–657,658). Major reference sequence databases have started with antizyme to make impressive efforts to annotate ribosomal frameshifting (274), but much more is needed.

### Vector systems and assay cautionary tales

Vectors whose products permit estimation of the proportion of ribosomes that shift from translating the zero to the alternative frame of interest have provided many insights to frameshifting. While they avoid some comparative problems with solo reporters, they have different ‘issues’. Commonly the 5′ end of the frameshift cassette being tested is fused in the zero frame to the 3′ end of the reporter used to reflect zero frame decoding, and its 3′ end is fused to a downstream reporter such that its decoding monitors new frame reading. When the 5′ encoded reporter is assayed by enzyme activity, care is needed to avoid or be cognizant of, distortions due to features of its C-terminal extension though for the 3′ encoded reporter its being on the C-terminal side of a trans-frame encoded fusion product can also be relevant. Vector systems where both products can be assayed in the same tube, such as firefly and renilla luciferase (659) have proved popular, though fluorescence reporters, e.g. (660,661), have been gaining ground. Plasmid-based expression has been very valuable, but in certain cases can yield over-expression artifacts avoidable with chromosomal integrants (427).

Many studies have used only modest-length sequence flanking the known or suspected, frameshift site and in some cases this led to misunderstanding. It has taken clever sleuthing to reveal the very important distant-acting features in luteoviral, HIV, herpes virus drug resistant mutants and *S. cerevisiae* antizyme frameshifting described above. In contrast, there has been general awareness of the need for caution in (i) extrapolating from *in vitro* to *in vivo* or when heterologous expression is involved and (ii) regarding *E. coli* growing in rich lab media as reflecting most bacterial growth in nature. Undue proximity to the initiation site for practical reasons in certain early studies was an issue, and issues relating to mRNA stability, ribosome spacing level and distinction between transcriptional and translational ‘frameshifting’ are also now more addressable.

### Manipulation of Recoding and importance of frameshift efficiency: Intervention in natural situations and toward synthetic biology

Perturbation of frameshift efficiency is potentially beneficial. While there have been some studies with regulatory frameshifting, for instance testing polyamine analogs on antizyme frameshift efficiency (662,663), reasonably the great majority of studies have focused on a set ratio (especially that utilized by viruses and in particular HIV).

When the level of *S. cerevisiae* Ty1 frameshifting was reduced by over-expressing the relevant limiting A-site tRNA, the transposition frequency was greatly reduced (664). Also when retroviral *pol* was brought into the same frame as *gag*, inviability ensued (665). Early studies on perturbation of HIV *gag-pol* frameshifting with paromomycin (666), inhibition of human T-cell lymphotropic virus gag-pro frameshifting with cycloheximide (667), inhibition of *S. cerevisiae* double-stranded RNA virus −1 frameshifting by anisomycin and preussin (668,669) [Addition of anisomycin reduced the −1 frameshifting and toxicity of cells stably transfected with a cassette of disease-associated expanded CAG triplets of spinocerebellar ataxia 3 sequence (374)], and further HIV studies (670–672) were accompanied by numerous comments, including (673–676) on the potential of targeting viral frameshifting. Essential for any such targeting is knowledge of the extent of effects of various levels of frameshift perturbation on virus propagation. This was first systematically investigated with the *S. cerevisiae* double-stranded RNA virus, L-A (79). In addition to studies on *S. cerevisiae* Ty elements (677), this was followed by several studies on counterpart significance for several viruses, including Rous sarcoma virus (549) and SARS coronavirus (546,678,679), but especially HIV (660,680,681,676,682).

Further to ‘off the shelf’ translational inhibitors, with more of an eye to the recoding signals or flanking sequence, compound libraries have been screened and oligonucleotides synthesized. The best candidate from the initial screen for compounds targeting the 3′ recoding signal for HIV frameshifting (683) was later shown to bind to a variety of RNA stem loops (684). A new screen and subsequent modification has yielded a compound with much greater specificity, and yet causes a 50% increase in frameshifting (685). Other approaches to increase specificity have been followed including recent modification of RNA binding peptides (686). A detailed review on this topic is available (682). Other studies have been on the pokeweed protein (687) and on ligand binding to a riboswitch (688). The latter highlights the potential of frameshift stimulators as bricks in synthetic biology to engineer gene expression bifurcations.

Oligonucleotide targeting to the HIV frameshift 3′ stimulatory structure has also been investigated (689). However, the main motivation of synthetic oligonucleotide work has been for the mechanistic insights it can provide and as part of an exploration of different potential ways of being able to generate frameshifting that could partially compensate for a nearby genetic disease causing frameshift mutation. Modified synthetic oligonucleotides complementary to a sequence 3′ of known or potential frameshift sites lacking 3′ intra-mRNA structural frameshift stimulators were tested for their potential to stimulate frameshifting (98,690–693). They showed unexpected spacing features. It is unclear if these findings presage natural *trans*-acting mRNAs (the miRNAs that influence CCR5 frameshifting bind to 3′ structural stimulators (27) rather than to unfolded mRNA). However, no practical reagents of this type for causing compensatory frameshifting for frameshift mutation mediated human genetic disease have yet been developed. Effects of synthetic oligonucleotide binding 5′ of shift sites has also been studied (505).

A counterpart to targeting frameshifting with therapeutic intervention is the use of agents such as antibiotics for their crucial antibacterial effects, but which can have low-level effects on frameshifting even with mammalian ribosomes. While the large battery of proteases would rapidly destroy most proteins, this may not apply to all whether abberant or normally utilized, with potentially significant effects in special cases perhaps even for neurodegenerative disease (369). Treatment of *Clostridium perfringens* infected horses with gentamycin or streptomycin caused in-frame restorative frameshifting in the decoding of a cryptic gene whose product is involved in the synthesis of a toxin that causes more accentuated and fatal progression of equine typhlocolitis (694). At a substantial majority of frameshift sites, streptomycin does not cause detectable frameshifting, but at some it does (671) and is not surprising (369), due to aminoglycosides causing increased acceptance of near-cognate aminoacyl-tRNA and reduced acceptance time (402,695). However, even gentamycin and paromomycin which in general are more effective than streptomycin, commonly cause only very modest increases in frameshifting (696). The oxazolidinones, linezolid and especially R chi-01 promote more but still often modest levels of frameshifting (696,697).

The most significant discovery to date of antibiotic mediated frameshifting is the regulatory frameshifting caused by members of the ketolides group of macrolides, during the decoding of a ‘leader’ uORF of a gene encoding a protein conferring erythromycin resistance (61,698).

There have been two recent studies pertinent to synthetic biology (699,700). One generated synthetic ligand-responsive stimulators for eukaryotic −1 frameshifting at U_UUA_AAC. To design them the authors of the second study used mRNA display to select the most efficient stimulators from over 200 million sequence variants. Further they modified the winners by coupling them with a ligand-responsive riboswitch followed by optimization in the course of directed evolution *in vivo* (in yeast). The authors demonstrated the applicability of such frameshifting-based sensors by designing genetic circuits that act as Boolean logic gates which trigger cell death in response to the combinatorial presence of two ligands, theophylline and neomycin (700).

### Do frameshift-derived peptides perform a role in immune display?

Major Histocompatibility Complex (MHC) class I molecules bind and present oligopeptides at the cell surface. This presentation of peptides enables lymphocyte-mediated immunosurveillance important for viral and tumor immunity. It is upregulated by interferons and certain other cytokines. In addition to peptides being derived from mature ‘retiree’ proteins, a proportion of the peptides come from proteins being synthesized, or directly afterward. Part of the initial reason for appreciating this came from the rapid time after viral infection that infected cells display viral antigens recognized by specific CD8+ cytotoxic T-lymphocytes, with viral counter-attack being relevant (701). This led to the DRiP (Defective Ribosomal Products) hypothesis (702) which was later broadened to include products derived from ribosomal frameshifting (703,704), transcriptional indels (705), various other non-standard events and notably potential specialized ribosomes ‘immuno-ribosomes’ (706). Detection is highly sensitive and antibiotic treatment can affect such peptide display (707). There are numerous fascinating and important unanswered questions about the origin of peptides selected for display, and the extent of relevance of cotranslational protein degradation (708) and of frameshifting are only a couple of the unknowns.

## HISTORY, ORIGIN AND FUTURE PERSPECTIVES

Reading of successive non-overlapping triplet codons is so ingrained in our perception of decoding, that it is easy to gloss over the dilemmas faced in the 8 years prior to 1961 in trying to imagine and distinguish between a variety of possible decoding schemes. However, from the discovery of the general nature of decoding (159) until two years after completion in 1966 of the deciphering of codon assignments, the pendulum swung too far and it was thought that framing was so hard-wired that frameshifting did not, and could not be made to, occur. The practical manifestation at the time, some ‘ten’ years in advance of the development of DNA sequencing, was the perception that a mutation could not be a frameshift mutation if it was leaky (allowed the synthesis of some full-length product), or could not be suppressible by a secondary ‘suppressor’ mutation of some translational component, i.e by mutation of a gene at a separate location from the gene containing the original frameshift mutation (709–711). These misconceptions of sacrosanct framing were corrected with the discovery of (i) frameshift mutant extragenic suppressors (712,713) that, as expected, were later shown to be mutants of various translation components that compensate for a framing ‘problem’ (439,440), (ii) frameshifting by the WT translational apparatus as evidenced by frameshift mutant ‘leakiness’ (393,714) and (iii) the first identified viral encoded frameshift products (73,74). However, it took the advent of general availability of synthetic oligonucleotides of specified sequence and more extensive sequencing in 1984 to facilitate the discoveries that brought utilized frameshifting to widespread interest. While this occurred in 1985 with yeast Ty (206,715), release factor 2 (350) and retroviral frameshifting (76), follow-up work was needed to show at what level it occurred, see above, by which time coronaviral frameshifting was discovered (103). Parallel developments with other phenomena permitted appreciation of common features involved in various ways of utilizating non-standard decoding, and the dynamic competition with standard decoding. This led to a new word, recoding or reprogrammed genetic decoding (716–718). In contrast to this use for naturally occurring organisms, the same word was later used somewhat differently in connection with human intervention to ‘genomically recode organisms’ (719), though several of the authors involved are now using a different term for that meaning (720). [Distinctions between natural recoding and complete reassignment of the meaning of a codon wherever it occurs irrespective of context, have been highlighted (721) while word usage evolves, clarity about the intended meaning is key.]

Primitive protein synthesis is unlikely to have been strictly linear but to have had dissociation and re-pairing to mRNA at new codons and other translational ‘latitude’. Not only was there presumably a lesser ability to restrain such activity, it could have been useful in partially circumventing problem deficiencies in substrate supply. Irrespective of whether one envisages a small proto-tmRNA-like structure being relevant to primordial protein synthesis (722,723), since glycine was likely one of the early amino acids (724), it is possible that Shine Dalgarno-like interactions served to restrain mRNA ‘drift away’ with G-rich mRNA sequences being present. Did Shine Dalgarno mRNA:rRNA pairing play a role in the elongation stage of protein synthesis before serving a role in initiation (725)? Whether any extant productively utilized frameshifting predated those cases derived from triplet decoding where a framing error was selected and its efficiency enhanced with recoding signals, is unknown (at least it seems likely that frameshifting was utilized in expression of the common ancestor of release factor 2 (355)). However, it is not for us to tell nature what is ideal – ‘programmed error’ would be an inappropriate descriptor that would detract from the illustration of decoding versatility provided by the mechanisms used for frameshifting and other types of recoding.

To minimize the need for a proto ribosome to stabilize triplet codon: anticodon interactions, Crick and colleagues developed a proto tRNA anticodon alternating stacking scheme that allowed for quintuplet pairing but only triplet decoding thus avoiding destruction of previously coded information on transition to triplet only pairing (726). Despite the elegance of this scheme, several of the different models for the origin of decoding are relevant for those with the mindset of a less sacrosanct view of framing and an appreciation that Crick's frozen accident thesis (727) has considerably melted (728). These models involve stereochemical interactions between amino acids and RNA, proto mRNAs evolving as linkers between peptidated RNAs and tRNAs derived from replicators involving parallel duplication (729–731). Further to the framing latitude considered in the last paragraph due to dissociation and re-pairing, there has been consideration of greater than triplet codon: anticodon interactions by proto tRNAs (722,723), and perhaps even of tRNA mimics (486). While at least the great majority of frameshifting with WT translational components considered above involves at least one tRNA anticodon dissociating and re-pairing in a new frame, that of Ty3 has been proposed not to do so (214) and there is considerable doubt about a case of non-cognate frameshifting (452,453). Similarily among the frameshift mutant suppressors that have altered tRNAs, though some involve anticodon racheting, in at least the great majority the evidence points to no more than 3 anticodon loop nts being paired to mRNA at one time (440,732).

Tinkering with decoding versatility (733) is no longer just Nature's prerogative. Despite flexibility of tRNAs with expanded anticodon loops (734,735), several advances including use of pyrrolysine tRNA, synthetase selection improvements (736), mutants of ribosome decoding cavities (737), genome wide codon reassignment together with deletion of a release factor gene and potentially of subunit tethering (738), are permitting effective use of quadruplet codons as one of the approaches for codon creation in efforts to greatly expand the repertoire of types of encoded amino acids for new synthetic capabilities with accompanying genetic biocontainment and virus resistance (438,739).

Various facets of programmed frameshifting and other types of recoding are likely to feature in the toolkit of future synthetic biology. However, for now the main excitement is the prospect of being able to ascertain the extent to which productive frameshifting is utilized to enrich gene expression and, with the recent advances in biophysical techniques, to at last understand how ribosomes sense and respond to recoding signals. In conclusion the study of frameshifting has matured by reaching the final stage of Jonas Salk's 1958 trajectory for scientific discoveries: *The way an idea eventually becomes an accepted truth is revealed by the stages through which it passes: First, it is said that ‘It can't be true’; then, ‘If true, it is not very important’; and finally, ‘We knew it all along.’* UGA-C.
